# Global prevalence of underweight and overweight/obesity among migrant workers: a systematic review and meta-analysis

**DOI:** 10.7189/jogh.16.04154

**Published:** 2026-05-15

**Authors:** Herlin Ajeng Nurrahma, Hidayat Arifin, Anung Ahadi Pradana, Herry Susanto, Chiu-Li Yeh, Ya-Ling Chen, Hitoshi Shirakawa, Kuei-Ru Chou, Suh-Ching Yang

**Affiliations:** 1 School of Nutrition and Health Sciences, Taipei Medical University, Taipei, Taiwan; 2 Department of Physiology, Faculty of Medicine, Sultan Agung Islamic University, Semarang, Indonesia; 3 Department of Medical-Surgical, Emergency, Disaster, and Critical Care Nursing, Faculty of Nursing, Universitas Airlangga, Surabaya, Indonesia; 4 Research Group in Medical-Surgical Nursing, Faculty of Nursing, Universitas Airlangga, Surabaya, Indonesia; 5 School of Nursing, College of Nursing, Taipei Medical University, Taipei, Taiwan; 6 International PhD Program in Gerontology and Long-Term Care, College of Nursing, Taipei Medical University, Taipei, Taiwan; 7 Mitra Keluarga Hospital Group, Jakarta, Indonesia; 8 Faculty of Nursing, Universitas Islam Sultan Agung, Semarang, Indonesia; 9 Laboratory of Nutrition, Graduate School of Agricultural Science, Tohoku University, Sendai, Japan; 10 Department of Nursing, Taipei Medical University-Shuang Ho Hospital, New Taipei, Taiwan; 11 Research Center in Nursing Clinical Practice, Wan Fang Hospital, Taipei Medical University, Taipei, Taiwan; 12 Psychiatric Research Center, Taipei Medical University Hospital, Taipei, Taiwan; 13 Research Center for Neuroscience, Taipei Medical University, Taipei, Taiwan; 14 Research Center of Geriatric Nutrition, College of Nutrition, Taipei Medical University, Taipei, Taiwan; 15 Nutrition Research Centre, Taipei Medical University Hospital, Taipei, Taiwan; 16 School of Gerontology and Long-Term Care, College of Nursing, Taipei Medical University, Taipei, Taiwan

## Abstract

**Background:**

Migrant workers are increasingly recognised as a nutritionally vulnerable population due to unstable working conditions, disrupted food environments, and limited access to health services. However, global estimates of malnutrition in this group remain fragmented. We aimed to identify the global prevalence of underweight and overweight/obesity among migrant workers and identify key moderating factors.

**Methods:**

We conducted a systematic review and meta-analysis following PRISMA guidelines. We searched seven databases without restrictions on language, region, or year of publication. Observational studies reporting the prevalence of underweight (body mass index (BMI) < 18.5 kg/m^2^) or overweight/obesity (BMI ≥ 25 kg/m^2^) among migrant workers were included. We calculated pooled prevalence estimates using generalised linear mixed models with random effects. We assessed heterogeneity with *I^2^* and Cochran’s Q statistics. Moderator analyses were conducted using meta-regression and subgroup comparisons. Further, we appraised study quality using the Joanna Briggs Institute tools, and assessed the certainty of the evidence using GRADE.

**Results:**

We included 30 studies involving 135 404 migrant workers with a mean age of 32.3 years (standard deviation = 11.1). The pooled prevalence was 4.7% (95% confidence interval (CI) = 3.1, 6.9) for underweight and 43.7% (95% CI = 39.8, 47.6) for overweight/obesity, suggesting a substantial double burden of malnutrition. Underweight was more common among local migrants and in lower-middle-income settings, particularly in South Asia, whereas overweight/obesity was more common among international migrants, especially in high-income countries and in Europe and Central Asia. Moderator analyses identified female proportion, destination country or region, income level, and marital status as significant moderators for both outcomes.

**Conclusions:**

Migrant workers experience a substantial double burden of malnutrition, although the pattern varies across migration contexts. Undernutrition remains a concern in lower-income settings, whereas overweight and obesity are more common in high-income settings. Integrating nutrition-sensitive strategies into labour and migration health policies, including healthier food environments, routine nutritional screening, and culturally appropriate interventions, may help reduce health inequalities in this population.

**Registration:**

INPLASY: 202540030.

Urbanisation and globalisation have accelerated labour migration from developing countries to urban areas within national borders and to high-income countries, as individuals seek better employment and living conditions [1,2]. These global movements have resulted in growing populations of migrant workers, with over 169 million people engaged in international labour migration worldwide [1]. A migrant worker is broadly defined as an individual who relocates across geographic boundaries to engage in paid employment, either within a country (internal migration) or across international borders (international migration) [1,3]. However, definitions of migrant workers vary across disciplines and institutions, leading to inconsistencies in population scope and limiting comparability across studies. Given the heterogeneity of migrant populations, a structured classification framework was applied in this review to ensure conceptual consistency across studies. In this study, migrant workers were defined according to terminology from the International Organization for Migration and the International Labour Organization, encompassing individuals engaged in economically motivated labour migration, including internal rural – urban migrants, international migrant workers, seasonal migrant workers, and temporary contract migrant workers [1,4]. During migration, individuals frequently experience lifestyle and dietary disruptions due to unfamiliar food environments, limited financial resources, and demanding work conditions [5,6]. These stressors are compounded by legal uncertainty, social marginalisation, and limited access to healthcare in both transit and host countries [7], making migrant workers especially vulnerable to malnutrition.

Malnutrition, defined as a condition resulting from an imbalance, deficiency, or excess of nutrients that negatively affects body composition and health outcomes [8], has increasingly been recognised among migrant populations. Its determinants are multifactorial, including food insecurity, limited social integration, low dietary diversity, age-related vulnerabilities, and precarious living or working conditions [5,9,10]. This can result in undernutrition, micronutrient deficiencies, and growth-related issues such as stunting and wasting [7,8,11]. At the same time, migrants in high-income countries frequently experience overweight and obesity alongside ‘hidden hunger’ characterised by energy-dense but nutrient-poor diets, compounded by limited food autonomy and financial constraints [9,12].

These nutritional challenges not only compromise physical health but also reduce work productivity, diminish quality of life, and contribute to long-term healthcare burdens [13–16]. From a public health nutrition perspective, malnutrition among migrant workers represents a systemic failure in ensuring equitable access to nutrition and health services for vulnerable populations. Addressing this issue requires integration of nutrition into broader public health and migration policies through initiatives such as nutritional surveillance, workplace health programmes, culturally tailored dietary education, and food environment reform [1]. Without interventions, malnutrition among migrant workers will continue to fuel preventable diseases, widen health disparities, and increase economic burdens on health systems in both sending and receiving countries [5].

Previous studies reported various forms of malnutrition among migrants across regions. For instance, undernutrition remains a major concern in poor households with mothers who are migrant workers [7], while overweight and obesity are increasingly observed among migrants in high-income countries and among rural-to-urban migrants [17–19]. A systematic review by Ankomah *et al.* reported that overweight/obesity among migrants and refugees ranged from 11.1–42.0%, while undernutrition ranged from 0.3–17%, highlighting the double burden of malnutrition in these populations [9]. However, the existing evidence base remains fragmented and heterogeneous, with most studies focusing on specific subpopulations (*e.g.* refugees, women, or children) or limited geographic regions. Importantly, migrant workers, who constitute a large and distinct population with unique occupational, social, and environmental exposures, have not been comprehensively examined globally. Moreover, the influence of migration-related characteristics, such as type of migration (local *vs.* international) and length of stay, on nutritional outcomes remains poorly understood. Prolonged duration of stay may exacerbate nutritional vulnerability through persistent socioeconomic disadvantage or, alternatively, promote acculturation to obesogenic food environments, yet these dynamics have rarely been systematically assessed. In addition, variability in study design and methodological quality further complicates the interpretation of existing findings. Addressing these gaps, the present study conducted a global meta-analysis to estimate the pooled prevalence of underweight and overweight/obesity among local and international migrant workers and to examine key moderating factors influencing malnutrition.

## METHODS

### Search strategy and data sources

In this study, we followed guidelines established by the Meta-analysis of Observational Studies in Epidemiology and the updated PRRISMA guidelines [20,21] (Appendix S1 and S2 in the **Online Supplementary Document**). Our study was registered with the International Platform of Registered Systematic Review and Meta-analysis Protocols (202540030) [22]. Because we used aggregated, anonymised data from publicly available published sources, ethical approval was not required.

We conducted an updated comprehensive literature search on 4 March 2026 with the assistance of a university librarian across seven electronic databases – CENTRAL, Embase, PubMed, Scopus, Web of Science, Latin American and Caribbean Literature on Health Sciences, and African Index Medicus. We performed the search without restrictions on country, language, or publication year. To ensure comprehensive coverage, backward citation tracking was performed by screening the reference lists of included studies, and forward citation tracking was used to identify studies that cited them. Additionally, we searched Google Scholar and grey literature sources to identify potentially relevant studies that may not have been captured through database searches. Our search strategy involved a combination of Boolean operators and specialised terms tailored to each database. In CENTRAL, PubMed, Scopus, and Web of Science, we employed both free-text keywords and relevant MeSH terms to refine the search. For Embase, we used Emtree terms, the database’s controlled vocabulary, to enhance search specificity and ensure the inclusion of all criteria. This approach optimised the retrieval of relevant studies by adapting to each database’s unique indexing system.

The primary search concepts were structured around three main themes: epidemiological measures (*e.g.* prevalence and incidence); nutritional condition (*e.g.* malnutrition, thinness, and overweight); and mobile populations (*e.g.* migrants, nomads, and transients) (Appendices S3 and S4 in the **Online Supplementary Document**.

### Eligibility criteria

Regarding the inclusion criteria, the study population had to be comprised of migrant workers, defined according to terminology from the International Organization for Migration and the International Labour Organization, including internal rural-urban migrants, international migrant workers, seasonal migrant workers, and temporary contract migrant workers. These groups were defined as individuals engaged in economically motivated labour migration within or across national borders (Appendix S5 in the **Online Supplementary Document**) [1,4]. Malnutrition had to be assessed in accordance with World Health Organization (WHO) definitions [8], with being underweight defined as a body-mass index (BMI)<18.5 kg/m^2^ and overweight/obesity as a BMI>25 kg/m^2^. Studies had to report quantitative prevalence data on malnutrition among migrant workers and use observational designs, including cross-sectional and cohort studies, which are well-suited for estimating prevalence and identifying trends. Intervention studies (*e.g.* randomised controlled trials) were also included if baseline nutritional data were available prior to the intervention. We excluded studies that did not focus on malnutrition or migrant workers, involved populations outside the scope of this review, used qualitative or other designs unsuitable for prevalence estimation, or lacked sufficient data for analysis.

### Screening, data extraction, quality assessment and certainty of evidence assessment

Two researchers (HAN and AAP) independently reviewed the data using EndNote, version 21 (Clarivate Analytics, Philadelphia, Pennsylvania, USA). Initially, duplicates were removed, and titles and abstracts of the remaining were screened. This was followed by a full-text review to assess whether the studies met the inclusion criteria. Disagreements were resolved through discussion with a third researcher (SCY.) if necessary. To ensure accuracy and thoroughness, the reviewers contacted the authors of published articles for additional data or clarification. Once eligible full texts were obtained, data extraction was performed by two researchers (HA and HS) who had previously agreed on the information to be extracted. The extracted data included: study characteristics, such as authors, publication year, country, and study design; demographic data and risk factors, such as sample size, gender, age, comorbidities, behavioural risk factors, educational level, migrant setting, and migrant type; and screening tools and malnutrition (underweight and overweight/obesity) prevalence. When a study reported prevalence estimates for multiple independent populations (*e.g.* different countries, migrant types, or survey years), we treated these estimates as separate data points in the meta-analysis because they represented distinct population samples [23,24].

We appraised the study quality using the Joanna Briggs Institute tool for prevalence studies [23,25], which comprises nine domains that evaluate sampling, measurement, and analysis. Each domain was scored as ‘yes,’ ‘no,’ ‘unclear,’ or ‘not applicable,’ and overall quality was categorised as high (>70%), moderate (50–70%), or low (<50%) based on the number of ‘yes’ scores [26].

We assessed the certainty of evidence using the GRADE approach [27], considering risk of bias, inconsistency, indirectness, imprecision, and publication bias. Ratings were assigned as high, moderate, low, or very low. Downgrading occurred due to serious concerns, such as high heterogeneity (*I^2 ^*> 95) or publication bias (*e.g.* funnel plot asymmetry, Peters’ test *P* < 0.05) [28]. All disagreements during quality or certainty assessments were resolved by consensus or third-party adjudication.

### Statistical analysis

We calculated pooled prevalence estimates of underweight and overweight/obesity using a random-effects meta-analysis, accounting for between-study heterogeneity. Prevalence was defined as the proportion of malnutrition cases among migrant workers in each study and was expressed as a percentage, with corresponding 95% confidence intervals (CIs) and 95% prediction intervals. We assessed statistical heterogeneity using Cochran’s Q test and the *I^2^* statistic. Heterogeneity was considered substantial when *I^2^* exceeded 30%, and the Q test was statistically significant (*P* < 0.10) [24]. Given the presence of substantial heterogeneity, moderator analyses were conducted to explore potential sources of variability across studies. We performed a random-effects meta-regression using study-level covariates, including mean age (in years), mean duration of stay (in years), and percentage of female participants. Meta-regression coefficients represent absolute percentage-point changes in prevalence associated with a one-unit increase in the moderator variable (*e.g.* years for mean age or percentage points for female proportion). We modelled prevalence estimates using generalised linear mixed models with random effects. Continuous moderators were entered in their original measurement units to maintain interpretability of the regression coefficients [23,24]. In addition, we conducted subgroup analyses according to study characteristics (study design, publication year, study setting, sample size category, study quality, destination region, and country income level) and participant characteristics (marital status, educational level, migration setting, migration scope, and migrant type). The choice of these moderators was guided by the Social Determinants of Health (SDOH) framework [29]. In this framework, destination region and country income level represent structural determinants that capture broader socioeconomic and policy contexts shaping migrant health, whereas individual characteristics such as education, age, sex, and marital status reflect socioeconomic position. Study setting and migration context, including urban or rural environments, were treated as proxies for intermediary determinants related to living conditions, access to services, and health system context. Using the SDOH framework provided a theory-driven basis for subgroup stratification and strengthened the interpretability of subgroup findings. A *P*-value <0.05 was considered statistically significant.

We conducted sensitivity analyses to evaluate the robustness of the pooled estimates. Specifically, we applied a leave-one-out approach, sequentially excluding the study with the smallest and the largest statistical weight to assess the influence of individual studies on the overall results. Further sensitivity analyses were also conducted according to study design and migrant worker type to examine the consistency of the findings across methodological and population subgroups. We assessed publication bias through visual inspection of funnel plots and Peters’ regression test, with *P* ≥ 0.10 indicating no statistically significant evidence of small-study effects or publication bias [30]. When asymmetry was observed, or the Peters’ test indicated potential bias, the trim-and-fill method was applied to estimate the number of potentially missing studies and to evaluate the impact of such bias on the pooled estimates [31]. The adjusted pooled prevalence obtained after imputing missing data from potentially missing studies was compared with the original estimate to assess whether publication bias materially influenced the results. Peters’ test suggested possible small-study effects; however, this finding should be interpreted cautiously because funnel plot asymmetry and regression-based tests in prevalence meta-analyses may be influenced by substantial heterogeneity and sample size variation. We performed all analyses using Comprehensive Meta-Analysis, version 3.0 (Biostat, Englewood, New Jersey, USA) [30].

## RESULTS

### Study selection and characteristics

**Figure 1:**
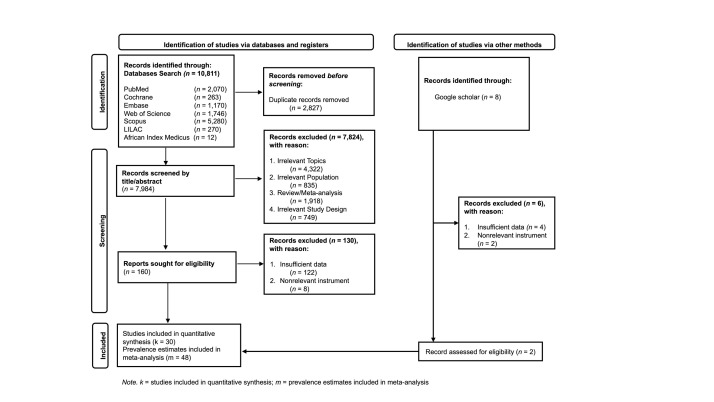
PRISMA flow diagram of article selection process.

We initially identified 10 811 relevant studies from electronic databases. After removing 2827 duplicates, 7984 studies remained. Of these screened by title and abstract, 7824 records were excluded due to irrelevant topics, populations, or study designs. Subsequently, we assessed 160 full-text studies for eligibility; 130 were excluded due to insufficient data or inappropriate measurement tools. As a result, 28 eligible studies were included in the analysis. To ensure the comprehensiveness of the search, we performed an additional manual search on Google Scholar and conducted citation tracking, which yielded three additional eligible studies. Therefore, 30 studies were included in the meta-analysis. From these studies, 22 data points on underweight and 48 data points on overweight/obesity were extracted. Specifically, one study [32] provided malnutrition data by the destination country, one study [33] provided data by migrant type, two studies [34,35] by year of data collection, two studies [6,36] by host country, and two studies [37,38] by category of migrant worker (**Figure 1****,** **Table 1**).

**Table 1:** Characteristics of included studies

Study	Country	Study design	Demographic data and risk factors				Tools, underweight prevalence, overweight/obesity prevalence
			**Sample size and gender**	**Age in years, x̄ (SD); comorbidities; behaviour risk**	Education*	**Migrant type and setting**	
Abasilim *et al.*, 2024 [10]	USA	Cross-sectional	n = 102; female n = 101 (99%), male n = 1 (1%)	NA	<secondary level n = 38 (37.3%); ≥secondary level n = 64 (62.7%)	Farmworkers; international	Tools: BMI (WHO); Underweight: NA; Overweight/obesity n = 77 (75.5%)
Afrifa-Anane *et al.*, 2020 [32]	Netherlands	Cross-sectional	n = 983; female n = 612 (62.3%), male n = 371 (37.7%)	Age: 46.9 (8.4); Comorbidities: NA; Smoking n = 109 (11.1%)	<secondary level n = 763 (77.6%); ≥secondary level n = 220 (22.4%)	International	Tools: BMI (WHO); Underweight: NA; Overweight/obesity n = 792 (80.6%)
Afrifa-Anane *et al.*, 2020 [32]	Germany	Cross-sectional	n = 523; female n = 234 (44.7%), male n = 289 (55.3%)	Age: 45.2 (10.3); Comorbidities: NA; Smoking n = 108 (20.6%)	<secondary level n = 318 (60.8%); ≥secondary level n = 205 (39.2%)	International	Tools: BMI (WHO); Underweight: NA; Overweight/obesity n = 369 (70.6%)
Afrifa-Anane *et al.*, 2020 [32]	UK	Cross-sectional	n = 891; female n = 553 (62.1%), male n = 338 (37.9%)	Age: 47.1 (10.6); Comorbidities: NA; Smoking n = 47 (5.3%)	<secondary level n = 399 (44.8%); ≥secondary level n = 492 (55.2%)	International	Tools: BMI (WHO); Underweight: NA; Overweight/obesity n = 742 (83.3%)
Afrifa-Anane *et al.*, 2020 [32]	Ghana	Cross-sectional	n = 487; female n = 100 (20.5%), male n = 387 (79.5%)	Age: 46.3 (11.7); Comorbidities: NA; Smoking n = 77 (15.8%)	<secondary level n = 354 (72.7%); ≥secondary level n = 133 (27.3%)	Local	Tools: BMI (WHO); Underweight: NA; Overweight/obesity n = 313 (64.3%)
Afrifa-Anane *et al.*, 2020 [32]	Ghana	Cross-sectional	n = 972; female n = 594 (61.1%), male n = 378 (38.9%)	Age: 46.4 (12.6); Comorbidities: NA; Smoking n = 87 (8.9%)	<secondary level n = 864 (88.9%); ≥secondary level n = 108 (11.1%)	Local	Tools: BMI (WHO); Underweight: NA; Overweight/obesity n = 231 (23.8%)
Alhadlaq *et al.*, 2023 [34]	Kuwait	Cross-sectional	n = 3478 (n for females and males NA)	Age: 40.8 (12.4); Comorbidities: NA; Smoking n = 832 (23.9%)	NA	Constructors, refinery, and agricultural workers; Temporary contract; Local and international	Tools: BMI (WHO); Underweight n = 30 (0.9%); Overweight/obesity n = 2449 (70.4%)
Alhadlaq *et al.*, 2023 [34]	Kuwait	Cross-sectional	n = 3807 (n for females and males NA)	Age: NA; Comorbidities: NA; Smoking n = 669 (17.6%)	NA	Constructors, refinery, and agricultural workers; Temporary contract; Local and international	Tools: BMI (WHO); Underweight n = 29 (0.8%); Overweight/obesity n = 1908 (50.1%)
Ali *et al.*, 2022 [39]	Kuwait	Cross-sectional	n = 3474 (n for females and males NA)	Age: NA; Comorbidities: hypertension n = 412 (11.8%), diabetes n = 350 (10.1%); Smoking n = 831 (23.9%)	NA	Industrial workers; Local and international	Tools: BMI (WHO); Underweight n = 77 (2.2%); Overweight/obesity n = 2449 (70.5%)
Arslan *et al.*, 2023 [40]	Turkey	Cross-sectional	n = 450; female n = 450 (100.0%)	Age: 35.3 (11.4); Comorbidities: NA; Behaviour risks: NA	<secondary level n = 108 (24.0%); ≥secondary level n = 342 (76.0%)	International	Tools: BMI (WHO); Underweight n = 33 (7.3%); Overweight/obesity n = 159 (35.3%)
Aung *et al.*, 2019 [14]	Thailand	Cross-sectional	n = 414; female n = 183 (44.2%), male n = 231 (55.8%)	Age: 29.4 (9.0); Comorbidities: hypertension n = 112 (27.0%), diabetes n = 4 (0.9%); Smoking n = 109 (26.3%), alcohol n = 169 (40.8%), exercise n = 63 (15.2%), depression n = 54 (13.0%)	<secondary level n = 316 (76.3%); ≥secondary level n = 98 (23.7%)	Domestic, construction, agriculture, factory workers; Temporary contract; International	Tools: BMI (APG); Underweight n = 15 (3.6%); Overweight/obesity n = 166 (40.0%)
Aung *et al.*, 2024 [41]	Thailand	Cross-sectional	n = 360; female n = 168 (46.7%), male n = 192 (53.3%)	Age: 29.7 (9.2); Comorbidities: hypertension n = 102 (27.0%), depression n = 52 (14.4%); Smoking n = 90 (25.0%), alcohol n = 138 (38.3%), exercise n = 52 (14.4%)	<secondary level n = 191 (53.1%); ≥secondary level n = 169 (46.9%)	Domestic, construction, agriculture, factory workers; Temporary contract; International	Tools: BMI (WHO); Underweight: NA; Overweight/obesity n = 81 (22.5%)
Bhandari *et al.*, 2023 [42]	South Korea	Cross-sectional	n = 141; female n = 17 (12.1%), male n = 122 (86.5%)	Age: 31.3 (5.6); Comorbidities: hypertension n = 94 (66.6%), dyslipidaemia n = 91 (63.2%), diabetes n = 1 (0.7%); Smoking n = 21 (14.9%), alcohol n = 67 (47.5%), exercise n = 58 (41.1%)	<secondary level n = 21 (14.9%); ≥secondary level n = 120 (85.1%)	Agriculture, construction, manufacturing workers; Temporary contract; International	Tools: BMI (WHO); Underweight n = 6 (4.3%); Overweight/obesity n = 45 (31.9%)
Bi *et al.*, 2015 [33]	China	Cross-sectional	n = 48 704; female n = 21 816 (44.8%), male n = 26 888 (55.2%)	Age: 35.7 (10.6); Comorbidities: hypertension n = 7939 (16.3%), dyslipidaemia n = 16 803 (34.5%), diabetes n = 2484 (5.1%); Smoking n = 15 829 (32.5%), exercise n = 13 637 (27.9%)	<secondary level n = 25 521 (52.4%); ≥secondary level n = 23 183 (47.6%)	Manufacturing, wholesale and retail trade, accommodation and catering, services, construction workers; Local	Tools: BMI (WHO); Underweight: NA; Overweight/obesity n = 15341 (31.5%)
Bi *et al.*, 2015 [33]	China	Cross-sectional	n = 8404; female n = 3765 (44.8%), male n = 4639 (55.2%)	Age: 31.8 (10.6); Comorbidities: NA; Smoking n = 2672 (31.8%)	<secondary level n = 4042 (48.1%); ≥secondary level n = 4362 (51.9%)	Manufacturing; Local	Tools: BMI (WHO); Underweight: NA; Overweight/obesity n = 2395 (28.5%)
Bi *et al.*, 2015 [33]	China	Cross-sectional	n = 7826; female n = 3803 (48.6%), male n = 4023 (51.4%)	Age: 34.3 (10.6); Comorbidities: NA; Smoking n = 2684 (34.3%)	<secondary level n = 4476 (57.2%); ≥secondary level n = 3349 (42.8%)	Wholesale and retail trade; Local	Tools: BMI (WHO); Underweight: NA; Overweight/obesity n = 2630 (33.6%)
Bi *et al.*, 2015 [33]	China	Cross-sectional	n = 8332; female n = 4149 (49.8%), male n = 4183 (50.2%)	Age: 32.7 (10.6); Comorbidities: NA; Smoking n = 2724 (32.7%)	<secondary level n = 5166 (62.0%); ≥secondary level n = 3166 (38.0%)	Accommodation and catering; Local	Tools: BMI (WHO); Underweight: NA; Overweight/obesity n = 2500 (30.0%)
Bi *et al.*, 2015 [33]	China	Cross-sectional	n = 8225; female n = 4047 (49.2%), male n = 4178 (50.8%)	Age: 32.6 (10.6); Comorbidities: NA; Smoking n = 2681 (32.6%)	<secondary level n = 3331 (40.5%); ≥secondary level n = 4894 (59.5%)	Services; Local	Tools: BMI (WHO); Underweight: NA; Overweight/obesity n = 2517 (30.6%)
Bi *et al.*, 2015 [33]	China	Cross-sectional	n = 8103; female n = 1110 (13.7%), male n = 6993 (86.3%)	Age: 36.7 (10.6); Comorbidities: NA; Smoking n = 2974 (36.7%)	<secondary level n = 4918 (60.7%); ≥secondary level n = 3184 (39.3%)	Construction workers; Local	Tools: BMI (WHO); Underweight: NA; Overweight/obesity n = 2998 (37.0%)
Bi *et al.*, 2015 [33]	China	Cross-sectional	n = 7814; female n = 2891 (37.0%), male n = 4923 (63.0%)	Age: 33.7 (10.6); Comorbidities: NA; Smoking n = 2633 (33.7%)	<secondary level n = 4032 (51.6%); ≥secondary level n = 3782 (48.4%)	Others; Local	Tools: BMI (WHO); Underweight: NA; Overweight/obesity n = 2641 (33.8%)
Carioca *et al.*, 2017 [36]	Brazil	Cross-sectional	n = 349; female n = 219 (62.8%), male n = 130 (37.2%)	Age: 59.3 (17.7); Comorbidities: hypertension n = 148 (42.4), diabetes n = 42 (12.0%); Smoking n = 59 (16.9%), alcohol n = 163 (46.7%), exercise n = 60 (17.2%)	<secondary level n = 240 (68.8%);≥secondary level n = 109 (31.9%)	International	Tools: BMI (WHO); Underweight n = 49 (14%); Overweight/obesity n = 135 (38.7%)
Carioca *et al.*, 2017 [36]	Brazil	Cross-sectional	n = 296; female n = 183 (61.8%), male n = 118 (38.2%)	Age: 52.2 (18.6); Comorbidities: hypertension n = 121 (40.8), diabetes n = 36 (12.2%); Smoking n = 48 (16.2%), alcohol n = 108 (36.5%), exercise n = 43 (14.5%)	<secondary level n = 249 (84.1%); ≥secondary level n = 47 (15.9%)	International	Tools: BMI (WHO); Underweight n = 25 (8.4%); Overweight/obesity n = 97 (32.8%)
Carioca *et al.*, 2021 [6]	Brazil	Cross-sectional	n = 145; female n = 76 (52.4%), male n = 69 (47.6%)	Age: 48.7 (18.2); Comorbidities: NA; Smoking n = 31 (21.8%), alcohol n = 70 (48.3%), exercise n = 113 (77.9%)	<secondary level n = 69 (47.6%); ≥secondary level n = 76 (52.4%)	International	Tools: BMI (WHO); Underweight n = 10 (6.9%); Overweight/obesity n = 77 (53.1%)
Carioca *et al.*, 2021 [6]	Brazil	Cross-sectional	n = 148; female n = 78 (52.7%), male n = 70 (47.3%)	Age: 42.4 (16.4); Comorbidities: NA; Smoking n = 24 (16.2%), alcohol = 62 (41.9%), exercise n = 122 (82.4%)	<secondary level n = 97 (65.5%); ≥secondary level n = 51 (34.5%)	International	Tools: BMI (WHO); Underweight n = 3 (2.0%); Overweight/obesity n = 67 (45.3%)
Castaneda *et al.*, 2015 [37]	USA	Cross-sectional	n = 282; female n = 163 (57.8%), male n = 118 (41.8%)	Age: 34.7 (11.3); Comorbidities: hypertension n = 30 (10.6%); dyslipidaemia n = 20 (7.1%), diabetes n = 20 (7.1%) Smoking n = 47 (16.6%)	<secondary level n = 219 (77.7%); ≥secondary level n = 63 (22.3%)	Agricultural workers; Temporary contract; Local and international	Tools: BMI (WHO); Underweight: NA; Overweight/obesity n = 92 (32.6%)
Castaneda *et al.*, 2015 [37]	USA	Cross-sectional	n = 175; female n = 95 (54.3%), male n = 80 (45.7%)	Age: 35.0 (11.6); Comorbidities: hypertension n = 22 (12.6%), dyslipidaemia n = 14 (8.0%), diabetes n = 12 (6.8%); Smoking n = 28 (16.0%)	<secondary level n = 135 (77.1%); ≥secondary level n = 40 (22.9%)	Agricultural workers; Seasonal; Local and international	Tools: BMI (WHO); Underweight: NA; Overweight/obesity n = 61 (34.9%)
Castaneda *et al.*, 2015 [37]	USA	Cross-sectional	n = 107; female n = 68 (63.6%), male n = 39 (36.4%)	Age: 34.2 (10.6); Comorbidities: hypertension n = 8 (7.5%), dyslipidaemia n = 6 (5.6%), diabetes n = 12 (11.2%); Smoking n = 19 (17.7%)	<secondary level n = 84 (78.5%); ≥secondary level n = 23 (21.5%)	Agricultural workers; Local and international	Tools: BMI (WHO); Underweight: NA; Overweight/obesity n = 31 (29.0%)
Castaneda *et al.*, 2019 [15]	Mexico	Cross-sectional	n = 146; female n = 83 (56.8%), male n = 63 (43.2%)	Age: 43.5 (14.2); Comorbidities: NA; Behaviour risks: NA	<secondary level n = 141 (96.6%); ≥secondary level n = 5 (3.4%)	Agricultural workers; Temporary contract; Local	Tools: BMI (WHO); Underweight: NA; Overweight/obesity n = 109 (74.7%)
Charoensook *et al.*, 2018 [43]	Thailand	Cross-sectional	n = 467; female n = 207 (44.3%), male n = 260 (55.7%)	Age: NA; Comorbidities: hypertension n = 7 (1.5%), diabetes n = 2 (0.4%), depression n = 355 (76.0%); Behaviour risks: NA	NA	Temporary contract; International	Tools: BMI (WHO); Underweight: 38 (8.1%); Overweight/obesity n = 201 (43.0%)
Dah Poe *et al.*, 2022 [44]	Thailand	Cross-sectional	n = 402; female n = 233 (58.0%), male n = 169 (42.0%)	Age: 38.9 (10.4); Comorbidities: hypertension n 0 87 (21.6), diabetes n = 54 (13.4%), depression n = 97 (24.1%); Smoking n = 37 (9.2%), alcohol n = 93 (23.1%), exercise n = 203 (50.5%)	<secondary level n = 139 (34.6%); ≥secondary level n = 263 (65.4%)	Labour and agriculture workers; International	Tools: BMI (WHO); Underweight n = 29 (7.2%); Overweight/obesity n = 156 (38.8%)
Emiral *et al.*, 2021 [45]	Turkey	Cross-sectional	n = 455; female n = 306 (67.3%), male n = 149 (32.7%)	35.0 (13.8); Comorbidities: hypertension n = 79 (17.4%), dyslipidaemia n = 31 (6.8%), diabetes n = 17 (3.7%); Smoking n = 152 (33.4%)	NA	Agricultural workers; Temporary contract; Local	Tools: BMI (WHO); Underweight: NA; Overweight/obesity n = 177 (38.9%)
Hall *et al.*, 2021 [46]	Macao	Cross-sectional	n = 1388; female n = 1388 (100.0%)	Age: 41.3 (9.6); Comorbidities: NA; Behaviour risks: NA	<secondary level n = 25 (1.8%); ≥secondary level n = 1363 (98.2%)	Domestic workers; Temporary contract; International	Tools: BMI (WPRO); Underweight n = 23 (1.7%); Overweight/obesity n = 872 (62.8%)
Huang *et al.*, 2020 [47]	Macao	Cross-sectional	n = 1388; female n = 1388 (100.0%)	NA	NA	Domestic workers; Temporary contract; International	Tools: BMI (WHO); Underweight n = 23 (1.7%); Overweight/obesity n = 872 (62.8%)
Huang *et al.*, 2020 [47]	Macao	Cross-sectional	n = 369; female n = 369 (100.0%)	NA	NA	Domestic workers; Temporary contract; International	Tools: BMI (WHO); Underweight n = 14 (3.8%); Overweight/obesity n = 201 (54.5%)
Jorgensen *et al.*, 2011 [48]	Denmark	Cross-sectional	n = 161 (n for females and males NA)	Age: 42.8 (8.2); Comorbidities: hypertension n = 51 (31.7%); Smoking n = 41 (25.7%), exercise n = 24 (14.9%)	NA	Domestic workers; Temporary contract; International	Tools: BMI (WHO); Underweight: NA; Overweight/obesity n = 115 (71.4%)
Joy *et al.*, 2017 [49]	India	Cross-sectional	n = 6755; female n = 2895 (42.7%), male n = 3880 (57.3%)	Age: 41 (10.0); Comorbidities: hypertension n = 1685 (24.9%), diabetes n = 682 (10.1); Behaviour risks: NA	<secondary level n = 1678 (24.8%); ≥secondary level n = 5097 (75.2%)	Manufacturing workers; Local	Tools: BMI (WHO); Underweight n = 796 (11.4%); Overweight/obesity n = 2563 (37.8%)
Khairizka Citra *et al.*, [50]	Taiwan	Cross-sectional	n = 235; female n = 235 (100.0%)	Age: 32.5 (6.9); Comorbidities: NA; Behaviour risks: NA	<secondary level n = 29 (12.3%); ≥secondary level n = 206 (87.7%)	Domestic workers; Temporary contract; International	Tools: BMI (WHO); Underweight: NA; Overweight/obesity n = 120 (51.1%)
Kowalski *et al.*, 2019 [51]	USA	Cross-sectional	n = 150; female n = 96 (64.0%), male n = 54 (36.0%)	Age: 39.1 (14.2); Comorbidities: hypertension n = 34 (22.7%), dyslipidaemia n = 30 (20%), diabetes n = 58 (38.7%); Behaviour risks: NA	NA	Farmworkers; International	Tools: BMI (WHO); Underweight: NA; Overweight/obesity n = 90 (60.0%)
Kuhn *et al.*, 2020 [38]	Bangladesh	Cross-sectional	n = 1260 (n for females and males NA)	Age: 30.8 (8.2); Comorbidities: hypertension n = 159 (12.6%); Smoking n = 475 (37.7%)	NA	Local	Tools: BMI (APG); Underweight n = 141 (11.2%); Overweight/obesity n = 475 (37.7%)
Kuhn *et al.*, 2020 [38]	Bangladesh	Cross-sectional	n = 790 (n for females and males NA)	Age: 32.9 (6.7); Comorbidities: hypertension n = 103 (13.0%); Smoking n = 258 (32.7%)	NA	International	Tools: BMI (APG); Underweight n = 17 (2.2%); Overweight/obesity n = 408 (51.7%)
Leong *et al.*, 2006 [52]	Malaysia	Cross-sectional	n = 76; female n = 76 (100.0%)	Age: NA; Comorbidities: hypertension n = 76 (100.0%); Behaviour risks: NA	NA	Domestic workers; Temporary contract; International	Tools: BMI (WHO); Underweight n = 6 (7.9%); Overweight/obesity n = 25 (32.9%)
Lopez-Cevallos *et al.*, 2019 [53]	USA	Cross-sectional	n = 3382; female n = 673 (19.9%), male n = 2709 (80.1%)	Age: NA; Comorbidities: hypertension n = 337 (9.9%), dyslipidaemia n = 718 (21.2%), diabetes n = 167 (4.9%); Behaviour risks: NA	NA	Farmworkers; Temporary contract; Local	Tools: BMI (WHO); Underweight: NA; Overweight/obesity n = 691 (20.4%)
Mei *et al.*, 2020 [54]	Malaysia	Cross-sectional	n = 125; female n = 50 (40.0%), male n = 75 (60.0%)	Age: 32.5 (7.9); Comorbidities: NA; Behaviour risks: NA	NA	Construction, cleaning, domestic, security guard, factory, foodservice workers; Temporary contract; International	Tools: BMI (WHO); Underweight n = 4 (3.2%); Overweight/obesity n = 36 (28.8%)
Peng *et al.*, 2022 [55]	China	Cross-sectional	n = 232; female n = 232 (100.0%)	Age: 34.4 (6.4); Comorbidities: hypertension n = 74 (31.9%); Smoking n = 3 (1.3%), exercise n = 43 (18.5%)	<secondary level n = 26 (11.2%); ≥secondary level n = 206 (88.8%)	Manufacturing workers; Local	Tools: BMI (APG); Underweight: NA; Overweight/obesity n = 63 (27.2%)
Sanskriti *et al.*, 2024 [56]	India	Cross-sectional	n = 96 (n for females and males NA)	Age: NA; Comorbidities: hypertension n = 76 (79.1%); Behaviour risks: NA	NA	Local	Tools: BMI (APG); Underweight n = 20 (20.8%); Overweight/obesity n = 17 (17.7%)
Shan *et al.*, 2011 [35]	China	Cohort	n = 707; female n = 304 (42.9%), male n = 403 (57.1%)	Age: 37.6 (11.3); Comorbidities: NA; Smoking n = 315 (44.6%), alcohol n = 316 (44.7%)	<secondary level n = 293 (21.0%); ≥secondary level n = 1100 (79.0%)	Farmworkers; Local	Tools: BMI (WHO); Underweight: NA; Overweight/obesity n = 129 (34.2%)
Shan *et al.*, 2011 [35]	China	Cohort	n = 707; female n = 568 (40.8%), male n = 825 (59.2%)	Age: 39.4 (11.8); Comorbidities: NA; Smoking n = 549 (39.4%), alcohol n = 715 (51.3%)	<secondary level n = 293 (21.0%); ≥secondary level n = 1100 (79.0%)	Farmworkers; Local	Tools: BMI (WHO); Underweight: NA; Overweight/obesity n = 477 (34.2%)
Sornlorm *et al.*, 2024 [57]	Thailand	Cross-sectional	n = 406; female n = 175 (43.1%), male n = 231 (56.9%)	Age: 32.3 (9.3); Comorbidities: depression: 213 (52.7%); Smoking n = 228 (56.2%), alcohol n = 214 (52.7%), exercise n = 339 (83.5%)	<secondary level n = 106 (26.1%); ≥secondary level n = 300 (73.9%)	Construction and manufacturing workers; Temporary contract; International	Tools: BMI (WHO); Underweight n = 47 (1.6%); Overweight/obesity n = 162 (39.9%)

APG – Asia Pacific guidelines, BMI – body mass index, NA – not available, WPRO – World Health Organization Regional Office for the Western Pacific

*Education level:<secondary level, lower than high school; ≥secondary level, graduate and higher than high school.

In the analysis of 30 studies, 135 404 migrant workers were examined, with a mean age of 32.3 years (standard deviation = 11.1). Most of these studies were cross-sectional (97.9%) [6,10,14,15,32–34,36-39,41–45,48–51,53–57] and published between 2016 and 2020 (39.1%) [11,14,15,32,36,38,43,47,49,50,53]. East Asia and the Pacific (45.8%) were the primary destination chosen by the majority of migrant workers [11,14,33,35,41–44,47,50,55,57], and they were headed to high-income countries (45.8%) [10,32,34,35,37,39,42,47,48,50,51,53]. Research on malnutrition was primarily conducted in community settings, and malnutrition was assessed using the WHO BMI classification (85.4%) (Appendix S6 in the **Online Supplementary Document**).

### Quality assessment and evidence

We identified 76.7% studies rated as high quality, 16.7% as moderate quality, and 6.7% as low quality. The overall methodological quality was acceptable, with common limitations related to sample size justification, sampling representativeness, and response rate reporting (Appendix S7 in the **Online Supplementary Document**).

We rated the certainty of evidence for both underweight and overweight/obesity outcomes as low using the GRADE approach adapted for prevalence studies [28]. This rating primarily reflected very high heterogeneity (*I^2^*>97) and evidence of publication bias, while no serious concerns were identified for indirectness or imprecision. Overall, these findings indicate substantial variability in prevalence estimates across studies and highlight the need for more standardised and methodologically robust research on migrant nutrition (Appendix S8 in the **Online Supplementary Document**).

### Results of the meta-analysis and sensitivity analysis

#### Pooled prevalence of underweight

**Figure 2:**
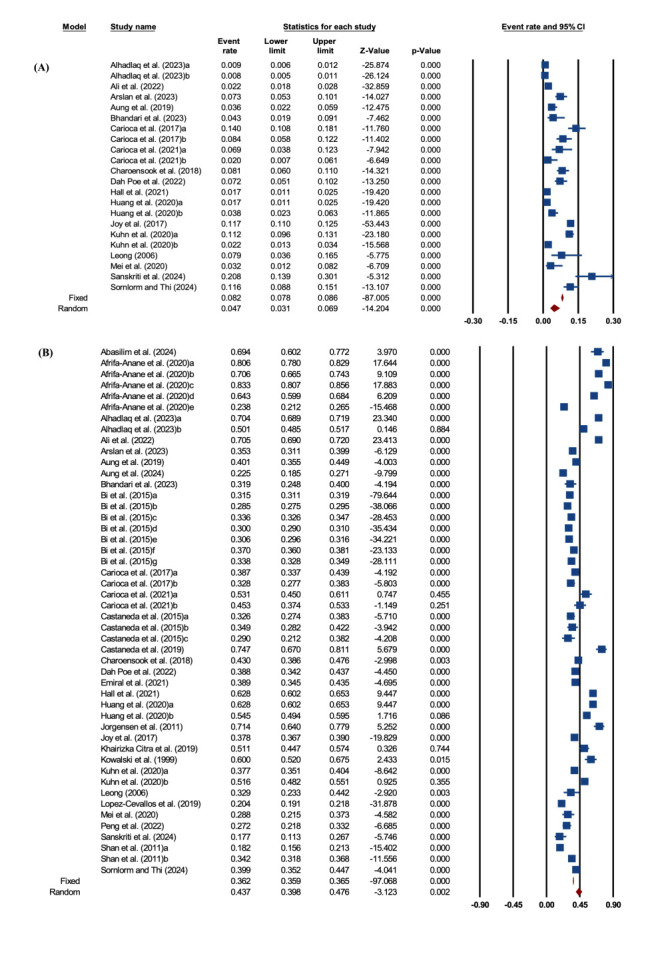
Pooled prevalence among migrant workers. **Panel A.** Underweight. **Panel B.** Overweight/obesity among migrant workers.

**Figure 3:**
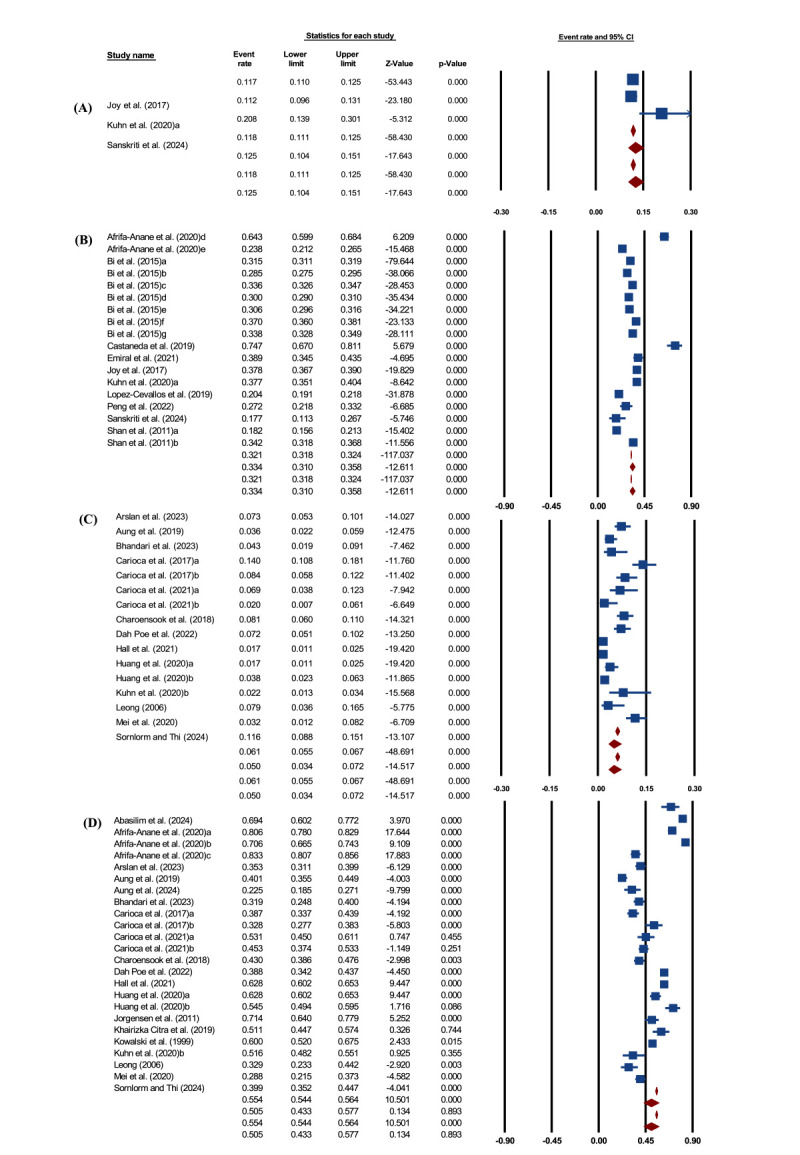
Pooled prevalence. **Panel A.** Underweight among local migrant workers. **Panel B.** Overweight/obesity among local migrant workers. **Panel C.** Underweight among international migrant workers. **Panel D.** Overweight/obesity among international migrant workers.

The pooled prevalence of underweight among migrant workers was 4.7% (95% CI = 3.1, 6.9) (n = 26 244) [6,11,14,34,36,38–40,42–44,46,47,49,52,56,57]; the corresponding prediction interval value ranged between 0.7 % and 33.6 % for potential future studies. Heterogeneity was considerable (Q = 835 442.19, *P* < 0.001, *I^2^* = 97.5), and a random-effects model was used (**Figure 2**, Panel A). Subgroup analysis revealed a higher prevalence of underweight among local migrants (12.5%; 95% CI = 10.4, 15.1) than among international migrant workers (5.0%; 95% CI = 3.4, 7.2) (**Figure 3**, Panels A and B). Visual inspection of the funnel plot and Peters’ regression (*P* = 0.02) suggested asymmetry and possible small-study effects. However, Duval and Tweedie’s trim-and-fill method imputed zero missing studies, so the adjusted pooled estimate was essentially unchanged (no meaningful adjustment) (Appendix S9 and S10 in the **Online Supplementary Document**).

#### Pooled prevalence of overweight/obesity

The pooled prevalence of overweight/obesity among migrant workers was 43.7% (95% CI = 39.8, 47.6) (n = 135 404) [6,10,11,14,15,32–53,55,56]. The corresponding prediction interval value ranged between 20.6% and 69.8% for potential future studies. Heterogeneity was high (Q = 75 995.83, *P* < 0.001, *I^2^* = 99.4), and a random-effects model was therefore applied (**Figure 2**, Panel B). By migrant type, international migrants had the highest prevalence (50.5%; 95% CI = 43.3, 57.7), followed by local migrants (33.4%; 95% CI = 31.0, 35.8 (**Figure 3**, Panels C and D). Regarding publication bias, the funnel plot demonstrated asymmetry. Publication bias was suggested by funnel plot asymmetry and supported by Peters’ regression test (*P* = 0.01). Trim-and-fill analysis indicated potential small-study effects, with five studies estimated to be missing on the right side of the funnel plot. After imputing these studies using a random-effects model, the pooled prevalence increased slightly from 43.7% (95% CI = 39.8, 47.6) to 46.7% (95% CI = 42.5, 51.0) (Appendix S1 and S12 in the **Online Supplementary Document**). The small difference between the observed and adjusted estimates indicates that the pooled prevalence remained stable, suggesting that potential publication bias had limited influence on the overall findings.

#### Sensitivity analysis

Sensitivity analyses supported the robustness of the pooled estimates. For underweight, excluding the study with the smallest statistical weight [34] yielded a revised prevalence of 5.1% (95% CI = 3.5, 7.4), whereas excluding the study with the largest statistical weight [56] resulted in a prevalence of 4.3% (95% CI = 2.9, 6.5). Additional leave-one-out analyses produced similar estimates. When we examined studies by migrant worker categories, the pooled prevalence across all categories was 4.8% (95% CI = 3.2, 7.3). Excluding a study representing local migrants [38,49,56] yielded a comparable estimate of 5.1% (95% CI = 3.7, 7.0), whereas exclusion of a highly weighted study representing international migrant workers [6,14,36,38,40,42–44,46,47,52,54,57] resulted in a prevalence of 4.7% (95% CI = 3.1, 7.1).

For overweight/obesity, excluding the study with the smallest statistical weight [56] yielded an estimated prevalence of 44.2% (95% CI = 40.3, 48.2), whereas excluding the study with the largest statistical weight [32] resulted in a prevalence of 42.7% (95% CI = 38.9, 46.5). When examined across migrant worker categories, the pooled prevalence was 44.1% (95% CI = 40.5, 47.8). Excluding a study of short-term migrants [37] produced a comparable estimate of 44.2% (95% CI = 40.6, 47.9), while excluding a highly weighted study of international migrant workers [6,10,14,32,36,38,40–44,46–48,50–52,54,57] yielded a slightly lower prevalence of 40.6% (95% CI = 36.6, 44.8). Additional sensitivity analyses that excluded the cohort study [52] yielded a similar estimate of 43.9% (95% CI = 39.9, 47.9). Overall, these analyses indicate that the pooled estimates were stable and not materially influenced by individual studies.

### Result of the moderator analyses

#### Underweight among migrant workers

Meta-regression and subgroup analyses were performed to identify potential sources of heterogeneity among the included studies. The meta-regression analysis revealed female proportion (6.6%; 95% CI = 5.4, 7.7) was a significant moderator of the pooled prevalence of being underweight among migrant workers, even though the mean age (2.2%; 95% CI = –3.9, 8.3) and length of stay (3.3%; 95% CI = –1.3, 8.3) non-significantly moderated it (**Table 2**).

**Table 2:** Result of moderator analysis

Variables	Underweight			Overweight/obesity		
**Study size/participants, n**	**Pooled estimate, % (95% CI)**	***P*-value**	**Study size/participants, n**	**Pooled estimate, % (95% CI)**	***P*-value**
Meta regression analysis						
*Mean age*	16/26 244	2.2 (–3.9, 8.3)	0.480	40/118 756	5.1 (3.2, 7.0)	<0.001
*Length of stay*	9/9184	3.3 (–1.9, 8.5)	0.215	14/60 620	–0.1 (–3.1, 2.9)	0.0951
*Female*	16/7987	6.6 (5.4, 7.7)	<0.001	41/54 041	4.8 (4.5, 5.2)	<0.001
**Subgroup analysis**						
Study characteristics						
*Cohort study*	1/76	7.9 (3.6, 16.5)	<0.001	1/76	32.9 (23.3, 44.2)	<0.001
*Cross-sectional study*	21/26 128	4.6 (3.0, 6.9)		47/135 328	43.9 (39.9, 47.9)	
Publish year						
*2011–2015*	NA	NA	<0.001	13/100 486	32.7 (30.7, 34.7)	<0.001
*2016–2020*	10/12 309	5.7 (3.9, 8.4)		18/19 825	50.1 (40.7, 59.5)	
*2021–2025*	11/13 935	3.8 (2.0, 7.3)		15/15 093	44.7 (36.7, 52.9)	
Setting						
*Community*	17/15 409	6.0 (4.4, 8.2)	<0.001	42/124 278	42.3 (39.1, 45.5)	<0.001
*Hospital*	5/10 976	2.0 (1.0, 4.0)		6/11 126	53.7 (42.5, 64.5)	
Sample size category						
*<1000*	15/11 561	6.5 (4.8, 8.9)	<0.001	32/11 651	44.7 (37.4, 52.3)	0.003
*>1000*	7/14 593	2.5 (1.0, 6.0)		16/123 753	41.5 (35.8, 47.5)	
Study quality*						
*High*	17/16 407	4.0 (2.5, 6.5)	<0.001	41/126 308	43.4 (39.2, 47.7)	0.002
*Moderate*	4/9761	7.6(3.2, 16.9)		5/5212	44.6 (30.5, 59.6)	
*Low*	1/76	7.9 (3.6, 16.5)		2/3884	46.5 (22.5, 72.2)	
Destination country by continent						
*East Asia and the Pacific*	10/11 483	4.5 (2.7, 7.3)	<0.001	22/105 511	36.6 (33.7, 39.7)	<0.001
*Europe and Central Asia*	1/450	7.3(5.3, 10.1)		6/3463	65.2 (45.8, 80.6)	
*Latin America and the Caribbean*	4/938	7.6 (4.3, 13.3)		5/1084	48.9 (35.7, 62.1)	
*Middle East and North Africa*	4/11 549	1.3 (0.7, 2.4)		4/11 549	61.1 (48.9, 72.1)	
*North America*	NA	NA		6/4198	40.0 (25.1, 56.9)	
*South Asia*	3/8131	12.5 (10.4, 15.1)		3/8131	34.8 (30.2, 39.8)	
*Sub-Saharan Africa*	NA	NA		2/1459	42.8 (11.8, 80.7)	
Destination country by income level						
*High*	8/14 721	1.8 (1.2, 2.6)	<0.001	22/27 413	53.2 (43.9, 62.2)	<0.001
*Upper middle*	11/3392	7.3 (5.6, 9.5)		21/98 401	35.7 (33.9, 37.5)	
*Lower middle*	3/8131	12.5(10.4, 15.1)		5/9590	35.8 (26.6, 46.2)	
**Sample characteristics**						
Marital status						
*Single*	10/5332	25.1 (19.1, 32.4)	<0.001	15/16 754	22.8 (17.3, 29.6)	<0.001
*Married*	10/5332	65.5 (49.1, 78.9)		15/16 754	69.0 (57.3, 78.7)	
*Widow/widower*	4/2296	24.4 (16.8, 34.0)		5/1109	19.1 (12.1, 28.7)	
Education level†						
*Lower than secondary level*	12/7697	31.3 (18.5, 47.9)	0.868	34/117 314	49.2 (44.5, 53.9)	0.761
*More than equal secondary level*	12/7697	73.5 (56.4, 85.6)		34/117 314	51.8 (47.1, 56.5)	
Migrant setting						
*Rural*	2/7285	0.8 (0.6, 1.0)	<0.001	12/15 006	40.4 (28.6, 53.4)	0.011
*Urban*	19/15 485	5.8 (4.1, 8.1)		35/120 398	45.3 (41.2, 49.5)	

CI – confidence interval, NA – not applicable

*The Joanna Briggs Institute (JBI) quality assessment tool was used to evaluate the quality of the included studies, with the total score divided into three categories: high quality, with scores of >70.0%; moderate quality, with scores 50.0–70.0%; low Quality, with scores <50.0%.

†Education level:<secondary level, lower than high school; ≥secondary level, graduate and higher than high school.

Subgroup analyses identified several factors moderated with the prevalence of underweight among migrant workers. Studies published between 2016 and 2020 reported a prevalence of 5.7% (95% CI = 3.9, 8.4). Community-based studies showed a higher pooled prevalence (6.0%; 95% CI = 4.4, 8.2). Studies with sample sizes of fewer than 1000 participants reported a prevalence of 6.5% (95% CI = 4.8, 8.9), while studies classified as low quality reported a prevalence of 7.9% (95% CI = 3.6, 16.5). When stratified by region, the highest prevalence was observed in South Asia (12.5%; 95% CI = 10.4, 15.1). Similarly, studies conducted in lower–middle-income countries reported a prevalence of 12.5% (95% CI = 10.4, 15.1). Sociodemographic factors also showed variation, with higher prevalence reported among married migrant workers (65.5%; 95% CI = 49.1, 78.9) and those working in urban settings (5.8%; 95% CI = 4.1, 8.1) (Appendix S11–23 in the **Online Supplementary Document**).

#### Overweight/obesity among migrant workers

We conducted meta-regression and subgroup analyses to explore potential sources of heterogeneity among the included studies. Meta-regression indicated that mean age (5.1%; 95% CI = 3.2, 7.0) and the proportion of female participants (4.8%; 95% CI = 4.5, 5.2) were significantly associated with the prevalence of overweight or obesity among migrant workers, whereas length of stay was not significantly associated with the outcome (−0.1%; 95% CI = –3.1, 2.9 (**Table 2**).

Subgroup analyses showed variation in prevalence across study characteristics and population contexts. Studies published between 2016 and 2020 reported a prevalence of 50.1% (95% CI = 40.7, 59.5). Hospital-based studies reported a higher prevalence (53.7%; 95% CI = 42.5, 64.5) than other study settings. When stratified by destination region, the highest prevalence was observed in Europe and Central Asia (65.2%; 95% CI = 45.8, 80.6). Similarly, studies conducted in high-income countries reported a prevalence of 53.2% (95% CI = 43.9, 62.2). Higher prevalence was also observed among married migrant workers (69.0%; 95% CI = 57.3, 78.7) (Appendix S24–36 in the **Online Supplementary Document**).

## DISCUSSION

In this meta-analysis, we highlighted the double burden of malnutrition among local and international migrant workers, with an underweight prevalence of 4.7% and an overweight/obesity prevalence of 43.7%. Subgroup analyses suggested that underweight was more prevalent among local migrants, especially in lower-middle-income countries and regions like South Asia, while overweight/obesity prevalence was highest among international migrants, particularly in high-income countries and Europe and Central Asia. These subgroup patterns should be interpreted cautiously because they are based on study-level comparisons, involve multiple stratifications and are primarily intended to explore heterogeneity rather than test a single causal model.

Considerable between-study heterogeneity is expected in global prevalence meta-analyses because underlying prevalence genuinely varies across settings, populations, and measurement approaches. High heterogeneity, therefore, does not in itself invalidate the pooled estimates but indicates that they should be interpreted cautiously as averages across heterogeneous contexts rather than as precise global values. In this study, heterogeneity likely arose from differences in migrant type, migration setting, destination region, and country income level, as well as methodological variation in study design, setting, sample size, quality, and BMI measurement or cut-off definitions. To account for this, we used random-effects models and prediction intervals, supported by leave-one-out sensitivity analyses, exclusion of cohort studies, subgroup analyses, and meta-regression to assess robustness and explore sources of variability [23,24,58–60]. These methods can identify influential studies and coherent subgroup patterns, but they cannot eliminate genuine contextual variation. The pooled estimates should therefore be viewed as context-dependent summary averages, with interpretation guided more by the prediction intervals and consistency of subgroup findings than by the pooled point estimates alone.

The distinction between local and international migration may partly reflect differences in the migration context. Local migrants, particularly those moving from rural to urban areas within the same country, may continue to experience structural disadvantages, including poor housing, informal or physically demanding work, and limited access to subsidised food or health programmes [61]. These conditions may preserve nutritional vulnerability despite geographic relocation. By contrast, international migrants may have access to higher incomes, but may also face new risks, such as dietary acculturation, limited nutrition knowledge, and more sedentary occupational roles [42,49,62]. However, this interpretation should be made cautiously, as evidence on internal migration was more limited and concentrated in specific settings, particularly China and India, which may reduce the generalisability of comparisons between internal and international migrants [56,61]. At the same time, international labour migration is often shaped by legal and institutional constraints, including restrictive work contracts, tied employment arrangements, and limited access to public health services, which may adversely affect diet quality and longer-term health outcomes [18,63].

Several study-level characteristics were significantly associated with the prevalence of underweight, including the proportion of female participants, study design, publication year, study setting, sample size category, study quality, destination country or region, host-country income level, marital status, and migrant setting (rural *vs.* urban). These patterns may reflect both contextual differences across migrant populations and methodological variation across studies, rather than direct causal mechanisms. For example, higher underweight prevalence in community-based than hospital-based studies, and in smaller studies, may indicate differences in sampling frames or the inclusion of more socially or economically vulnerable groups [15,54]. Underweight prevalence was also higher in lower-middle-income destinations, South Asia, and urban migrant settings, which may be consistent with differences in food affordability, cost of living, work demands, and access to services across settings, but these interpretations should be viewed as hypotheses rather than demonstrated mechanisms [64].

For overweight or obesity, meta-regression showed that mean age and the proportion of female participants were significant moderators. These findings are consistent with broader evidence suggesting that age- and sex-related differences may shape weight patterns [65,66]. Subgroup analyses also showed significant variation by study design, publication year, study setting, destination country or region, host-country income level, and marital status. Overweight or obesity prevalence was higher in hospital-based than community-based studies, which may reflect differences in case mix, healthcare access, or sampling approaches rather than true population differences alone [18]. The higher prevalence observed in high-income destinations, particularly Europe and Central Asia, may be consistent with more obesogenic environments and nutrition transition patterns, including greater availability of energy-dense foods and lower levels of physical activity [62,63]. Length of stay was not a significant moderator for either outcome, suggesting that duration alone may not adequately capture the broader structural and living conditions relevant to malnutrition risk [67].

Gender and marital status also showed significant variation in both outcomes. These subgroup patterns can be interpreted through the lens of the SDOH, which emphasises that health outcomes are shaped by structural, socioeconomic, and contextual conditions [29]. For women, the observed variation may be related to gendered occupational roles, social pressures, or other unmeasured contextual factors [68]. Similarly, the higher prevalence observed among married individuals may reflect differences in household roles, financial obligations, or lifestyle patterns, but these mechanisms were not directly evaluated in the included studies. For underweight, one possible explanation is that competing household demands or remittance obligations may influence dietary intake or food quality among migrant workers themselves [13]. For overweight or obesity, another possible explanation is that marital status may be associated with lifestyle changes, physical activity patterns, or stress-related eating behaviours [41,62,66]. However, because most included studies were cross-sectional, these interpretations should be considered hypothesis-generating and consistent with the observed patterns, rather than evidence of causal pathways.

From a nutrition policy perspective, the results underscore the urgent need to integrate nutritional considerations into migration and labour health policies. Routine nutritional screening, culturally tailored dietary education, and improved access to affordable, nutritious foods should be implemented in both sending and receiving countries. Occupational health systems must also embed nutrition services as part of migrant worker protections [1,64]. Given the variability across contexts, interventions are likely to require tailoring by migrant type, destination setting, and prevailing labour and food environments.

### Strengths and limitations

This study contributes to the global nutrition literature by being among the first meta-analyses to compare the prevalence of malnutrition between local and international migrant workers, while incorporating meta-regression and GRADE-based certainty assessments to strengthen the interpretation of the findings. However, several limitations should be acknowledged. First, this meta-analysis demonstrated statistical heterogeneity across included studies, likely reflecting differences in study populations, settings, and methodological approaches, which may also contribute to funnel plot asymmetry. Therefore, pooled prevalence estimates should be interpreted as context-dependent averages and considered alongside prediction intervals and subgroup patterns. Second, subgroup analyses were extensive and primarily intended to explore heterogeneity. Because multiple stratified comparisons were conducted across regions, income levels, study characteristics, and sociodemographic factors, these findings may be prone to spurious associations and should be interpreted with caution. We also acknowledge that multiple estimates from the same study may have introduced within-study correlation. As individual-level data were unavailable, this dependence could not be explicitly modelled, and we did not apply multilevel meta-analysis or robust variance estimation. Consequently, some standard errors may have been underestimated, and precision overestimated. Future meta-analyses should consider multilevel or robust variance estimation approaches to better account for statistical dependence among estimates derived from the same study.

In global health prevalence research, potential bias may arise not only from selective publication but also from structural characteristics of the evidence base. Small-study effects may lead to inflated or more variable prevalence estimates, as smaller studies often report larger or more extreme effects than larger studies [60,69]. Regional research imbalances may influence pooled estimates when evidence is concentrated in a limited number of geographic regions while other areas remain underrepresented, thereby limiting the extent to which pooled estimates reflect global patterns. Furthermore, publication patterns may introduce bias if studies reporting statistically significant or notable findings are more likely to be published or identified, potentially leading to missing evidence that could distort meta-analytic estimates [24]. In addition, funnel plot asymmetry should not be interpreted as evidence of publication bias alone, as it can also arise from heterogeneity, small-study effects, and methodological differences [60].

Geographical representation was limited, with underrepresentation of major migrant-receiving regions such as the Middle East and Sub-Saharan Africa, which may reduce the generalisability of the findings. Variation in BMI classification criteria across studies also represents an important limitation. Although most studies used WHO thresholds, some applied regional cut-offs, particularly in Asian populations, which may have reduced comparability, contributed to heterogeneity, and influenced the pooled prevalence estimates, especially in cross-regional comparisons. Future research should prioritise standardised data collection, more consistent reporting of key covariates, broader surveillance in underrepresented regions, and the use of analytic approaches that better address statistical dependence among effect estimates.

## CONCLUSIONS

This meta-analysis highlights the double burden of malnutrition among migrant workers, with pooled prevalences of 4.7% for underweight and 43.7% for overweight/obesity. These estimates should be interpreted with caution as averages across diverse settings rather than as fixed global values. Local migrants, especially in lower-income settings, tended to have higher undernutrition prevalence, whereas international migrants showed higher overweight/obesity prevalence, pointing to different contextual and occupational risks. Although several study-level moderators were identified, these findings should be regarded as hypothesis-generating given the substantial heterogeneity and multiple comparisons. The findings nevertheless underscore the need to embed nutrition-sensitive approaches within labour, migration, and public health policies, including regular nutritional screening, improved access to affordable healthy foods, and culturally appropriate dietary interventions. Future research should prioritise longitudinal studies, stronger methodological consistency, and context-specific interventions to better address the nutritional vulnerabilities of diverse migrant populations.

## Additional material


Online Supplementary Document
