# Supplementary material for: Global prevalence of underweight and overweight/obesity among migrant workers: a systematic review and meta-analysis
Source: J Glob Health. 2026 May 15;16:04154. doi: 10.7189/jogh.16.04154 (PMC13178055; doi:10.7189/jogh.16.04154)
Supplement: Online Supplementary Document [file jogh-16-04154-s001.pdf]

Supplement to: Nurrahma HA, Arifin H, Pradana AA, Susanto H, Yeh CL, Chen YL, Shirakawa H, Chou KR, Yang SC. Global prevalence of underweight and overweight/obesity among migrant workers: a systematic review and meta-analysis. J Glob Health. 2026;16:04154.

Global prevalence of underweight and overweight/obesity among migrant workers: a systematic review and meta-analysis

Appendix 1. Meta-analysis of Observational Studies in Epidemiology (MOOSE) 3
Appendix 2. Preferred Reporting Items for Systematic Reviews and Meta-Analyses 5
Appendix 3. Search Terms Used in Cochrane, Embase, Scopus, Pubmed, Web of Science, LILACS and African Index Mediscus 8
Appendix 4. Search Syntaxes Used in Databases 10
Appendix 5. Operational classification of migrant worker populations included in this review 13
Appendix 6. Demographics and Overall Characteristics of Included Studies 14
Appendix 7. Study Quality 15
Appendix 8. GRADE Summary of Findings – Prevalence of Malnutrition Among Migrant Workers 18
Appendix 9. Funnel Plot of Underweight among Migrant Workers 19
Appendix 10. Duval and Tweedie trim-and-fill analysis for publication bias in studies reporting the prevalence of underweight among migrant workers. 20
Appendix 11. Funnel Plot of Overweight/Obesity among Migrant Workers 21
Appendix 12. Duval and Tweedie trim-and-fill analysis for publication bias in studies reporting the prevalence of overweight/obesity among migrant workers. 22
Appendix 13. Meta-regression Scatter Plot of Underweight among Migrant Workers with Age 23
Appendix 14. Meta-regression Scatter Plot of Underweight among Migrant Workers with Length of Stay ... 24
Appendix 15. Meta-regression Scatter Plot of Underweight among Migrant Workers with Female 25
Appendix 16. Pooled Prevalence of Underweight among Migrant Workers based on Study design 26
Appendix 17. The Pooled Prevalence of Underweight among Migrant Workers by Publish Year of Studies .. 27
Appendix 18. Pooled Prevalence of Underweight among Migrant Workers according to Study Settings 28
Appendix 19. Pooled Prevalence of Underweight among Migrant Workers based on Sample size category .. 29
Appendix 20. Pooled Prevalence of Underweight among Migrant Workers according to Study Quality 30
Appendix 21. Pooled Prevalence of Underweight among Migrant Workers based on Destination Continent . 31
Appendix 22. Pooled Prevalence of Underweight among Migrant Workers based on income level of destination country 32
Appendix 23. Pooled Prevalence of Underweight among Migrant Workers according by Marital status 33
Appendix 24. Pooled Prevalence of Underweight among Migrant Workers by Education Level 34
Appendix 25. Pooled Prevalence of Underweight among Migrant Workers according to Migrant Setting 35
Appendix 26. Pooled Prevalence of Underweight among Migrant Workers according to Migrant Occupational 36
Appendix 27. Pooled Prevalence of Underweight among Migrant Workers according to Migrant Duration .. 37
Appendix 28. Meta-regression Scatter Plot of Overweight/Obesity among Migrant Workers with Mean Age 38
Appendix 29. Meta-regression Scatter Plot of Overweight/Obesity among Migrant Workers with Length of Stay 39
Appendix 30. Meta-regression Scatter Plot of Overweight/Obesity among Migrant Workers with Female 40

Appendix 31. The Pooled Prevalence of Overweight/Obesity among Migrant Workers based on Study design 41
Appendix 32. Pooled Prevalence of Overweight/Obesity among Migrant Workers by Publish Year of Studies. 42
Appendix 33. Pooled Prevalence of Overweight/Obesity among Migrant Workers according to Study Settings 43
Appendix 34. Pooled Prevalence of Overweight/Obesity among Migrant Workers based on Sample size category 44
Appendix 35. Pooled Prevalence of Overweight/Obesity among Migrant Workers according to Study Quality 45
Appendix 36. Pooled Prevalence of Overweight/Obesity among Migrant Workers based on Destination Continent 46
Appendix 37. Pooled Prevalence of Overweight/Obesity among Migrant Workers based on income level of destination country 47
Appendix 38. Pooled Prevalence of Overweight/Obesity among Migrant Workers according by Marital status 48
Appendix 39. Pooled Prevalence of Overweight/Obesity among Migrant Workers by Education Level 49
Appendix 40. Pooled Prevalence of Overweight/Obesity among Migrant Workers according to Migrant Setting 50

Appendix 1. Meta-analysis of Observational Studies in Epidemiology (MOOSE)

Item No	Recommendation	Reported on Page No
Reporting of background should include		
1	Problem definition	2-3
2	Hypothesis statement	N/A
3	Description of study outcome(s)	3
4	Type of exposure or intervention	4-6
5	Type of study designs	3-4
6	Study population	74
Reporting of search strategy should include		
7	Qualifications of searchers (eg, librarians and investigators)	4-5
8	Search strategy, including period included in the synthesis and key words	4, Supplementary
9	Effort to include all available studies, including contact with authors	4
10	Databases and registries searched	4-5
11	Search software used, name and version, including special features used (eg, explosion)	4
12	Use of hand searching (eg, reference lists of obtained articles)	4
13	List of citations located and those excluded, including justification	Supplementary
14	Method of addressing articles published in languages other than English	4
15	Method of handling abstracts and unpublished studies	4
16	Description of any contact with authors	4
Reporting of methods should include		
17	Description of relevance or appropriateness of studies assembled for assessing the hypothesis to be tested	4
18	Rationale for the selection and coding of data (eg, sound clinical principles or convenience)	4
19	Documentation of how data were classified and coded (eg, multiple raters, blinding and interrater reliability)	N/A
20	Assessment of confounding (eg, comparability of cases and controls in studies where appropriate)	N/A
21	Assessment of study quality, including blinding of quality assessors, stratification or regression on possible predictors of study results	4-5
22	Assessment of heterogeneity	5-6
23	Description of statistical methods (eg, complete description of fixed or random effects models, justification of whether the chosen models account for predictors of study results, dose–response models, or cumulative meta-analysis) in sufficient detail to be replicated	5-6
24	Provision of appropriate tables and graphics	22-43, Supplementary
Reporting of results should include		
25	Graphic summarizing individual study estimates and overall estimate	Supplementary
26	Table giving descriptive information for each study included	22-40
27	Results of sensitivity testing (eg, subgroup analysis)	41-43, Supplementary

28	Indication of statistical uncertainty of findings	N/A
Reporting of discussion should include		
29	Quantitative assessment of bias (eg, publication bias)	7-8, Supplementary
30	Justification for exclusion (eg, exclusion of non-English language citations)	4
31	Assessment of quality of included studies	9, supplementary
Reporting of conclusions should include		
32	Consideration of alternative explanations for observed results	9-13
33	Generalization of the conclusions (ie, appropriate for the data presented and within the domain of the literature review)	13
34	Guidelines for future research	13
35	Disclosure of funding source	13

MOOSE by Stroup DF, Berlin JA, Morton SC, Olkin I, Williamson GD, Rennie Det al. (1)

Appendix 2. Preferred Reporting Items for Systematic Reviews and Meta-Analyses

Item No	Checklist Item	Location where item is reported
Title		
1	Identify the report as a systematic review.	1
Abstract		
2	See the PRISMA 2020 for Abstracts checklist.	N/A
Introduction		
3	Describe the rationale for the review in the context of existing knowledge.	2-3
4	Provide an explicit statement of the objective(s) or question(s) the review addresses.	2-3
Methods		
5	Specify the inclusion and exclusion criteria for the review and how studies were grouped for the syntheses.	4
6	Specify all databases, registers, websites, organisations, reference lists and other sources searched or consulted to identify studies. Specify the date when each source was last searched or consulted.	3-4
7	Present the full search strategies for all databases, registers and websites, including any filters and limits used.	Supplementary
8	Specify the methods used to decide whether a study met the inclusion criteria of the review, including how many reviewers screened each record and each report retrieved, whether they worked independently, and if applicable, details of automation tools used in the process.	3-4
9	Specify the methods used to collect data from reports, including how many reviewers collected data from each report, whether they worked independently, any processes for obtaining or confirming data from study investigators, and if applicable, details of automation tools used in the process.	3-4
10a	List and define all outcomes for which data were sought. Specify whether all results that were compatible with each outcome domain in each study were sought (e.g. for all measures, time points, analyses), and if not, the methods used to decide which results to collect.	4-5, Figure
10b	List and define all other variables for which data were sought (e.g. participant and intervention characteristics, funding sources). Describe any assumptions made about any missing or unclear information.	4-5, Figure
11	Specify the methods used to assess risk of bias in the included studies, including details of the tool(s) used, how many reviewers assessed each study and whether they worked independently, and if applicable, details of automation tools used in the process.	5-6
12	Specify for each outcome the effect measure(s) (e.g. risk ratio, mean difference) used in the synthesis or presentation of results.	5-6
13a	Describe the processes used to decide which studies were eligible for each synthesis (e.g. tabulating the study intervention characteristics and comparing against the planned groups for each synthesis (item #5)).	5-6
13b	Describe any methods required to prepare the data for presentation or synthesis, such as handling of missing summary statistics, or data conversions.	5-6

13c	Describe any methods used to tabulate or visually display results of individual studies and syntheses.	5-6
13d	Describe any methods used to synthesize results and provide a rationale for the choice(s). If meta-analysis was performed, describe the model(s), method(s) to identify the presence and extent of statistical heterogeneity, and software package(s) used.	5-6
13e	Describe any methods used to explore possible causes of heterogeneity among study results (e.g. subgroup analysis, meta-regression).	6-7
13f	Describe any sensitivity analyses conducted to assess robustness of the synthesized results.	7
14	Describe any methods used to assess risk of bias due to missing results in a synthesis (arising from reporting biases).	7
15	Describe any methods used to assess certainty (or confidence) in the body of evidence for an outcome.	7
Results		
16a	Describe the results of the search and selection process, from the number of records identified in the search to the number of studies included in the review, ideally using a flow diagram.	6, Figure
16b	Cite studies that might appear to meet the inclusion criteria, but which were excluded, and explain why they were excluded.	6, Figure
17	Cite each included study and present its characteristics.	22-40
18	Present assessments of risk of bias for each included study.	11, Supplementary
19	For all outcomes, present, for each study: (a) summary statistics for each group (where appropriate) and (b) an effect estimate and its precision (e.g. confidence/credible interval), ideally using structured tables or plots.	22-40, Figure, Supplementary
20a	For each synthesis, briefly summarise the characteristics and risk of bias among contributing studies.	Supplementary
20b	Present results of all statistical syntheses conducted. If meta-analysis was done, present for each the summary estimate and its precision (e.g. confidence/credible interval) and measures of statistical heterogeneity. If comparing groups, describe the direction of the effect.	40-43, Table
20c	Present results of all investigations of possible causes of heterogeneity among study results.	40-43, Supplementary
20d	Present results of all sensitivity analyses conducted to assess the robustness of the synthesized results.	7
21	Present assessments of risk of bias due to missing results (arising from reporting biases) for each synthesis assessed.	7, Supplementary
22	Present assessments of certainty (or confidence) in the body of evidence for each outcome assessed.	7, Supplementary
Discussion		
23a	Provide a general interpretation of the results in the context of other evidence.	6-9
23b	Discuss any limitations of the evidence included in the review.	8-9
23c	Discuss any limitations of the review processes used.	8-9
23d	Discuss implications of the results for practice, policy, and future research.	9
Other Information		

24a	Provide registration information for the review, including register name and registration number, or state that the review was not registered.	3
24b	Indicate where the review protocol can be accessed, or state that a protocol was not prepared.	3
24c	Describe and explain any amendments to information provided at registration or in the protocol.	3
25	Describe sources of financial or non-financial support for the review, and the role of the funders or sponsors in the review.	13
26	Declare any competing interests of review authors.	13
27	Report which of the following are publicly available and where they can be found: template data collection forms; data extracted from included studies; data used for all analyses; analytic code; any other materials used in the review.	6-7, Supplementary

Checklist by PRISMA (2)

Appendix 3. Search Terms Used in Cochrane, Embase, Scopus, Pubmed, Web of Science, LILACS and African Index Mediscus

**Databases**	**Population**	**Exposure**	**Outcome**
**Cochrane**	transients and migrants (mesh)<br>migrant worker*<br>migrant*<br>nomad*<br>transient*	malnutrition (mesh)<br>malnourishment*<br>undernutrition<br>nutritional deficienc*<br>“deficiency disease*”<br>underweight<br>thinness<br>leanness<br>overweight (mesh)<br>obesity (mesh)<br>obese	prevalence<br>epidemiology<br>incidence
**Embase**	migrant worker (emtree)<br>transients and migrants<br>migrant worker*<br>migrant*<br>nomad*<br>transient*	malnutrition (emtree)<br>malnourishment*<br>undernutrition<br>nutritional deficiency (emtree)<br>nutritional disorder (emtree)<br>“deficiency disease*”<br>underweight<br>thinness<br>leanness<br>overweight<br>obesity (emtree)<br>obese	prevalence<br>epidemiology<br>incidence
**Scopus**	transients and migrants (mesh)<br>migrant worker*<br>migrant*<br>nomad*<br>transient*	malnutrition (mesh)<br>malnourishment*<br>undernutrition<br>nutritional deficienc*<br>“deficiency disease*”<br>underweight<br>thinness<br>leanness<br>overweight (mesh)<br>obesity (mesh)<br>obese	prevalence<br>epidemiology<br>incidence
**PubMed**	transients and migrants (mesh)<br>migrant worker*	malnutrition (mesh)<br>malnourishment*<br>undernutrition	prevalence<br>epidemiology<br>incidence

	migrant*<br>nomad*<br>transient*	“nutritional deficienc*”<br>“deficiency disease*”<br>underweight<br>thinness<br>leanness<br>overweight (mesh)<br>obesity (mesh)<br>obese	
**Web of Science**	transients and migrants (mesh)<br>migrant worker*<br>migrant*<br>nomad*<br>transient*	malnutrition (mesh)<br>malnourishment*<br>undernutrition<br>nutritional deficienc*<br>“deficiency disease*”<br>underweight<br>thinness<br>leanness<br>overweight (mesh)<br>obesity (mesh)<br>obese	prevalence<br>epidemiology<br>incidence
**LILACS**	migrant<br>migration<br>migrante<br>worker<br>labor<br>trabajo	nutrition<br>underweight<br>overweight<br>obesity	prevalence<br>epidemiology<br>incidence
**African Index Mediscus**	migrant worker<br>migrant labor<br>migrant	nutrition<br>underweight<br>overweight<br>obesity	prevalence<br>epidemiology<br>incidence

Appendix 4. Search Syntaxes Used in Databases

Databases	Syntaxes	Number
**Cochrane**	#1: MeSH descriptor: [Transients and Migrants] explode all trees	141
	#2: (transients and migrants)	154
	#3: (migrant worker*)	161
	#4: (transient worker*)	217
	#5: (labor migrant*)	62
	#6: (foreign worker*)	185
	#7: (economic migrant*)	96
	#8: (seasonal worker*)	249
	#9: (immigrant*)	1200
	#10: (migrant*)	701
	#11: (laborer)	25
	#12: (nomad*)	79
	#13: (transient*)	24008
	#14: #1 OR #2 OR #3 OR #4 OR #5 OR #6 OR #7 OR #8 OR #9 OR #10 OR #11 OR #12 OR #13	26021
	#15: MeSH descriptor: [Malnutrition] explode all trees	6287
	#16: (malnutrition)	7697
	#17: (malnourishment*)	103
	#18: (undernutrition)	931
	#19: (nutritional deficienc*)	3686
	#20: (deficiency disease*)	13047
	#21: (underweight)	1617
	#22: (thinness)	631
	#23: (leanness)	25
	#24: #15 OR #16 OR #17 OR #18 OR #19 OR #20 OR #21 OR #22 OR #23	26557
	#25: MeSH descriptor: [Overweight] explode all trees	26704
	#26: (overweight)	25754
	#27: #25 OR #26	40799
	#28: MeSH descriptor: [Obesity] explode all trees	22904
	#29: (obesity)	60016
	#30: (obese)	30486
	#31: #28 OR #29 OR #30	67638
	#32: #24 OR #27 OR #31	96226
	#33: MeSH descriptor: [Prevalence] explode all trees	7385
	#34: (prevalence)	55765
	#35: MeSH descriptor: [Epidemiology] explode all trees	103
	#36: (epidemiology)	92004
	#37: MeSH descriptor: [Incidence] explode all trees	14393
	#38: (incidence)	179354
	#39: #33 OR #34 OR #35 OR #36 OR #37 OR #38	281547
	#40: #14 AND #32 AND #39	773
	trials	263

Databases	Syntaxes	Number
**Embase**	#1: 'migrant worker'/exp OR 'migrant worker*':ab,ti OR (transients:ab,ti AND migrants:ab,ti) OR 'migrant*':ab,ti OR 'nomads':ab,ti OR transient*:ab,ti	554,002
	#2: 'malnutrition'/exp OR 'malnutrition':ab,ti OR malnourishment:ab,ti OR undernutrition:ab,ti OR 'nutritional deficiency'/exp OR 'nutritional deficiency':ab,ti OR 'nutritional disorder'/exp OR 'nutritional disorder':ab,ti OR 'deficiency disease*':ab,ti OR underweight:ab,ti OR leanness:ab,ti	1,555,972
	#3: overweight:ab,ti	163,097
	#4: 'obesity'/exp OR 'obesity':ab,ti OR obese:ab,ti	959,823
	#5: #2 OR #3 OR #4	1,687,105
	#6: ('prevalence'/exp OR 'prevalence':ab,ti OR 'epidemiology'/exp OR epidemiology:ab,ti OR 'incidence'/exp) AND 'incidence':ab,ti	936,960
	#7: #1 AND #5 AND #6	1,170
**Pubmed**	#1: ((((((((malnutrition[MeSH Terms]) OR (malnutrition[Text Word])) OR (malnourishment*[Text Word])) OR (undernutrition[Text Word])) OR ("nutritional deficienc*"[Text Word])) OR ("deficiency disease*"[Text Word])) OR (underweight[Text Word])) OR (thinness[Text Word])) OR (leanness[Text Word])	217,961
	#2: (overweight[MeSH Terms]) OR (overweight[Text Word])	346,489
	#3: ((obesity[MeSH Terms]) OR (obesity[Text Word])) OR (obese[Text Word])	515,316
	#4: ((((("transients and migrants"[MeSH Terms]) OR ("transients and migrants"[Text Word])) OR ("migrant worker*"[Text Word])) OR (migrant*[Text Word])) OR (nomad*[Text Word])) OR (transient*[Text Word])	460,554
	#5: (((((prevalence[MeSH Terms]) OR (prevalence[Text Word])) OR (epidemiology[MeSH Terms])) OR (epidemiology[Text Word])) OR (incidence[MeSH Terms])) OR (incidence[Text Word])	3,724,863
	#6: ((((((((((malnutrition[MeSH Terms]) OR (malnutrition[Text Word])) OR (malnourishment*[Text Word])) OR (undernutrition[Text Word])) OR ("nutritional deficienc*"[Text Word])) OR ("deficiency disease*"[Text Word])) OR (underweight[Text Word])) OR (thinness[Text Word])) OR (leanness[Text Word])) OR ((overweight[MeSH Terms]) OR (overweight[Text Word]))) OR (((obesity[MeSH Terms]) OR (obesity[Text Word])) OR (obese[Text Word]))	729,434
	#7: ((((((("transients and migrants"[MeSH Terms]) OR ("transients and migrants"[Text Word])) OR ("migrant worker*"[Text Word])) OR (migrant*[Text Word])) OR (nomad*[Text Word])) OR (transient*[Text Word])) AND ((((((prevalence[MeSH Terms]) OR (prevalence[Text Word])) OR (epidemiology[MeSH Terms])) OR (epidemiology[Text Word])) OR (incidence[MeSH Terms])) OR (incidence[Text Word]))) AND ((((((((((malnutrition[MeSH Terms]) OR (malnutrition[Text Word])) OR (malnourishment*[Text Word])) OR (undernutrition[Text	2,070

Databases	Syntaxes	Number
	Word])) OR ("nutritional deficienc*"[Text Word])) OR ("deficiency disease*"[Text Word])) OR (underweight[Text Word])) OR (thinness[Text Word])) OR (leanness[Text Word])) OR ((overweight[MeSH Terms]) OR (overweight[Text Word]))) OR (((obesity[MeSH Terms]) OR (obesity[Text Word])) OR (obese[Text Word])))	
**Scopus**	#1: ((TITLE-ABS-KEY (transients AND migrants) OR TITLE-ABS-KEY (migrant AND worker*) OR TITLE-ABS-KEY (migrant*) OR TITLE-ABS-KEY (nomad*) OR TITLE-ABS-KEY (transient*)))	1,188,916
	#2: (TITLE-ABS-KEY (malnutrition) OR TITLE-ABS-KEY (malnourishment*) OR TITLE-ABS-KEY (undernutrition) OR TITLE-ABS-KEY (nutritional) OR TITLE-ABS-KEY (deficienc*) OR TITLE-ABS-KEY ("deficiency disease*") OR TITLE-ABS-KEY (underweight) OR TITLE-ABS-KEY (thinness) OR TITLE-ABS-KEY (leanness))	1,680,534
	#3: TITLE-ABS-KEY (overweight)	135,378
	#4: (TITLE-ABS-KEY (obesity) OR TITLE-ABS-KEY (obese))	752,008
	#5: (TITLE-ABS-KEY (prevalence) OR TITLE-ABS-KEY (epidemiology) OR TITLE-ABS-KEY (incidence))	3,805,700
	#6: #2 OR #3 OR #4	2,344,681
	#7: #1 AND #5 AND #6	5,280
**Web of Science**	#1: ((((ALL=(transients and migrants)) OR ALL=(migrant worker*)) OR ALL=(migrant*)) OR ALL=(nomad*)) OR ALL=(transient*)	790,783
	#2: (((((((ALL=(malnutrition)) OR ALL=(malnourishment*)) OR ALL=(undernutrition)) OR ALL=("nutritional deficienc*")) OR ALL=(“deficiency disease*”)) OR ALL=(underweight)) OR ALL=(thinness)) OR ALL=(leanness)	102,279
	#3: ALL=(overweight)	134,975
	#4: (ALL=(obesity)) OR ALL=(obese)	631,443
	#5: ((ALL=(prevalence)) OR ALL=(epidemiology)) OR ALL=(incidence)	2,710,408
	#6: #2 OR #3 OR #4	737,331
	#7: #1 AND #5 AND #6	1,746
**LILACS**	#1: (migrant OR migration OR migrante)	37,182
	#2: (worker OR labor OR trabajo)	180,255
	#3: (nutrition OR underweight OR overweight OR obesity)	98,816
	#4: #1 AND #2 AND #3	270
**African Index Mediscus**	#1: migrant worker OR migrant labor OR migrant	38
	#2: nutrition OR obesity OR overweight	735
	#3: #1 AND #2	21

Appendix 5. Operational classification of migrant worker populations included in this review

Category	Operational Definition	Example from included studies	Sources
Internal rural–urban migrant workers	Individuals relocating within national borders for employment, typically from rural to urban areas	Migrant factory workers in China; rural migrants working in urban manufacturing sectors	IOM Migration Glossary[3]; ILO Labour Migration Framework[4]
International migrant workers	Individuals who cross international borders for employment in another country	Domestic workers in Taiwan; migrant construction workers in Gulf countries	ILO Media-Friendly Glossary on Migration[5]
Seasonal migrant workers	Migrant workers whose employment is tied to seasonal economic activities	Agricultural harvest workers	UN ICMW Convention[6]
Temporary contract migrant workers	Migrant workers employed under fixed-term contracts or project-based employment	Contract-based migrant labor in construction or domestic work sectors	ILO Temporary Labour Migration Framework[7]

Appendix 6. Demographics and Overall Characteristics of Included Studies

Characteristics	Total, n (%)
Sample size, n	135,404
Age, mean ± standard deviation	32.34 ± 11.1
Study Design	
Cohort	1 (2.1)
Cross-sectional	47 (97.9)
Year Published	
2011 - 2015	13 (28.3)
2016 - 2020	18 (39.1)
2021 - 2025	15 (32.6)
Destination Country by Continent	
East Asia & Pacific	22 (45.8)
Europe & Central Asia	6 (12.5)
Latin America & Caribbean	5 (10.4)
Middle East & North Africa	4 (8.3)
North America	6 (12.5)
South Asia	3 (6.3)
Sub-Saharan Africa	2 (4.2)
Destination Country by Income Level	
High Income	22 (45.8)
Lower middle income	5 (10.4)
Upper middle income	21 (43.75)
Screening Tools	
WHO	41 (85.4)
APG	5 (10.4)
WPRO	2 (4.2)

WHO, World Health Organization; APG, Asia Pacific Guidelines

Appendix 7. Study Quality

Two reviewers independently evaluated the quality of the studies included in the analysis using the Joanna Briggs Institute quality assessment tool [8,9]. This tool comprises nine criteria that assess various elements such as sampling techniques, study participants, data collection, measurement, statistical analysis, and classification. Each criterion is rated with one of four options: Yes, No, Unclear, or Not Applicable. A Yes rating signifies that the criterion is met. The greater the number of Yes ratings, the higher the study's quality and the lower its risk of bias. The total number of Yes ratings was divided into three quality levels: high quality (>70%), moderate quality (50–70%), and low quality (<50%) [10]. Any disagreements between the reviewers during the quality assessment process were resolved through consultation with a third expert.

Study quality by the Joanna Briggs Institute tools include the following questions:

1. Was the sample frame appropriate to address the target population?
2. Were study participants sampled in an appropriate way?
3. Was the sample size adequate?
4. Were the study subjects and the setting described in detail?
5. Was the data analysis conducted with sufficient coverage of the identified sample?
6. Were valid methods used for the identification of the condition?
7. Was the condition measured in a standard, reliable way for all participants?
8. Was there appropriate statistical analysis?
9. Was the response rate adequate, and if not, was the low response rate managed appropriately?

No	Author	Risk of Bias									Quality
		1	2	3	4	5	6	7	8	9	
1	Abasilim C, Friedman LS, Martin MC, Madigan D, Perez J, Morera Met al. (11)	Yes	Yes	Yes	Yes	Yes	Yes	Yes	Yes	Yes	High Quality
2	Afrifa-Anane E, A DE-GA, K ACM, Beune E, Addo J, Smeeth Let al. (12)	Yes	Yes	Yes	Yes	Yes	Yes	Yes	Yes	Yes	High Quality
3	Alhadlaq HW, Ateeq A, Shayea AMF and Gasana J (13)	Yes	Yes	Yes	Yes	Yes	Yes	Yes	Yes	Yes	High Quality
4	Ali A, Alfajjam S and Gasana J (14)	Yes	Yes	Yes	Yes	Yes	Yes	Yes	Yes	Yes	High Quality
5	Arslan N, Çifci S and Bayram Değer V (15)	No	No	Yes	Yes	Yes	Yes	Unclear	Yes	Unclear	Moderate Quality
6	Aung TNN, Shirayama Y, Moolphate S, Aung MN, Lorga T and Yuasa M (16)	Yes	Yes	Yes	Yes	Yes	Yes	Yes	Yes	Yes	High Quality
7	Aung TNN, Shirayama Y, Moolphate S, Lorga T,	Yes	Yes	Yes	Yes	Yes	Yes	Yes	Yes	Yes	High Quality

	Angkurawaranon C, Yuasa Met al. (17)										
8	Bhandari P (18)	Yes	Yes	Unclear	Yes	Yes	Yes	Yes	Yes	Yes	High Quality
9	Bi Y, Wang L, Xu Y, Jiang Y, He J, Zhang Met al. (19)	Yes	Yes	Yes	Yes	Yes	Yes	Yes	Yes	Yes	High Quality
10	Carioca AAF, Gorgulho B, Teixeira JA, Fisberg RM and Marchioni DM (20)	Yes	Yes	Yes	Yes	Yes	Yes	Yes	Yes	Yes	High Quality
11	Carioca AAF, Gorgulho B, de Mello Fontanelli M, Fisberg RM and Marchioni DM (21)	Yes	Yes	Yes	Yes	Yes	Yes	Yes	Yes	Yes	High Quality
12	Castaneda SF, Rosenbaum RP, Holscher JT, Madanat H and Talavera GA (22)	Yes	Yes	Yes	Yes	Yes	Yes	Yes	Yes	Yes	High Quality
13	Castaneda J, Caire-Juvera G, Sandoval S, Castaneda PA, Contreras AD, Portillo GEet al. (23)	Yes	Yes	Yes	Yes	Yes	Yes	Yes	Yes	Yes	High Quality
14	Charoensook P, Upala P, Anuwatnonthakate A, Ruanjai T and Apidechkul T (24)	Yes	Yes	Yes	Yes	Yes	Yes	Yes	Yes	Yes	High Quality
15	Dah Poe NE, Srichan P, Khunthason S, Apidechkul T and Suttana W (25)	Yes	Yes	Yes	Yes	Yes	Yes	Yes	Yes	Yes	High Quality
16	Emiral GO, Onsuz MF, Ozay O, Isikli B and Metintas S (26)	Yes	Yes	Yes	Yes	Yes	Yes	Yes	Yes	Yes	High Quality
17	Hall BJ, Huang L, Yi G and Latkin C (27)	Unclear	Yes	Yes	Yes	Unclear	Yes	Yes	Yes	Unclear	Moderate Quality
18	Huang L, Chen W, Renzaho AMN and Hall BJ (28)	Yes	Yes	Unclear	Yes	Yes	Yes	Yes	Yes	Yes	High Quality
19	Jorgensen MB, Rasmussen CD, Carneiro IG, Flyvholm MA, Olesen K, Ekner Det al. (29)	Yes	Yes	Yes	Yes	Unclear	Yes	Yes	Unclear	Unclear	Moderate Quality
20	Joy EJ, Green R, Agrawal S, Aleksandrowicz L, Bowen L, Kinra Set al. (30)	Yes	Unclear	Yes	Yes	Unclear	Yes	Unclear	Unclear	Unclear	Moderate Quality
21	Khairizka Citra P, Jung-Su C and Chun-Kuang S (31)	Yes	Yes	Yes	Yes	Yes	Yes	Yes	Yes	Yes	High Quality
22	Kowalski K, Hoffman CJ and McClure A (32)	Yes	Unclear	Unclear	Yes	Unclear	Yes	Yes	Unclear	Unclear	Low Quality
23	Kuhn R, Barham T, Razzaque A and Turner P (33)	Yes	Yes	Yes	Yes	Yes	Yes	Yes	Yes	Unclear	High Quality
24	Leong CC (34)	Yes	Unclear	Unclear	Yes	Unclear	Yes	Yes	Unclear	Unclear	Low Quality

25	Lopez-Cevallos DF, Escutia G, Gonzalez-Pena Y and Garside LI (35)	Yes	Yes	Yes	Y es	Yes	Y es	Yes	Yes	Yes	High Quality
26	Mei CF, Faller EM, Chuan LX and Gabriel JS (36)	Yes	Yes	Yes	Y es	Yes	Y es	Yes	Yes	Yes	High Quality
27	Peng H, Sun M, Hu X, Han H, Su J, Peng Eet al. (37)	Yes	Yes	Yes	Y es	Yes	Y es	Yes	Yes	Yes	High Quality
28	Sanskriti J, Supriya V and Hemamalini AJ (38)	No	No	Unc lear	Y es	Yes	Ye s	Yes	Yes	No	Moderate Quality
29	Shan G, Wei D, Wang C, Zhang J, Wang B, Ma Met al. (39)	Yes	Yes	Yes	Y es	Yes	Y es	Yes	Yes	Yes	High Quality
30	Sornlorm K and Thi WM (40)	Yes	Yes	Yes	Y es	Yes	Y es	Yes	Yes	Yes	High Quality

Appendix 8. GRADE Summary of Findings – Prevalence of Malnutrition Among Migrant Workers

Two reviewers independently assessed the certainty of the evidence for each outcome using the GRADE approach, based on five domains: risk of bias, inconsistency, indirectness, imprecision, and publication bias [41]. Each domain was rated as having no limitation, serious limitation, or very serious limitation, with justifications provided in a summary table.

Certainty of evidence was then classified into four levels:

- High: very confident that the true effect lies close to that of the estimate
- Moderate:moderately confident in the effect estimate; the true effect is likely to be close, but may be substantially different
- Low: limited confidence; the true effect may be substantially different from the estimate
- Very low: very little confidence; the true effect is likely to be substantially different

Downgrading of certainty was applied if any of the five domains raised concerns (e.g., very high heterogeneity, small sample sizes, or potential publication bias), following Cochrane Handbook guidelines (Chapter 14) and GRADE Working Group recommendations. Discrepancies between reviewers were resolved through discussion with a third expert.

**Domain**	**Underweight**	**Overweight/Obesity**
Number of Study	22	48
Risk of Bias	Moderate: majority of studies were high quality, with minor concerns on sample size and dietary assessment methods	High: most studies were of high quality with minimal bias
Inconsistency	Serious: very high heterogeneity across studies (I^2 = 97.5%)	Serious: extremely high heterogeneity across studies (I^2 = 99.4%)
Indirectness	Not serious: studies were conducted among relevant migrant worker populations and used appropriate definitions	Not serious: studies clearly targeted migrant workers and used consistent, relevant outcome measures
Imprecision	Not serious: confidence interval (3.2%–7.3%) was narrow and interpretable	Not serious: confidence interval (39.7%–51.2%) remained within a clear range and supported meaningful interpretation
Publication Bias	Serious: funnel plot asymmetry and Peters' regression test (p 0.02) indicated potential publication bias	Serious: publication bias indicated by funnel plot and Peters' regression test (p 0.03)
Certainty	●●○○ Low: downgraded due to inconsistency and publication bias	●●○○ Low: downgraded due to inconsistency and publication bias

Appendix 9. Funnel Plot of Underweight among Migrant Workers

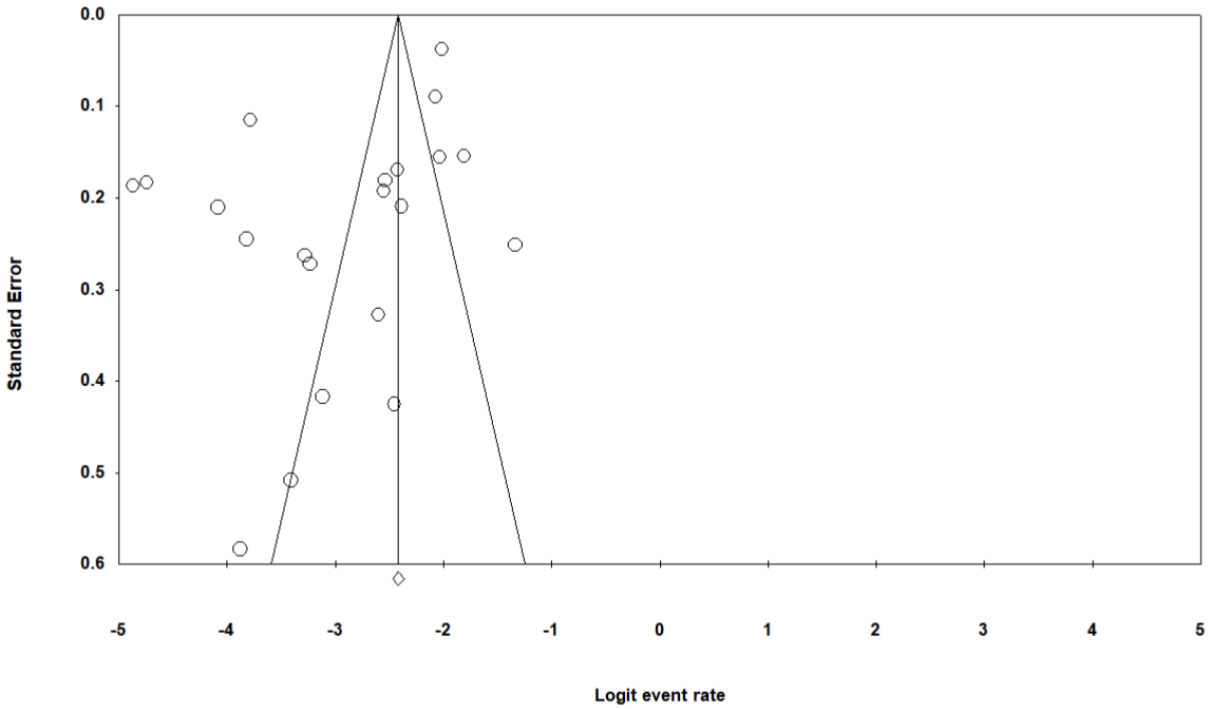

The plot shows visual asymmetry, suggesting potential publication bias, with fewer studies appearing on the left side of the mean effect size.

Appendix 10. Duval and Tweedie trim-and-fill analysis for publication bias in studies reporting the prevalence of underweight among migrant workers.

Duval and Tweedie's trim and fill

		Fixed Effects			Random Effects			Q Value
	Studies Trimmed	Point Estimate	Lower Limit	Upper Limit	Point Estimate	Lower Limit	Upper Limit	
**Observed values**		0.08173	0.07773	0.08591	0.04691	0.03146	0.06940	835.42178
**Adjusted values**	0	0.08173	0.07773	0.08591	0.04691	0.03146	0.06940	835.42178

Look for missing studies where?

- ○ Not specified
- ○ To left of mean
- ◉ To right of mean

Look for missing studies using which model?

- ○ Not specified
- ○ Fixed effect model
- ◉ Random effects model

Trim-and-fill analysis using a random-effects model identified no missing studies. The adjusted pooled estimate remained identical to the observed estimate, suggesting that potential publication bias had minimal influence on the pooled prevalence.

Appendix 11. Funnel Plot of Overweight/Obesity among Migrant Workers

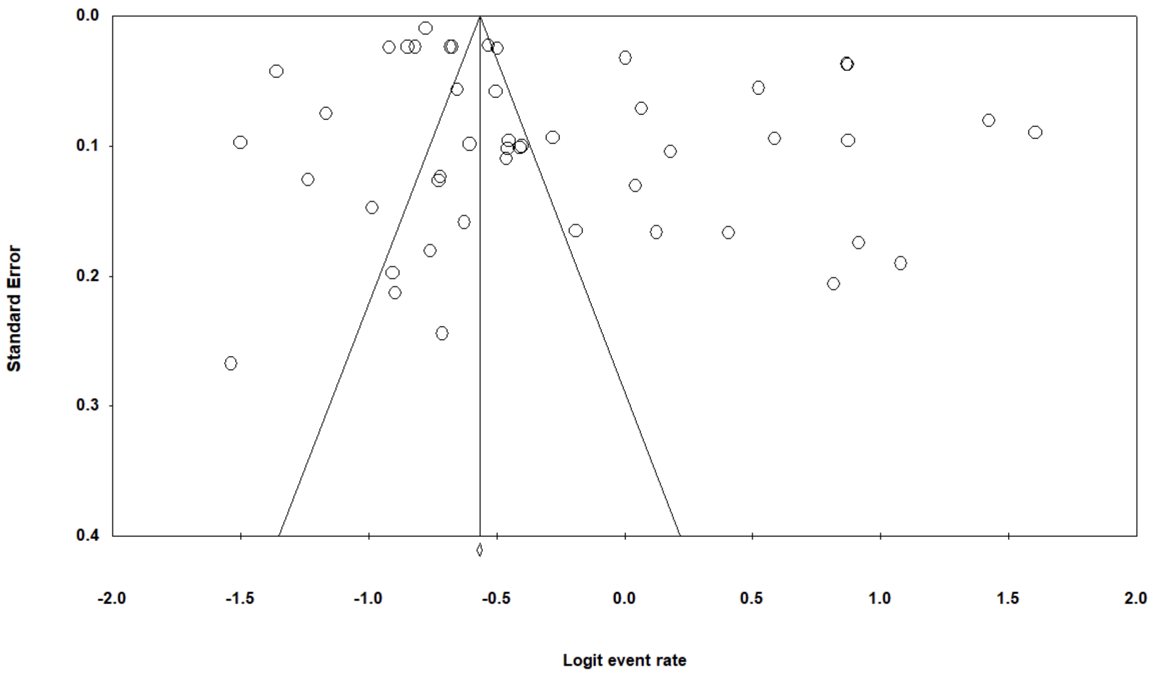

The plot shows visual asymmetry, suggesting potential publication bias, with fewer studies appearing on the right side of the mean effect size.

Appendix 12. Duval and Tweedie trim-and-fill analysis for publication bias in studies reporting the prevalence of overweight/obesity among migrant workers.

Duval and Tweedie's trim and fill

		**Fixed Effects**			**Random Effects**			**Q Value**
	Studies Trimmed	Point Estimate	Lower Limit	Upper Limit	Point Estimate	Lower Limit	Upper Limit	
**Observed values**		0.36193	0.35929	0.36458	0.43665	0.39779	0.47630	7595.83059
**Adjusted values**	5	0.37339	0.37076	0.37604	0.46774	0.42567	0.51026	9977.27200

Look for missing studies where?

- ○ Not specified
- ○ To left of mean
- ◉ To right of mean

Look for missing studies using which model?

- ○ Not specified
- ○ Fixed effect model
- ◉ Random effects model

Trim-and-fill analysis using a random-effects model estimated five potentially missing studies on the right side of the funnel plot. The adjusted pooled prevalence increased slightly from 43.7% to 46.7%, indicating that potential publication bias had limited influence on the overall findings.

Appendix 13. Meta-regression Scatter Plot of Underweight among Migrant Workers with Age

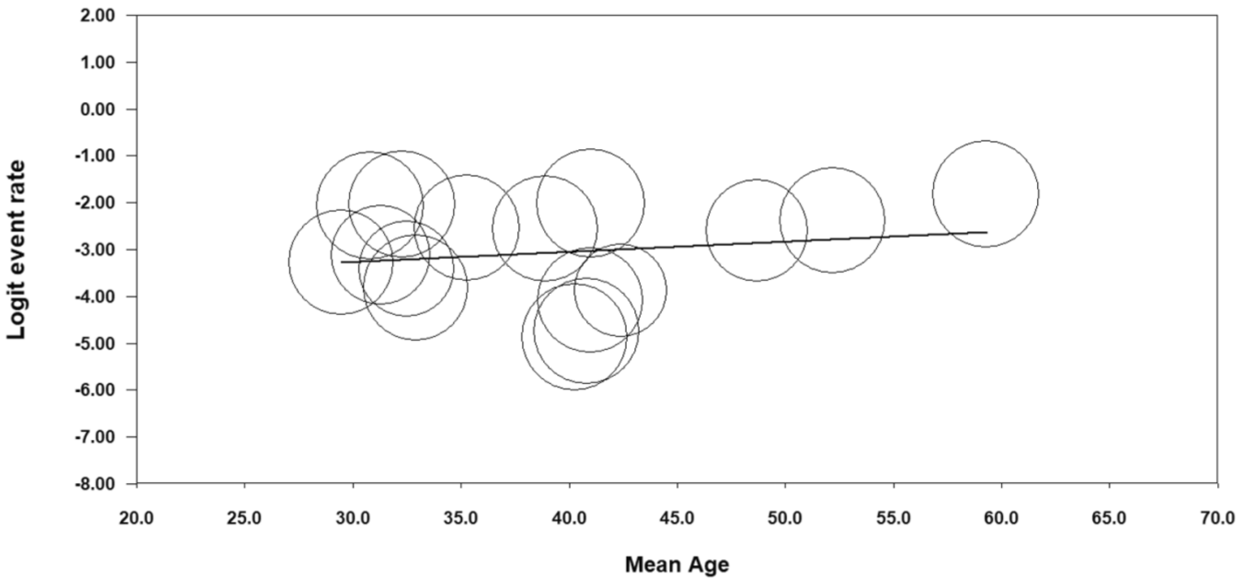

Meta-regression scatter plot showing the relationship between mean age and logit event rate across studies. The fitted regression line indicates a weak positive but non-significant association between mean age and logit event rate (β = 2.2%, 95% CI: –3.9 to 8.3, p value = 0.480, n = 26,244), suggesting that mean age does not significantly explain between-study variation in event rates.

Appendix 14. Meta-regression Scatter Plot of Underweight among Migrant Workers with Length of Stay

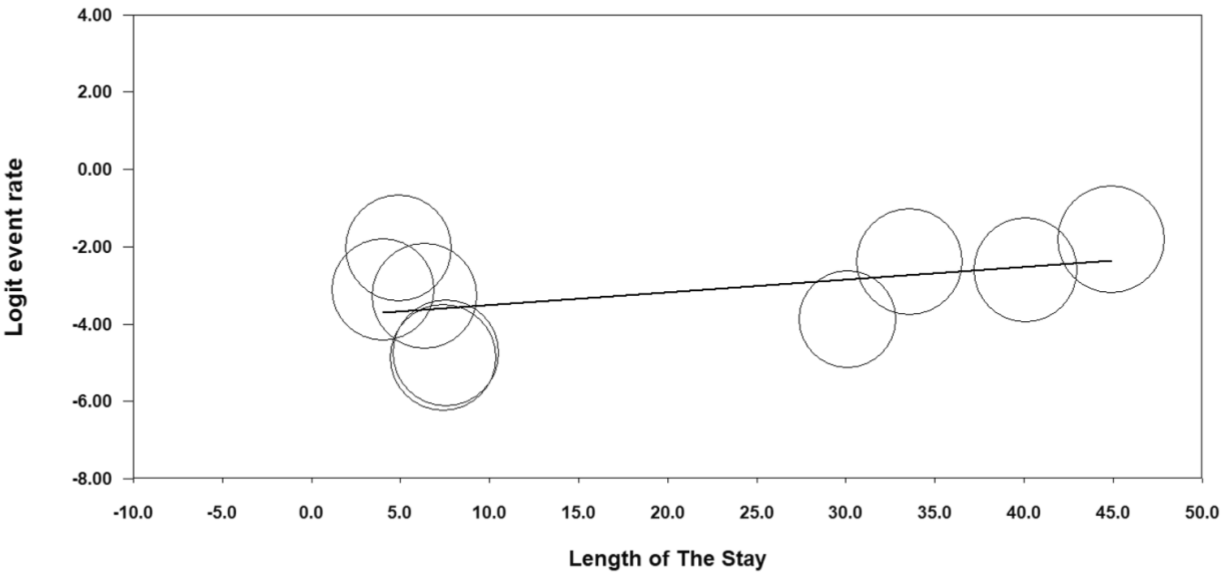

Meta-regression scatter plot showing the relationship between length of stay and logit event rate across studies. The fitted regression line indicates a weak but statistically significant positive association between length of stay and logit event rate (β = 3.3%, 95% CI: -1.9 to 8.5, p = 0.215, n =9,184), suggesting that length of stay not significantly explains between-study variation in event rates.

Appendix 15. Meta-regression Scatter Plot of Underweight among Migrant Workers with Female

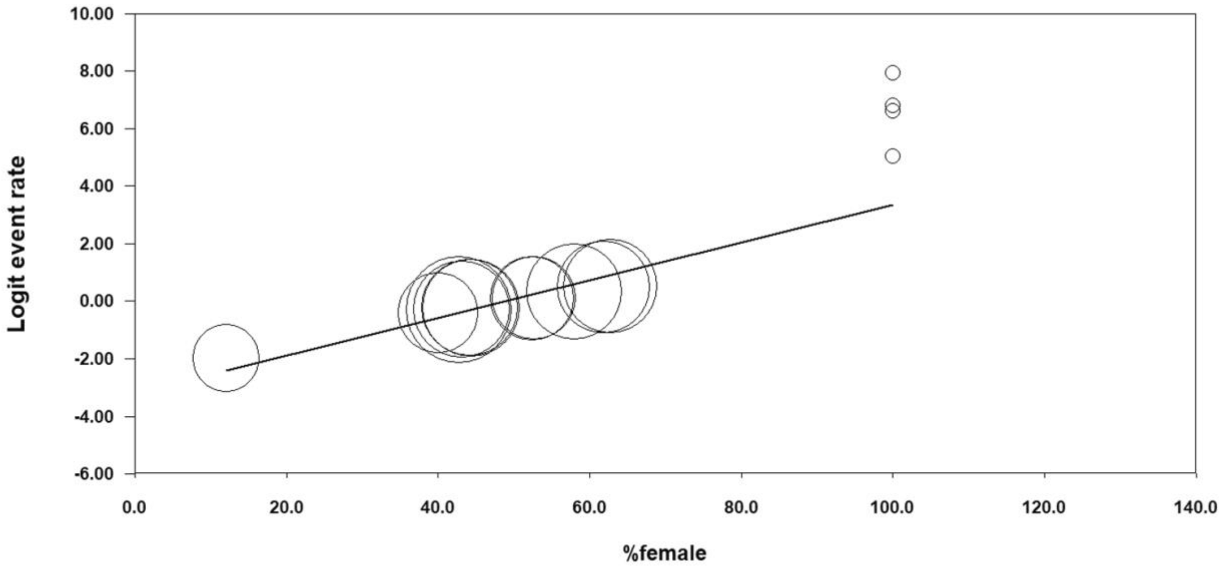

Meta-regression scatter plot showing the relationship between Female and logit event rate across studies. The fitted regression line indicates statistically significant positive association between Female and logit event rate ($\beta = 6.6$, 95% CI= 5.4 to 7.7, $p < 0.001$, n =7,987), suggesting that %Female significantly explains between-study variation in event rates.

Appendix 16. Pooled Prevalence of Underweight among Migrant Workers based on Study design

	Study name	Event rate	Lower limit	Upper limit	Z-Value	p-Value
(a)	Leong (2006)	0.079	0.036	0.165	-5.775	0.000
		0.079	0.036	0.165	-5.775	0.000
		0.079	0.036	0.165	-5.775	0.000
(b)	Alhadlaq et al. (2023)a	0.009	0.006	0.012	-25.874	0.000
	Alhadlaq et al. (2023)b	0.008	0.005	0.011	-26.124	0.000
	Ali et al. (2022)	0.022	0.018	0.028	-32.859	0.000
	Arslan et al. (2023)	0.073	0.053	0.101	-14.027	0.000
	Aung et al. (2019)	0.036	0.022	0.059	-12.475	0.000
	Bhandari et al. (2023)	0.043	0.019	0.091	-7.462	0.000
	Carioca et al. (2017)a	0.140	0.108	0.181	-11.760	0.000
	Carioca et al. (2017)b	0.084	0.058	0.122	-11.402	0.000
	Carioca et al. (2021)a	0.069	0.038	0.123	-7.942	0.000
	Carioca et al. (2021)b	0.020	0.007	0.061	-6.649	0.000
	Charoensook et al. (2018)	0.081	0.060	0.110	-14.321	0.000
	Dah Poe et al. (2022)	0.072	0.051	0.102	-13.250	0.000
	Hall et al. (2021)	0.017	0.011	0.025	-19.420	0.000
	Huang et al. (2020)a	0.017	0.011	0.025	-19.420	0.000
	Huang et al. (2020)b	0.038	0.023	0.063	-11.865	0.000
	Joy et al. (2017)	0.117	0.110	0.125	-53.443	0.000
	Kuhn et al. (2020)a	0.112	0.096	0.131	-23.180	0.000
	Kuhn et al. (2020)b	0.022	0.013	0.034	-15.568	0.000
	Mei et al. (2020)	0.032	0.012	0.082	-6.709	0.000
	Sanskriti et al. (2024)	0.208	0.139	0.301	-5.312	0.000
	Sornlorm and Thi (2024)	0.116	0.088	0.151	-13.107	0.000
		0.082	0.078	0.086	-86.813	0.000
		0.046	0.030	0.069	-13.958	0.000
		0.082	0.078	0.086	-87.005	0.000
		0.051	0.036	0.073	-15.057	0.000

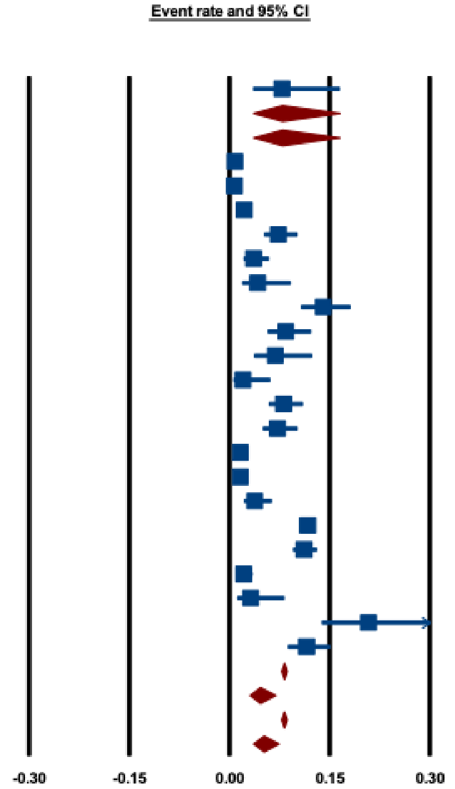

This figure illustrates the pooled prevalence of underweight among migrant workers based on study design; (a) Cohort study was 7.9% (95% CI= 3.6 to 16.5; n= 76) and (b) Cross-sectional study setting was 4.6% (95% CI= 3.0 to 6.9; n= 26,128)

Appendix 17. The Pooled Prevalence of Underweight among Migrant Workers by Publish Year of Studies

	Study name	Statistics for each study				
		Event rate	Lower limit	Upper limit	Z-Value	p-Value
(a)	Aung et al. (2019)	0.036	0.022	0.059	-12.475	0.000
	Carioca et al. (2017)a	0.140	0.108	0.181	-11.760	0.000
	Carioca et al. (2017)b	0.084	0.058	0.122	-11.402	0.000
	Charoensook et al. (2018)	0.081	0.060	0.110	-14.321	0.000
	Huang et al. (2020)a	0.017	0.011	0.025	-19.420	0.000
	Huang et al. (2020)b	0.038	0.023	0.063	-11.865	0.000
	Joy et al. (2017)	0.117	0.110	0.125	-53.443	0.000
	Kuhn et al. (2020)a	0.112	0.096	0.131	-23.180	0.000
	Kuhn et al. (2020)b	0.022	0.013	0.034	-15.568	0.000
	Mei et al. (2020)	0.032	0.012	0.082	-6.709	0.000
		0.104	0.098	0.110	-68.078	0.000
		0.057	0.039	0.084	-13.459	0.000
(b)	Alhadlaq et al. (2023)a	0.009	0.006	0.012	-25.874	0.000
	Alhadlaq et al. (2023)b	0.008	0.005	0.011	-26.124	0.000
	Ali et al. (2022)	0.022	0.018	0.028	-32.859	0.000
	Arslan et al. (2023)	0.073	0.053	0.101	-14.027	0.000
	Bhandari et al. (2023)	0.043	0.019	0.091	-7.462	0.000
	Carioca et al. (2021)a	0.069	0.038	0.123	-7.942	0.000
	Carioca et al. (2021)b	0.020	0.007	0.061	-6.649	0.000
	Dah Poe et al. (2022)	0.072	0.051	0.102	-13.250	0.000
	Hall et al. (2021)	0.017	0.011	0.025	-19.420	0.000
	Sanskriti et al. (2024)	0.208	0.139	0.301	-5.312	0.000
	Sornlorm and Thi (2024)	0.116	0.088	0.151	-13.107	0.000
		0.034	0.031	0.039	-56.687	0.000
		0.038	0.020	0.073	-9.161	0.000
		0.082	0.078	0.086	-86.813	0.000
		0.052	0.037	0.072	-16.248	0.000

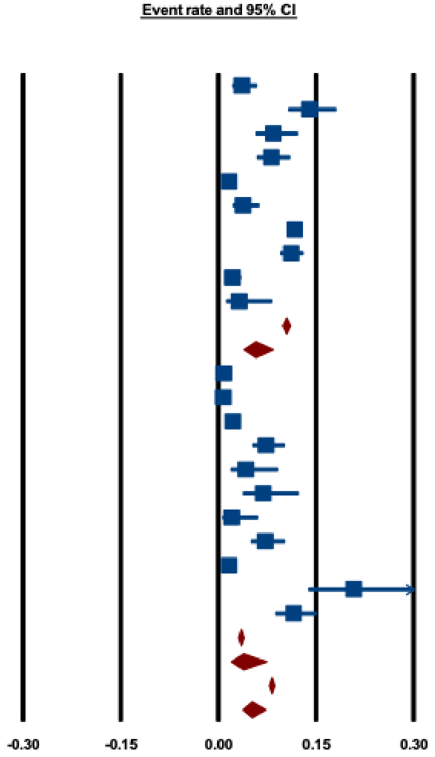

This figure illustrates the pooled prevalence of underweight among migrant workers by publish year of studies; (a) In years 2016 - 2020 was 5.7% (95% CI, 3.9 to 8.4; n = 12,233) and (b) In years 2021 - 2025 was 3.8% (95% CI, 2.0 to 7.3; n = 15,093)

Appendix 18. Pooled Prevalence of Underweight among Migrant Workers according to Study Settings

	Study name	Event rate	Lower limit	Upper limit	Z-Value	p-Value
		Statistics for each study				
(a)	Arslan et al. (2023)	0.073	0.053	0.101	-14.027	0.000
	Aung et al. (2019)	0.036	0.022	0.059	-12.475	0.000
	Carioca et al. (2017)a	0.140	0.108	0.181	-11.760	0.000
	Carioca et al. (2017)b	0.084	0.058	0.122	-11.402	0.000
	Carioca et al. (2021)a	0.069	0.038	0.123	-7.942	0.000
	Carioca et al. (2021)b	0.020	0.007	0.061	-6.649	0.000
	Charoensook et al. (2018)	0.081	0.060	0.110	-14.321	0.000
	Dah Poe et al. (2022)	0.072	0.051	0.102	-13.250	0.000
	Hall et al. (2021)	0.017	0.011	0.025	-19.420	0.000
	Huang et al. (2020)a	0.017	0.011	0.025	-19.420	0.000
	Huang et al. (2020)b	0.038	0.023	0.063	-11.865	0.000
	Joy et al. (2017)	0.117	0.110	0.125	-53.443	0.000
	Kuhn et al. (2020)a	0.112	0.096	0.131	-23.180	0.000
	Kuhn et al. (2020)b	0.022	0.013	0.034	-15.568	0.000
	Mei et al. (2020)	0.032	0.012	0.082	-6.709	0.000
	Sanskriti et al. (2024)	0.208	0.139	0.301	-5.312	0.000
	Sornlorm and Thi (2024)	0.116	0.088	0.151	-13.107	0.000
		0.099	0.094	0.105	-74.696	0.000
		0.060	0.044	0.082	-16.375	0.000
(b)	Alhadlaq et al. (2023)a	0.009	0.006	0.012	-25.874	0.000
	Alhadlaq et al. (2023)b	0.008	0.005	0.011	-26.124	0.000
	Ali et al. (2022)	0.022	0.018	0.028	-32.859	0.000
	Bhandari et al. (2023)	0.043	0.019	0.091	-7.462	0.000
	Leong (2006)	0.079	0.036	0.165	-5.775	0.000
		0.016	0.014	0.019	-49.633	0.000
		0.020	0.010	0.040	-10.781	0.000
		0.082	0.078	0.086	-87.005	0.000
		0.050	0.038	0.066	-19.396	0.000

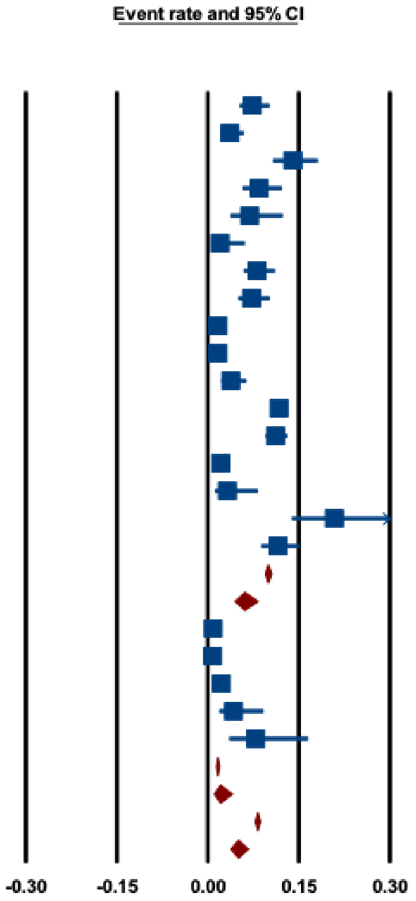

This figure illustrates the pooled prevalence of underweight among migrant workers according to migrant setting; (a) Community setting was 6.0% (95% CI, 4.4 to 8.2; n = 15,404) and (b) Hospital setting was 2.0% (95% CI, 1.0 to 4.0; n = 10,976)

Appendix 19. Pooled Prevalence of Underweight among Migrant Workers based on Sample size category

	Study name	Event rate	Lower limit	Upper limit	Z-Value	p-Value
(a)	Arslan et al. (2023)	0.073	0.053	0.101	-14.027	0.000
	Aung et al. (2019)	0.036	0.022	0.059	-12.475	0.000
	Bhandari et al. (2023)	0.043	0.019	0.091	-7.462	0.000
	Carioca et al. (2017)a	0.140	0.108	0.181	-11.760	0.000
	Carioca et al. (2017)b	0.084	0.058	0.122	-11.402	0.000
	Carioca et al. (2021)a	0.069	0.038	0.123	-7.942	0.000
	Carioca et al. (2021)b	0.020	0.007	0.061	-6.649	0.000
	Charoensook et al. (2018)	0.081	0.060	0.110	-14.321	0.000
	Dah Poe et al. (2022)	0.072	0.051	0.102	-13.250	0.000
	Huang et al. (2020)b	0.038	0.023	0.063	-11.865	0.000
	Kuhn et al. (2020)b	0.022	0.013	0.034	-15.568	0.000
	Leong (2006)	0.079	0.036	0.165	-5.775	0.000
	Mei et al. (2020)	0.032	0.012	0.082	-6.709	0.000
	Sanskriti et al. (2024)	0.208	0.139	0.301	-5.312	0.000
	Sornlorm and Thi (2024)	0.116	0.088	0.151	-13.107	0.000
		0.080	0.071	0.088	-41.466	0.000
		0.065	0.048	0.089	-15.473	0.000
(b)	Alhadlaq et al. (2023)a	0.009	0.006	0.012	-25.874	0.000
	Alhadlaq et al. (2023)b	0.008	0.005	0.011	-26.124	0.000
	Ali et al. (2022)	0.022	0.018	0.028	-32.859	0.000
	Hall et al. (2021)	0.017	0.011	0.025	-19.420	0.000
	Huang et al. (2020)a	0.017	0.011	0.025	-19.420	0.000
	Joy et al. (2017)	0.117	0.110	0.125	-53.443	0.000
	Kuhn et al. (2020)a	0.112	0.096	0.131	-23.180	0.000
		0.082	0.078	0.087	-76.490	0.000
		0.025	0.010	0.060	-7.916	0.000
		0.082	0.078	0.086	-87.005	0.000
		0.058	0.043	0.078	-17.263	0.000

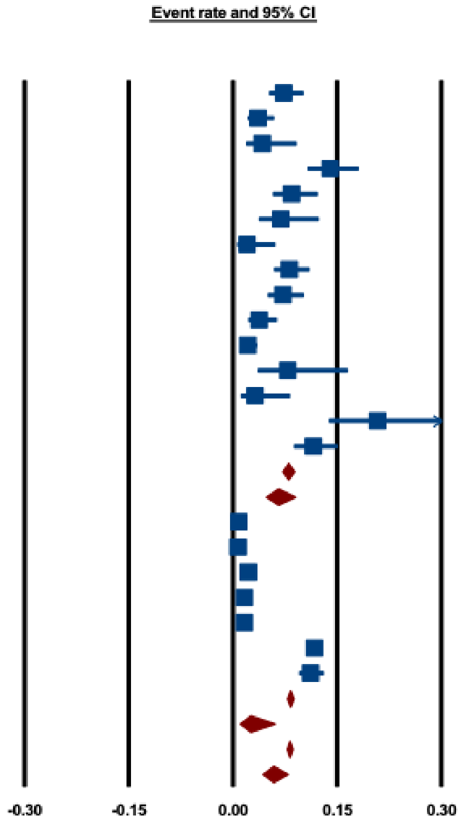

This figure illustrates the pooled prevalence of underweight among migrant workers based on sample size category; (a) Study with sample less than 1000 was 44.7% (95% CI, 37.4 to 52.3; n = 11,651) and (b) Study with sample more than 1000 was 41.5% (95% CI, 35.8 to 47.5; n = 14,593)

Appendix 20. Pooled Prevalence of Underweight among Migrant Workers according to Study Quality

	Study name	Event rate	Lower limit	Upper limit	Z-Value	p-Value
(a)	Alhadlaq et al. (2023)a	0.009	0.006	0.012	-25.874	0.000
	Alhadlaq et al. (2023)b	0.008	0.005	0.011	-26.124	0.000
	Ali et al. (2022)	0.022	0.018	0.028	-32.859	0.000
	Aung et al. (2019)	0.036	0.022	0.059	-12.475	0.000
	Bhandari et al. (2023)	0.043	0.019	0.091	-7.462	0.000
	Carioca et al. (2017)a	0.140	0.108	0.181	-11.760	0.000
	Carioca et al. (2017)b	0.084	0.058	0.122	-11.402	0.000
	Carioca et al. (2021)a	0.069	0.038	0.123	-7.942	0.000
	Carioca et al. (2021)b	0.020	0.007	0.061	-6.649	0.000
	Charoensook et al. (2018)	0.081	0.060	0.110	-14.321	0.000
	Dah Poe et al. (2022)	0.072	0.051	0.102	-13.250	0.000
	Huang et al. (2020)a	0.017	0.011	0.025	-19.420	0.000
	Huang et al. (2020)b	0.038	0.023	0.063	-11.865	0.000
	Kuhn et al. (2020)a	0.112	0.096	0.131	-23.180	0.000
	Kuhn et al. (2020)b	0.022	0.013	0.034	-15.568	0.000
	Mei et al. (2020)	0.032	0.012	0.082	-6.709	0.000
	Sornlorm and Thi (2024)	0.116	0.088	0.151	-13.107	0.000
		0.051	0.047	0.056	-66.357	0.000
		0.040	0.025	0.065	-12.162	0.000
(b)	Leong (2006)	0.079	0.036	0.165	-5.775	0.000
		0.079	0.036	0.165	-5.775	0.000
		0.079	0.036	0.165	-5.775	0.000
(c)	Arslan et al. (2023)	0.073	0.053	0.101	-14.027	0.000
	Hall et al. (2021)	0.017	0.011	0.025	-19.420	0.000
	Joy et al. (2017)	0.117	0.110	0.125	-53.443	0.000
	Sanskriti et al. (2024)	0.208	0.139	0.301	-5.312	0.000
		0.111	0.104	0.118	-57.875	0.000
		0.076	0.032	0.169	-5.407	0.000
		0.082	0.078	0.086	-87.005	0.000
		0.053	0.036	0.076	-14.407	0.000

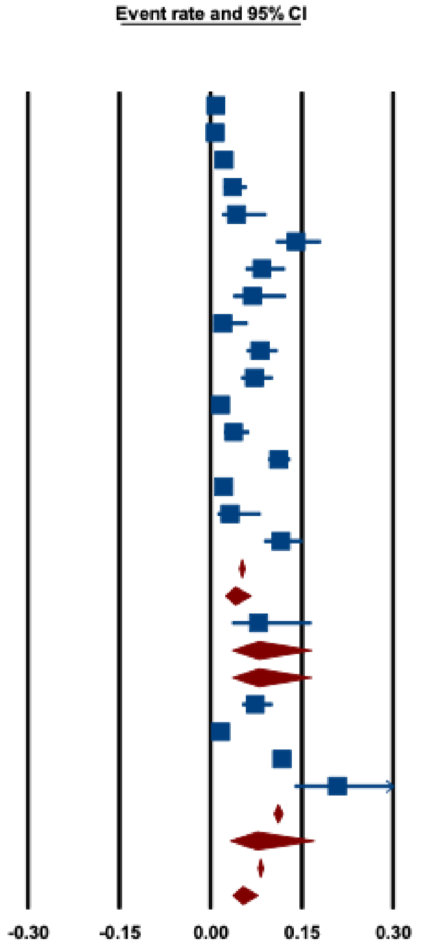

This figure illustrates the pooled prevalence of underweight among migrant workers according to study quality; (a) High quality study was 4.0% (95% CI, 2.5 to 6.5; n = 16,407), (b) Low quality study was 7.9% (95% CI, 3.6 to 16.5; n = 76) and (c) Moderate quality study was 7.6% (95% CI, 3.2 to 16.9; n = 9,761).

Appendix 21. Pooled Prevalence of Underweight among Migrant Workers based on Destination Continent

	Study name	Event rate	Lower limit	Upper limit	Z-Value	p-Value
(a)	Aung et al. (2019)	0.036	0.022	0.059	-12.475	0.000
	Bhandari et al. (2023)	0.043	0.019	0.091	-7.462	0.000
	Charoensook et al. (2018)	0.081	0.060	0.110	-14.321	0.000
	Dah Poe et al. (2022)	0.072	0.051	0.102	-13.250	0.000
	Hall et al. (2021)	0.017	0.011	0.025	-19.420	0.000
	Huang et al. (2020)a	0.017	0.011	0.025	-19.420	0.000
	Huang et al. (2020)b	0.038	0.023	0.063	-11.865	0.000
	Leong (2006)	0.079	0.036	0.165	-5.775	0.000
	Mei et al. (2020)	0.032	0.012	0.082	-6.709	0.000
	Somlorm and Thi (2024)	0.116	0.088	0.151	-13.107	0.000
		0.052	0.045	0.059	-40.318	0.000
		0.045	0.027	0.073	-11.498	0.000
(b)	Arslan et al. (2023)	0.073	0.053	0.101	-14.027	0.000
		0.073	0.053	0.101	-14.027	0.000
		0.073	0.053	0.101	-14.027	0.000
(c)	Carioca et al. (2017)a	0.140	0.108	0.181	-11.760	0.000
	Carioca et al. (2017)b	0.084	0.058	0.122	-11.402	0.000
	Carioca et al. (2021)a	0.069	0.038	0.123	-7.942	0.000
	Carioca et al. (2021)b	0.020	0.007	0.061	-6.649	0.000
		0.104	0.085	0.127	-18.943	0.000
		0.076	0.043	0.133	-7.921	0.000
(d)	Alhadlaq et al. (2023)a	0.009	0.006	0.012	-25.874	0.000
	Alhadlaq et al. (2023)b	0.008	0.005	0.011	-26.124	0.000
	Ali et al. (2022)	0.022	0.018	0.028	-32.859	0.000
	Kuhn et al. (2020)b	0.022	0.013	0.034	-15.568	0.000
		0.015	0.013	0.018	-51.352	0.000
		0.013	0.007	0.024	-14.038	0.000
(e)	Joy et al. (2017)	0.117	0.110	0.125	-53.443	0.000
	Kuhn et al. (2020)a	0.112	0.096	0.131	-23.180	0.000
	Sanskriti et al. (2024)	0.208	0.139	0.301	-5.312	0.000
		0.118	0.111	0.125	-58.430	0.000
		0.125	0.104	0.151	-17.643	0.000
		0.082	0.078	0.086	-87.005	0.000
		0.085	0.073	0.098	-28.939	0.000

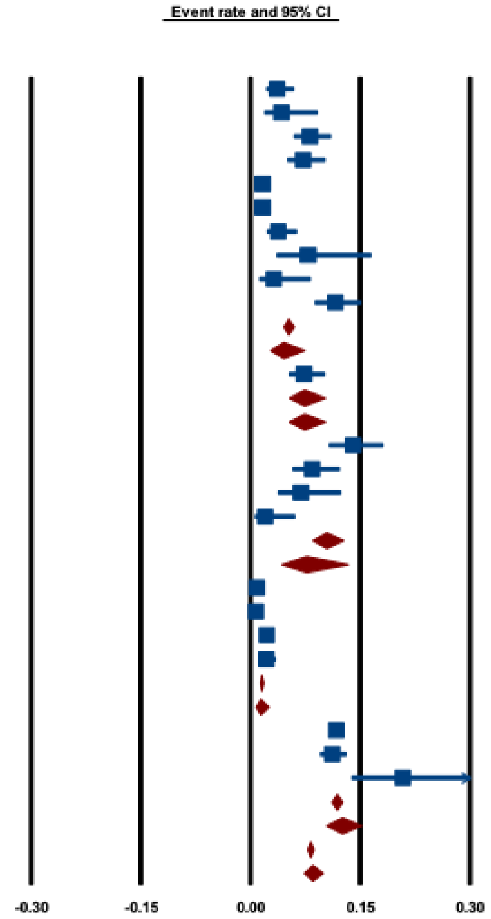

This figure illustrates the pooled prevalence of underweight among migrant workers based on destination continent; (a) East Asia and Pacific was 4.5% (95% CI, 2.7 to 7.3; n = 11,483), (b) Europe & Central Asia was 7.3% (95% CI, 5.3 to 10.1; n = 450), (c) Latin America & Caribbean was 7.6% (95% CI, 4.3 to 13.3; n = 938), (d) Midle East & North Africa was 1.3% (95% CI, 0.7 to 2.4; n = 11,549) and (e) South Asia was 12.5% (95% CI, 10.4 to 15.1; n = 8,131).

Appendix 22. Pooled Prevalence of Underweight among Migrant Workers based on income level of destination country

	Study name	Event rate	Lower limit	Upper limit	Z-Value	p-Value
		Statistics for each study				
(a)	Alhadlaq et al. (2023)a	0.009	0.006	0.012	-25.874	0.000
	Alhadlaq et al. (2023)b	0.008	0.005	0.011	-26.124	0.000
	Ali et al. (2022)	0.022	0.018	0.028	-32.859	0.000
	Bhandari et al. (2023)	0.043	0.019	0.091	-7.462	0.000
	Hall et al. (2021)	0.017	0.011	0.025	-19.420	0.000
	Huang et al. (2020)a	0.017	0.011	0.025	-19.420	0.000
	Huang et al. (2020)b	0.038	0.023	0.063	-11.865	0.000
	Kuhn et al. (2020)b	0.022	0.013	0.034	-15.568	0.000
		0.017	0.015	0.019	-59.758	0.000
		0.018	0.012	0.026	-19.947	0.000
(b)	Joy et al. (2017)	0.117	0.110	0.125	-53.443	0.000
	Kuhn et al. (2020)a	0.112	0.096	0.131	-23.180	0.000
	Sanskriti et al. (2024)	0.208	0.139	0.301	-5.312	0.000
		0.118	0.111	0.125	-58.430	0.000
		0.125	0.104	0.151	-17.643	0.000
(c)	Arslan et al. (2023)	0.073	0.053	0.101	-14.027	0.000
	Aung et al. (2019)	0.036	0.022	0.059	-12.475	0.000
	Carioca et al. (2017)a	0.140	0.108	0.181	-11.760	0.000
	Carioca et al. (2017)b	0.084	0.058	0.122	-11.402	0.000
	Carioca et al. (2021)a	0.069	0.038	0.123	-7.942	0.000
	Carioca et al. (2021)b	0.020	0.007	0.061	-6.649	0.000
	Charoensook et al. (2018)	0.081	0.060	0.110	-14.321	0.000
	Dah Poe et al. (2022)	0.072	0.051	0.102	-13.250	0.000
	Leong (2006)	0.079	0.036	0.165	-5.775	0.000
	Mei et al. (2020)	0.032	0.012	0.082	-6.709	0.000
	Somlorm and Thi (2024)	0.116	0.088	0.151	-13.107	0.000
		0.086	0.076	0.096	-36.272	0.000
		0.073	0.056	0.095	-17.283	0.000
		0.082	0.078	0.086	-87.005	0.000
		0.079	0.068	0.091	-30.429	0.000

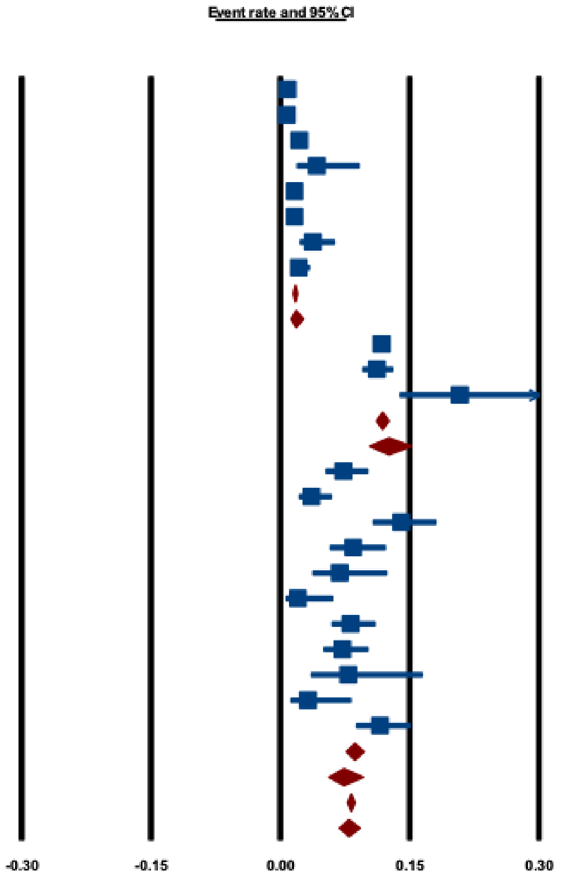

This figure illustrates the pooled prevalence of underweight among migrant workers based on income level on destination country; (a) High income country was 1.8% (95% CI, 1.2 to 2.6; n = 14,721), (b) Lower middle income country was 12.5% (95% CI, 10.4 to 15.1; n = 8,131) and (c) Upper middle income country was 7.3% (95% CI, 5.6 to 9.5; n = 3,392)

Appendix 23. Pooled Prevalence of Underweight among Migrant Workers according by Marital status

	Study name	Event rate	Lower limit	Upper limit	Z-Value	p-Value
(a)	Aung et al. (2019)1	0.027	0.015	0.047	-11.784	0.000
	Bhandari et al. (2023)1	0.021	0.007	0.064	-6.560	0.000
	Charoensook et al. (2018)1	0.066	0.047	0.093	-14.222	0.000
	Huang et al. (2020)a1	0.007	0.004	0.013	-15.521	0.000
	Huang et al. (2020)b1	0.014	0.006	0.032	-9.522	0.000
	Joy et al. (2017)1	0.103	0.096	0.110	-54.148	0.000
	Mei et al. (2020)1	0.016	0.004	0.062	-5.778	0.000
	Poe et al. (2022)1	0.057	0.038	0.085	-13.048	0.000
	Sornlorm et al. (2024)1	0.079	0.056	0.109	-13.348	0.000
		0.035	0.020	0.060	-11.279	0.000
(b)	Aung et al. (2019)	0.010	0.004	0.025	-9.215	0.000
	Bhandari et al. (2023)	0.021	0.007	0.064	-6.560	0.000
	Charoensook et al. (2018)	0.015	0.007	0.031	-10.990	0.000
	Huang et al. (2020)a	0.009	0.005	0.016	-16.728	0.000
	Huang et al. (2020)b	0.024	0.013	0.046	-10.931	0.000
	?Joy et al. (2017)	0.014	0.012	0.018	-41.487	0.000
	Mei et al. (2020)	0.008	0.001	0.055	-4.801	0.000
	Poe et al. (2022)	0.015	0.007	0.033	-10.186	0.000
	Sornlorm et al. (2024)	0.037	0.022	0.060	-12.393	0.000
		0.016	0.012	0.023	-24.197	0.000
(c)	Huang et al. (2020)a2	0.005	0.002	0.011	-13.947	0.000
	Huang et al. (2020)b2	0.016	0.007	0.036	-9.967	0.000
	Mei et al. (2020)2	0.004	0.000	0.060	-3.899	0.000
		0.008	0.003	0.021	-9.694	0.000
		0.018	0.014	0.024	-28.261	0.000

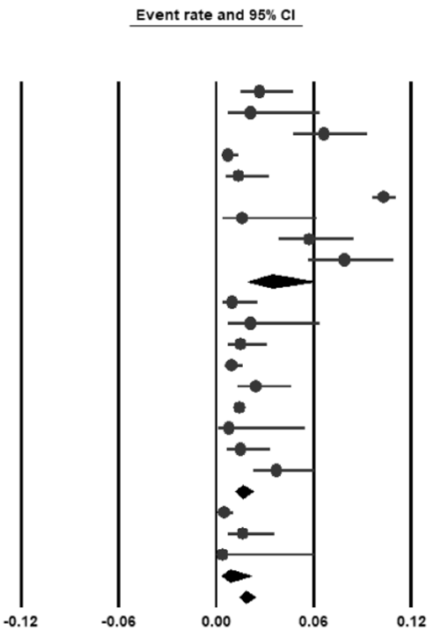

This figure illustrates the pooled prevalence of underweight among migrant workers according by marital status; (a) Married was 65.5% (95% CI, 49.1 to 78.9; n = 5332), (b) Single was 25.1% (95% CI, 19.1 to 32.4; n = 5332) and (c) Widow or widower was 24.4% (95% CI, 16.8 to 34.0; n = 2296).

Appendix 24. Pooled Prevalence of Underweight among Migrant Workers by Education Level

Study name	Event rate	Lower limit	Upper limit	Z-Value	p-Value
(a) Arslan et al. (2023)	0.240	0.203	0.282	-10.443	0.000
Aung et al. (2019)	0.763	0.720	0.802	10.126	0.000
Bhandari et al. (2023)	0.149	0.099	0.218	-7.369	0.000
Carioca et al. (2017)a	0.688	0.637	0.734	6.834	0.000
Carioca et al. (2017)b	0.841	0.795	0.879	10.484	0.000
Carioca et al. (2021)a	0.476	0.396	0.557	-0.581	0.561
Carioca et al. (2021)b	0.655	0.575	0.727	3.717	0.000
Dah Poe et al. (2022)	0.346	0.301	0.394	-6.081	0.000
Hall et al. (2021)	0.018	0.012	0.027	-19.812	0.000
Huang et al. (2020)a	0.018	0.012	0.027	-19.812	0.000
Joy et al. (2017)	0.248	0.238	0.258	-39.476	0.000
Somlorm and Thi (2024)	0.261	0.221	0.306	-9.207	0.000
	0.292	0.282	0.301	-38.085	0.000
(b) Arslan et al. (2023)1	0.760	0.718	0.797	10.443	0.000
Aung et al. (2019)1	0.237	0.198	0.280	-10.126	0.000
Bhandari et al. (2023)1	0.851	0.782	0.901	7.369	0.000
Carioca et al. (2017)a1	0.312	0.266	0.363	-6.834	0.000
Carioca et al. (2017)b1	0.159	0.121	0.205	-10.484	0.000
Carioca et al. (2021)a1	0.524	0.443	0.604	0.581	0.561
Carioca et al. (2021)b1	0.345	0.273	0.425	-3.717	0.000
Dah Poe et al. (2022)1	0.654	0.606	0.699	6.081	0.000
Hall et al. (2021)1	0.982	0.973	0.988	19.812	0.000
Huang et al. (2020)a1	0.982	0.973	0.988	19.812	0.000
Joy et al. (2017)1	0.752	0.742	0.762	39.476	0.000
Somlorm and Thi (2024)1	0.983	0.964	0.992	10.604	0.000
	0.710	0.700	0.719	37.571	0.000
	0.496	0.488	0.504	-0.894	0.372

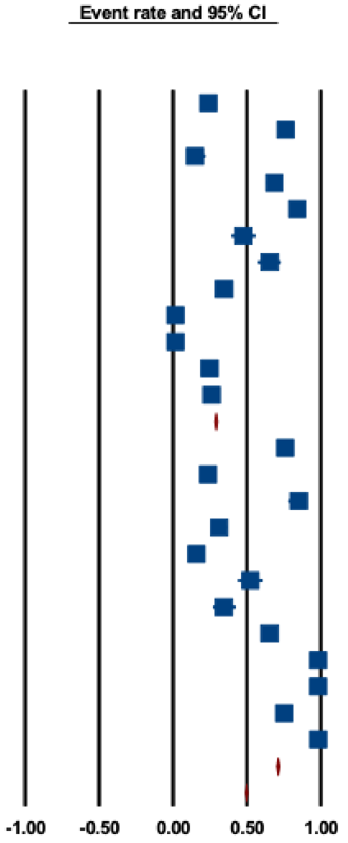

This figure illustrates the pooled prevalence of underweight among migrant workers by education level; (a) Education level that lower than secondary level was 31.3% (95% CI, 18.5 to 47.9; n = 7,697) and (b) Education level that more than secondary level was 73.5% (95% CI, 56.4 to 85.6; n = 7,697).

Appendix 25. Pooled Prevalence of Underweight among Migrant Workers according to Migrant Setting

	Study name	Event rate	Lower limit	Upper limit	Z-Value	p-Value
(a)	Alhadlaq et al. (2023)a	0.009	0.006	0.012	-25.874	0.000
	Alhadlaq et al. (2023)b	0.008	0.005	0.011	-26.124	0.000
		0.008	0.006	0.010	-36.765	0.000
		0.008	0.006	0.010	-36.765	0.000
(b)	Ali et al. (2022)	0.022	0.018	0.028	-32.859	0.000
	Arslan et al. (2023)	0.073	0.053	0.101	-14.027	0.000
	Aung et al. (2019)	0.036	0.022	0.059	-12.475	0.000
	Bhandari et al. (2023)	0.043	0.019	0.091	-7.462	0.000
	Carioca et al. (2017)a	0.140	0.108	0.181	-11.760	0.000
	Carioca et al. (2017)b	0.084	0.058	0.122	-11.402	0.000
	Carioca et al. (2021)a	0.069	0.038	0.123	-7.942	0.000
	Carioca et al. (2021)b	0.020	0.007	0.061	-6.649	0.000
	Charoensook et al. (2018)	0.081	0.060	0.110	-14.321	0.000
	Dah Poe et al. (2022)	0.072	0.051	0.102	-13.250	0.000
	Hall et al. (2021)	0.017	0.011	0.025	-19.420	0.000
	Huang et al. (2020)a	0.017	0.011	0.025	-19.420	0.000
	Huang et al. (2020)b	0.038	0.023	0.063	-11.865	0.000
	Joy et al. (2017)	0.117	0.110	0.125	-53.443	0.000
	Kuhn et al. (2020)a	0.112	0.096	0.131	-23.180	0.000
	Kuhn et al. (2020)b	0.022	0.013	0.034	-15.568	0.000
	Leong (2006)	0.079	0.036	0.165	-5.775	0.000
	Sanskriti et al. (2024)	0.208	0.139	0.301	-5.312	0.000
	Sornlorm and Thi (2024)	0.116	0.088	0.151	-13.107	0.000
		0.091	0.086	0.096	-80.790	0.000
		0.058	0.041	0.081	-15.214	0.000
		0.082	0.078	0.086	-86.768	0.000
		0.016	0.013	0.019	-38.770	0.000

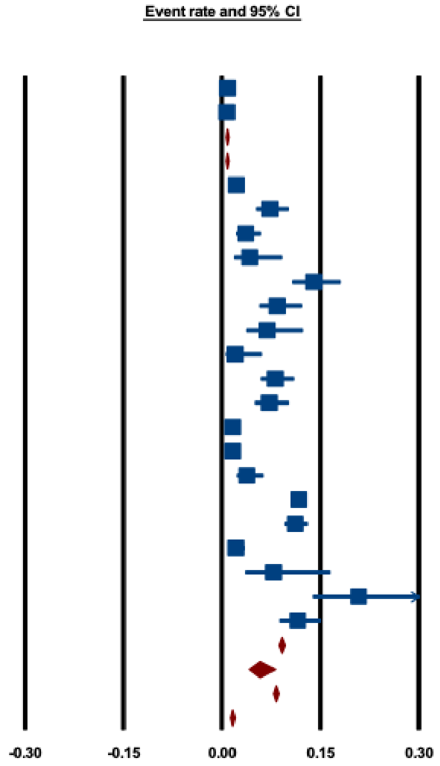

This figure illustrates the pooled prevalence of underweight among migrant workers according to migrant setting; (a) Rural setting was 0.8% (95% CI, 0.6 to 1.0; n = 7,285) and (b) Urban setting was 5.8% (95% CI, 4.1 to 8.1; n = 15,485)

Appendix 26. Pooled Prevalence of Underweight among Migrant Workers according to Migrant Occupational

	Study name	Event rate	Lower limit	Upper limit	Z-Value	p-Value
(a)	Aung et al. (2019)	0.036	0.022	0.059	-12.475	0.000
	Bhandari et al. (2023)	0.043	0.019	0.091	-7.462	0.000
	Carioca et al. (2021)b	0.020	0.007	0.061	-6.649	0.000
	Charoensook et al. (2018)	0.081	0.060	0.110	-14.321	0.000
	Dah Poe et al. (2022)	0.072	0.051	0.102	-13.250	0.000
	Hall et al. (2021)	0.017	0.011	0.025	-19.420	0.000
	Huang et al. (2020)a	0.017	0.011	0.025	-19.420	0.000
	Huang et al. (2020)b	0.038	0.023	0.063	-11.865	0.000
	Joy et al. (2017)	0.117	0.110	0.125	-53.443	0.000
	Leong (2006)	0.079	0.036	0.165	-5.775	0.000
	Mei et al. (2020)	0.032	0.012	0.082	-6.709	0.000
	Sornlorm and Thi (2024)	0.116	0.088	0.151	-13.107	0.000
		0.099	0.093	0.104	-66.310	0.000
		0.047	0.029	0.075	-11.920	0.000
(b)	Alhadlaq et al. (2023)a	0.009	0.006	0.012	-25.874	0.000
	Alhadlaq et al. (2023)b	0.008	0.005	0.011	-26.124	0.000
		0.008	0.006	0.010	-36.765	0.000
		0.008	0.006	0.010	-36.765	0.000
		0.085	0.080	0.090	-73.345	0.000
		0.012	0.009	0.015	-38.133	0.000

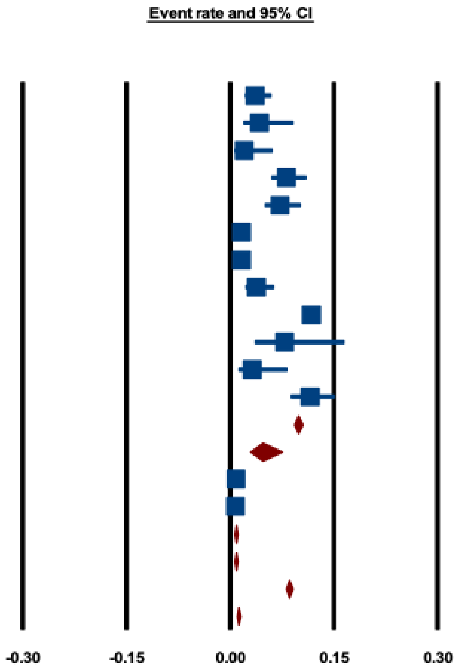

This figure illustrates the pooled prevalence of underweight among migrant workers according to migrant setting; (a) Blue-collar type was 4.7% (95% CI, 2.9 to 7.5; n = 13,070) and (b) White-collar type was 0.8% (95% CI, 0.6 to 1.0; n = 7,285)

Appendix 27. Pooled Prevalence of Underweight among Migrant Workers according to Migrant Duration

Study name	Event rate	Lower limit	Upper limit	Z-Value	p-Value
	**Statistics for each study**				
(a) Alhadlaq et al. (2023)a	0.009	0.006	0.012	-25.874	0.000
Alhadlaq et al. (2023)b	0.008	0.005	0.011	-26.124	0.000
Aung et al. (2019)	0.036	0.022	0.059	-12.475	0.000
Bhandari et al. (2023)	0.043	0.019	0.091	-7.462	0.000
Charoensook et al. (2018)	0.081	0.060	0.110	-14.321	0.000
Hall et al. (2021)	0.017	0.011	0.025	-19.420	0.000
Huang et al. (2020)a	0.017	0.011	0.025	-19.420	0.000
Huang et al. (2020)b	0.038	0.023	0.063	-11.865	0.000
Leong (2006)	0.079	0.036	0.165	-5.775	0.000
Mei et al. (2020)	0.032	0.012	0.082	-6.709	0.000
Sornlorm and Thi (2024)	0.116	0.088	0.151	-13.107	0.000
	0.031	0.027	0.035	-51.577	0.000
	0.031	0.016	0.059	-9.909	0.000
	0.031	0.027	0.035	-51.577	0.000
	0.031	0.016	0.059	-9.909	0.000

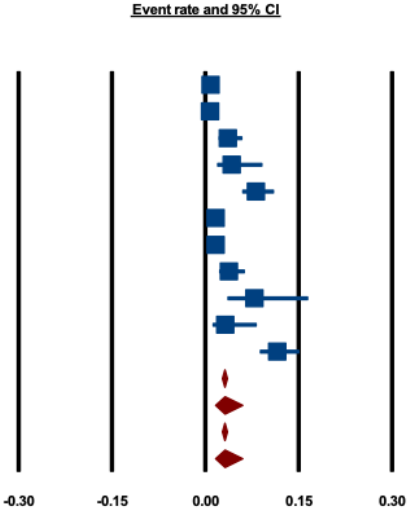

This figure illustrates the pooled prevalence of underweight among migrant workers according to migrant setting; (a) Blue-collar type was 4.7% (95% CI, 2.9 to 7.5; n = 13,070) and (b) White-collar type was 0.8% (95% CI, 0.6 to 1.0; n = 7,285)

Appendix 28. Meta-regression Scatter Plot of Overweight/Obesity among Migrant Workers with Mean Age

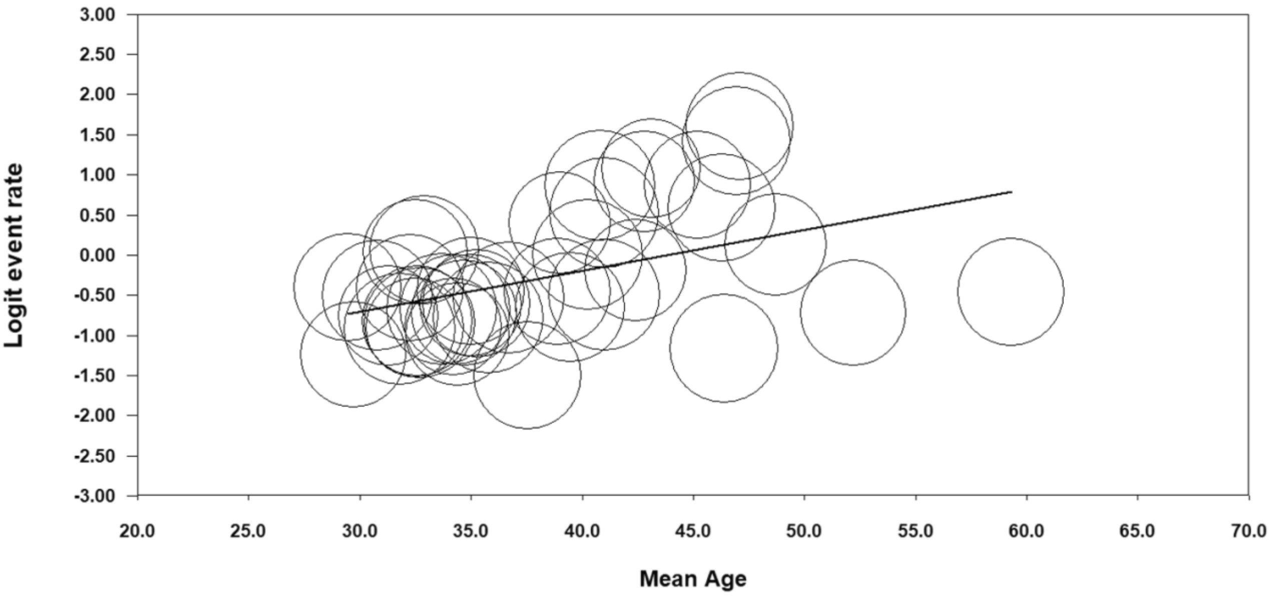

Meta-regression scatter plot showing the relationship between mean age and logit event rate across studies. The fitted regression line indicates a weak positive but significant association between mean age and logit event rate (β = 5.1%, 95% CI: 3.2 to 7.0, p value < 0.001, n = 118,756), suggesting that mean age significantly explains between-study variation in event rates.

Appendix 29. Meta-regression Scatter Plot of Overweight/Obesity among Migrant Workers with Length of Stay

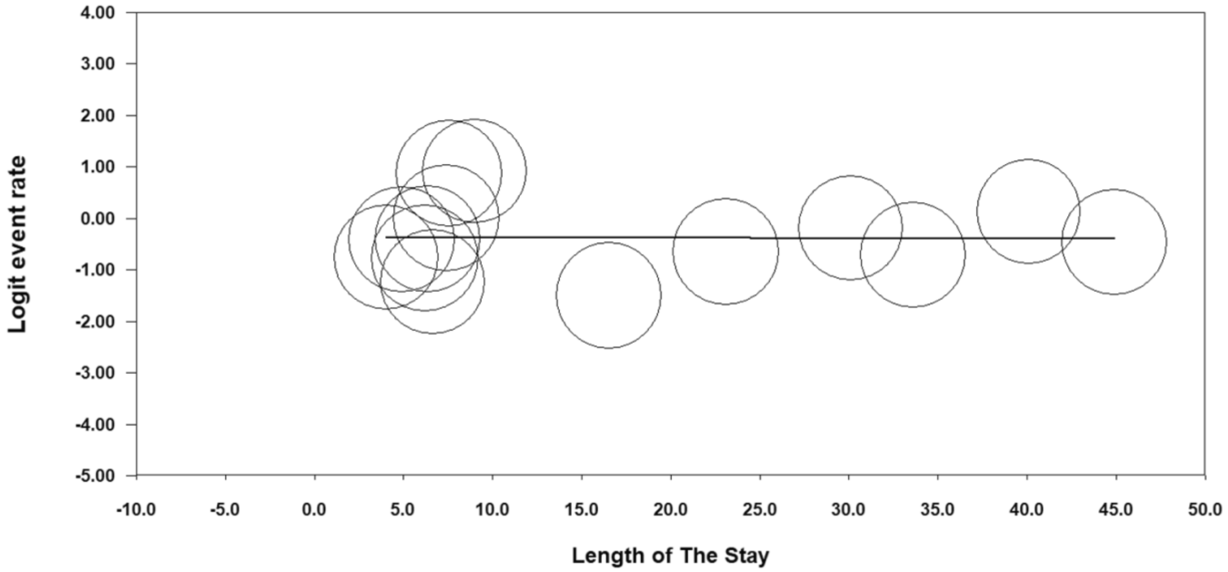

Meta-regression scatter plot showing the relationship between length of stay and logit event rate across studies. The fitted regression line indicates a weak negative but non-significant association between length of stay and logit event rate (β = -0.1%, 95% CI: –3.1 to 2.9, p value = 0.951, n = 60,620), suggesting that length of stay does not significantly explain between-study variation in event rates.

Appendix 30. Meta-regression Scatter Plot of Overweight/Obesity among Migrant Workers with Female

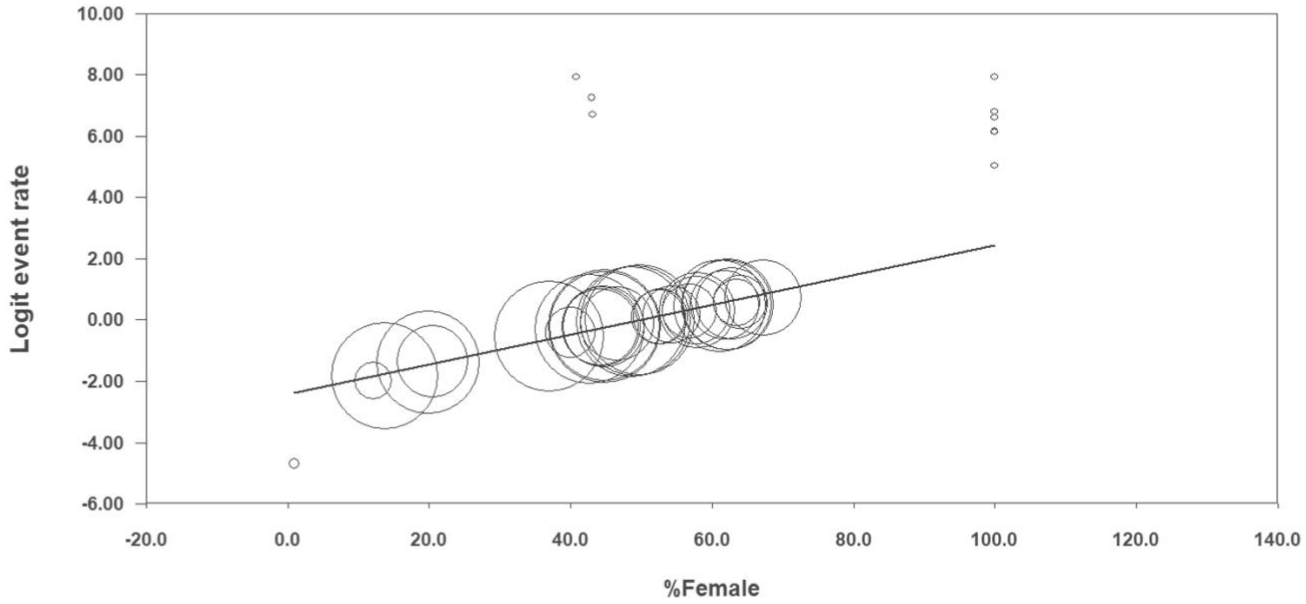

Meta-regression scatter plot showing the relationship between Female and logit event rate across studies. The fitted regression line indicates positive and significant association between Female and logit event rate (β = 4.8%, 95% CI: 4.5 to 5.2, p value <0.001, n = 54,041), suggesting that Female significantly explains between-study variation in event rates.

Appendix 31. The Pooled Prevalence of Overweight/Obesity among Migrant Workers based on Study design

	Study name	Statistics for each study				
		Event rate	Lower limit	Upper limit	Z-Value	p-Value
(a)	Leong (2006)	0.329	0.233	0.442	-2.920	0.003
		0.329	0.233	0.442	-2.920	0.003
		0.329	0.233	0.442	-2.920	0.003
(b)	Abasilim et al. (2024)	0.694	0.602	0.772	3.970	0.000
	Afrifa-Anane et al. (2020)a	0.806	0.780	0.829	17.644	0.000
	Afrifa-Anane et al. (2020)b	0.706	0.665	0.743	9.109	0.000
	Afrifa-Anane et al. (2020)c	0.833	0.807	0.856	17.883	0.000
	Afrifa-Anane et al. (2020)d	0.643	0.599	0.684	6.209	0.000
	Afrifa-Anane et al. (2020)e	0.238	0.212	0.265	-15.468	0.000
	Alhadlaq et al. (2023)a	0.704	0.689	0.719	23.340	0.000
	Alhadlaq et al. (2023)b	0.501	0.485	0.517	0.146	0.884
	Ali et al. (2022)	0.705	0.690	0.720	23.413	0.000
	Arslan et al. (2023)	0.353	0.311	0.399	-6.129	0.000
	Aung et al. (2019)	0.401	0.355	0.449	-4.003	0.000
	Aung et al. (2024)	0.225	0.185	0.271	-9.799	0.000
	Bhandari et al. (2023)	0.319	0.248	0.400	-4.194	0.000
	Bi et al. (2015)a	0.315	0.311	0.319	-79.644	0.000
	Bi et al. (2015)b	0.285	0.275	0.295	-38.066	0.000
	Bi et al. (2015)c	0.336	0.326	0.347	-28.453	0.000
	Bi et al. (2015)d	0.300	0.290	0.310	-35.434	0.000
	Bi et al. (2015)e	0.306	0.296	0.316	-34.221	0.000
	Bi et al. (2015)f	0.370	0.360	0.381	-23.133	0.000
	Bi et al. (2015)g	0.338	0.328	0.349	-28.111	0.000
	Carioca et al. (2017)a	0.387	0.337	0.439	-4.192	0.000
	Carioca et al. (2017)b	0.328	0.277	0.383	-5.803	0.000
	Carioca et al. (2021)a	0.531	0.450	0.611	0.747	0.455
	Carioca et al. (2021)b	0.453	0.374	0.533	-1.149	0.251
	Castaneda et al. (2015)a	0.326	0.274	0.383	-5.710	0.000
	Castaneda et al. (2015)b	0.349	0.282	0.422	-3.942	0.000
	Castaneda et al. (2015)c	0.290	0.212	0.382	-4.208	0.000
	Castaneda et al. (2019)	0.747	0.670	0.811	5.679	0.000
	Charoensook et al. (2018)	0.430	0.386	0.476	-2.998	0.003
	Dah Poe et al. (2022)	0.388	0.342	0.437	-4.450	0.000
	Emiral et al. (2021)	0.389	0.345	0.435	-4.695	0.000
	Hall et al. (2021)	0.628	0.602	0.653	9.447	0.000
	Huang et al. (2020)a	0.628	0.602	0.653	9.447	0.000
	Huang et al. (2020)b	0.545	0.494	0.595	1.716	0.086
	Jorgensen et al. (2011)	0.714	0.640	0.779	5.252	0.000
	Joy et al. (2017)	0.378	0.367	0.390	-19.829	0.000
	Khairizka Citra et al. (2019)	0.511	0.447	0.574	0.326	0.744
	Kowalski et al. (1999)	0.600	0.520	0.675	2.433	0.015
	Kuhn et al. (2020)a	0.377	0.351	0.404	-8.642	0.000
	Kuhn et al. (2020)b	0.516	0.482	0.551	0.925	0.355
	Lopez-Cevallos et al. (2019)	0.204	0.191	0.218	-31.878	0.000
	Mei et al. (2020)	0.288	0.215	0.373	-4.582	0.000
	Peng et al. (2022)	0.272	0.218	0.332	-6.685	0.000
	Sanskriti et al. (2024)	0.177	0.113	0.267	-5.746	0.000
	Shan et al. (2011)a	0.182	0.156	0.213	-15.402	0.000
	Shan et al. (2011)b	0.342	0.318	0.368	-11.556	0.000
	Sornlorm and Thi (2024)	0.399	0.352	0.447	-4.041	0.000
		0.362	0.359	0.365	-97.026	0.000
		0.439	0.399	0.479	-2.990	0.003
		0.362	0.359	0.365	-97.068	0.000
		0.427	0.390	0.465	-3.767	0.000

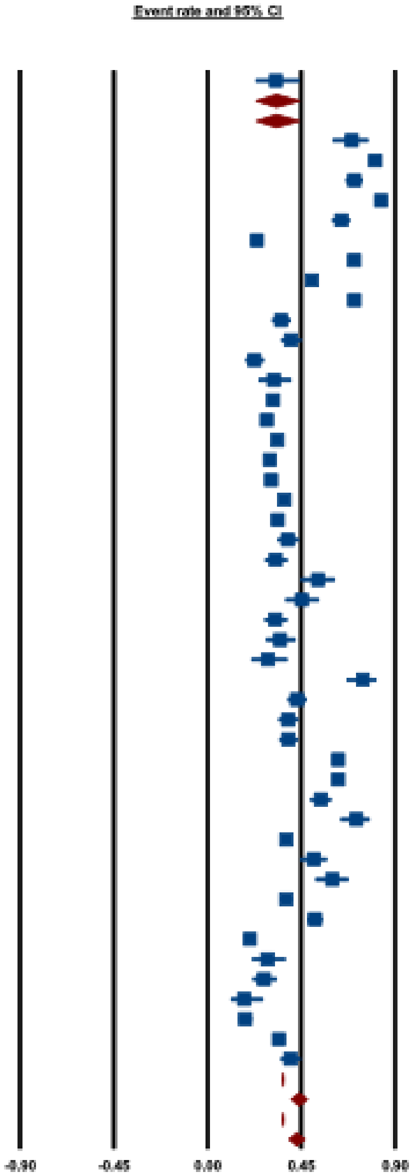

This figure illustrates the pooled prevalence of overweight/Obesity among migrant workers based on study design; (a) Cohort study was 32.9% (95% CI, 23.3 to 44.2 ; n = 76) and (b) Cross-sectional study setting was 43.9% (95% CI, 39.9 to 47.9; n = 135,328).

Appendix 32. Pooled Prevalence of Overweight/Obesity among Migrant Workers by Publish Year of Studies.

	Study name	Event rate	Lower limit	Upper limit	Z-Value	p-Value
(a)	Bi et al. (2015)a	0.315	0.311	0.319	-79.644	0.000
	Bi et al. (2015)b	0.285	0.275	0.295	-38.066	0.000
	Bi et al. (2015)c	0.336	0.326	0.347	-28.453	0.000
	Bi et al. (2015)d	0.300	0.290	0.310	-35.434	0.000
	Bi et al. (2015)e	0.306	0.296	0.316	-34.221	0.000
	Bi et al. (2015)f	0.370	0.360	0.381	-23.133	0.000
	Bi et al. (2015)g	0.338	0.328	0.349	-28.111	0.000
	Castaneda et al. (2015)a	0.326	0.274	0.383	-5.710	0.000
	Castaneda et al. (2015)b	0.349	0.282	0.422	-3.942	0.000
	Castaneda et al. (2015)c	0.290	0.212	0.382	-4.208	0.000
	Jorgensen et al. (2011)	0.714	0.640	0.779	5.252	0.000
	Shan et al. (2011)a	0.182	0.156	0.213	-15.402	0.000
	Shan et al. (2011)b	0.342	0.318	0.368	-11.556	0.000
		0.319	0.316	0.322	-111.690	0.000
		0.327	0.307	0.347	-15.858	0.000
(b)	Afrifa-Anane et al. (2020)a	0.806	0.780	0.829	17.644	0.000
	Afrifa-Anane et al. (2020)b	0.706	0.665	0.743	9.109	0.000
	Afrifa-Anane et al. (2020)c	0.833	0.807	0.856	17.883	0.000
	Afrifa-Anane et al. (2020)d	0.643	0.599	0.684	6.209	0.000
	Afrifa-Anane et al. (2020)e	0.238	0.212	0.265	-15.468	0.000
	Aung et al. (2019)	0.401	0.355	0.449	-4.003	0.000
	Carioca et al. (2017)a	0.387	0.337	0.439	-4.192	0.000
	Carioca et al. (2017)b	0.328	0.277	0.383	-5.803	0.000
	Castaneda et al. (2019)	0.747	0.670	0.811	5.679	0.000
	Charoensook et al. (2018)	0.430	0.386	0.476	-2.998	0.003
	Huang et al. (2020)a	0.628	0.602	0.653	9.447	0.000
	Huang et al. (2020)b	0.545	0.494	0.595	1.716	0.086
	Joy et al. (2017)	0.378	0.367	0.390	-19.829	0.000
	Khairizka Citra et al. (2019)	0.511	0.447	0.574	0.326	0.744
	Kuhn et al. (2020)a	0.377	0.351	0.404	-8.642	0.000
	Kuhn et al. (2020)b	0.516	0.482	0.551	0.925	0.355
	Lopez-Cevallos et al. (2018)	0.204	0.191	0.218	-31.878	0.000
	Mei et al. (2020)	0.288	0.215	0.373	-4.582	0.000
		0.425	0.418	0.433	-19.482	0.000
		0.501	0.407	0.595	0.022	0.982
(c)	Abasilim et al. (2024)	0.694	0.602	0.772	3.970	0.000
	Alhadlaq et al. (2023)a	0.704	0.689	0.719	23.340	0.000
	Alhadlaq et al. (2023)b	0.501	0.485	0.517	0.146	0.884
	Ali et al. (2022)	0.705	0.690	0.720	23.413	0.000
	Arslan et al. (2023)	0.353	0.311	0.399	-6.129	0.000
	Aung et al. (2024)	0.225	0.185	0.271	-9.799	0.000
	Bhandari et al. (2023)	0.319	0.248	0.400	-4.194	0.000
	Carioca et al. (2021)a	0.531	0.450	0.611	0.747	0.455
	Carioca et al. (2021)b	0.453	0.374	0.533	-1.149	0.251
	Dah Poe et al. (2022)	0.388	0.342	0.437	-4.450	0.000
	Emiral et al. (2021)	0.389	0.345	0.435	-4.695	0.000
	Hall et al. (2021)	0.628	0.602	0.653	9.447	0.000
	Peng et al. (2022)	0.272	0.218	0.332	-6.685	0.000
	Sanskriti et al. (2024)	0.177	0.113	0.267	-5.746	0.000
	Somlorm and Thi (2024)	0.399	0.352	0.447	-4.041	0.000
		0.580	0.572	0.588	18.708	0.000
		0.447	0.367	0.529	-1.270	0.204
		0.362	0.359	0.364	-97.171	0.000
		0.342	0.323	0.361	-15.249	0.000

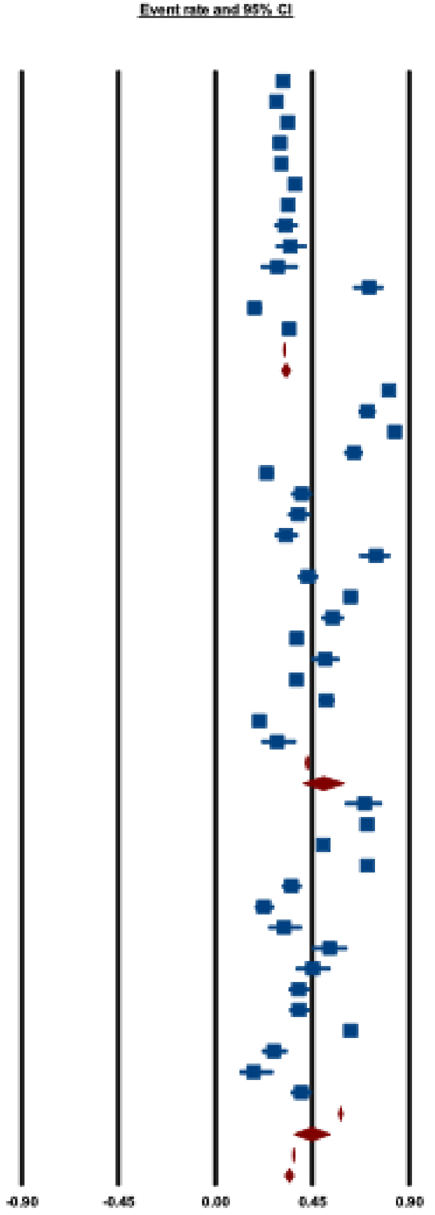

This figure illustrates the pooled prevalence of overweight/Obesity among migrant workers by publish year of studies; (a) In years 2011 - 2015 was 32.7% (95% CI, 30.7 to 34.7; n = 100,486), (b) In years 2016 - 2020 was 50.1% (95% CI, 40.7 to 59.5; n = 19,825) and (c) In years 2021 – 2025 was 44.7% (95% CI, 36.7 to 52.9; n = 15,093).

Appendix 33. Pooled Prevalence of Overweight/Obesity among Migrant Workers according to Study Settings

	Study name	Statistics for each study				
		Event rate	Lower limit	Upper limit	Z-Value	p-Value
(a)	Abasilim et al. (2024)	0.694	0.602	0.772	3.970	0.000
	Afrifa-Anane et al. (2020)e	0.238	0.212	0.265	-15.468	0.000
	Alhadlaq et al. (2023)a	0.704	0.689	0.719	23.340	0.000
	Alhadlaq et al. (2023)b	0.501	0.485	0.517	0.146	0.884
	Castaneda et al. (2015)a	0.326	0.274	0.383	-5.710	0.000
	Castaneda et al. (2015)b	0.349	0.282	0.422	-3.942	0.000
	Castaneda et al. (2015)c	0.290	0.212	0.382	-4.208	0.000
	Castaneda et al. (2019)	0.747	0.670	0.811	5.679	0.000
	Emiral et al. (2021)	0.389	0.345	0.435	-4.695	0.000
	Lopez-Cevallos et al. (2019)	0.204	0.191	0.218	-31.878	0.000
	Shan et al. (2011)a	0.182	0.156	0.213	-15.402	0.000
	Shan et al. (2011)b	0.342	0.318	0.368	-11.556	0.000
		0.438	0.430	0.447	-13.857	0.000
		0.404	0.286	0.534	-1.453	0.146
(b)	Afrifa-Anane et al. (2020)a	0.806	0.780	0.829	17.644	0.000
	Afrifa-Anane et al. (2020)b	0.706	0.665	0.743	9.109	0.000
	Afrifa-Anane et al. (2020)c	0.833	0.807	0.856	17.883	0.000
	Afrifa-Anane et al. (2020)d	0.643	0.599	0.684	6.209	0.000
	Ali et al. (2022)	0.705	0.690	0.720	23.413	0.000
	Arslan et al. (2023)	0.353	0.311	0.399	-6.129	0.000
	Aung et al. (2019)	0.401	0.355	0.449	-4.003	0.000
	Aung et al. (2024)	0.225	0.185	0.271	-9.799	0.000
	Bhandari et al. (2023)	0.319	0.248	0.400	-4.194	0.000
	Bi et al. (2015)a	0.315	0.311	0.319	-79.644	0.000
	Bi et al. (2015)b	0.285	0.275	0.295	-38.066	0.000
	Bi et al. (2015)c	0.336	0.326	0.347	-28.453	0.000
	Bi et al. (2015)d	0.300	0.290	0.310	-35.434	0.000
	Bi et al. (2015)e	0.306	0.296	0.316	-34.221	0.000
	Bi et al. (2015)f	0.370	0.360	0.381	-23.133	0.000
	Bi et al. (2015)g	0.338	0.328	0.349	-28.111	0.000
	Carioca et al. (2017)a	0.387	0.337	0.439	-4.192	0.000
	Carioca et al. (2017)b	0.328	0.277	0.383	-5.803	0.000
	Carioca et al. (2021)a	0.531	0.450	0.611	0.747	0.455
	Carioca et al. (2021)b	0.453	0.374	0.533	-1.149	0.251
	Charoensook et al. (2018)	0.430	0.386	0.476	-2.998	0.003
	Dah Poe et al. (2022)	0.388	0.342	0.437	-4.450	0.000
	Hall et al. (2021)	0.628	0.602	0.653	9.447	0.000
	Huang et al. (2020)a	0.628	0.602	0.653	9.447	0.000
	Huang et al. (2020)b	0.545	0.494	0.595	1.716	0.086
	Jorgensen et al. (2011)	0.714	0.640	0.779	5.252	0.000
	Joy et al. (2017)	0.378	0.367	0.390	-19.829	0.000
	Khairizka Citra et al. (2019)	0.511	0.447	0.574	0.326	0.744
	Kowalski et al. (1999)	0.600	0.520	0.675	2.433	0.015
	Kuhn et al. (2020)a	0.377	0.351	0.404	-8.642	0.000
	Kuhn et al. (2020)b	0.516	0.482	0.551	0.925	0.355
	Leong (2006)	0.329	0.233	0.442	-2.920	0.003
	Peng et al. (2022)	0.272	0.218	0.332	-6.685	0.000
	Sanskriti et al. (2024)	0.177	0.113	0.267	-5.746	0.000
	Somlorm and Thi (2024)	0.399	0.352	0.447	-4.041	0.000
		0.353	0.351	0.356	-97.792	0.000
		0.453	0.412	0.495	-2.208	0.027
		0.362	0.359	0.365	-96.975	0.000
		0.449	0.410	0.488	-2.545	0.011

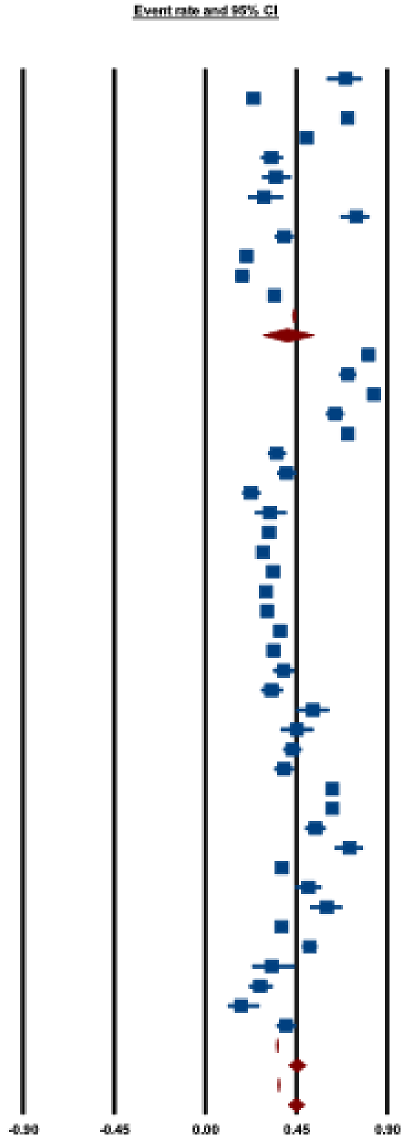

This figure illustrates the pooled prevalence of overweight/Obesity among migrant workers according to migrant setting; (a) Community setting was 42.3% (95% CI, 39.1 to 45.5; n = 124,287) and (b) Hospital setting was 53.7% (95% CI, 42.5 to 64.5; n = 11,126)

Appendix 34. Pooled Prevalence of Overweight/Obesity among Migrant Workers based on Sample size category

	Study name	Statistics for each study				
		Event rate	Lower limit	Upper limit	Z-Value	p-Value
(a)	Abasilim et al. (2024)	0.694	0.602	0.772	3.970	0.000
	Afrifa-Anane et al. (2020)a	0.806	0.780	0.829	17.644	0.000
	Afrifa-Anane et al. (2020)b	0.706	0.665	0.743	9.109	0.000
	Afrifa-Anane et al. (2020)c	0.833	0.807	0.856	17.883	0.000
	Afrifa-Anane et al. (2020)d	0.643	0.599	0.684	6.209	0.000
	Afrifa-Anane et al. (2020)e	0.238	0.212	0.265	-15.468	0.000
	Arslan et al. (2023)	0.353	0.311	0.399	-6.129	0.000
	Aung et al. (2019)	0.401	0.355	0.449	-4.003	0.000
	Aung et al. (2024)	0.225	0.185	0.271	-9.799	0.000
	Bhandari et al. (2023)	0.319	0.248	0.400	-4.194	0.000
	Carioca et al. (2017)a	0.387	0.337	0.439	-4.192	0.000
	Carioca et al. (2017)b	0.328	0.277	0.383	-5.803	0.000
	Carioca et al. (2021)a	0.531	0.450	0.611	0.747	0.455
	Carioca et al. (2021)b	0.453	0.374	0.533	-1.149	0.251
	Castaneda et al. (2015)a	0.326	0.274	0.383	-5.710	0.000
	Castaneda et al. (2015)b	0.349	0.282	0.422	-3.942	0.000
	Castaneda et al. (2015)c	0.290	0.212	0.382	-4.208	0.000
	Castaneda et al. (2019)	0.747	0.670	0.811	5.679	0.000
	Charoensook et al. (2018)	0.430	0.386	0.476	-2.998	0.003
	Dah Poe et al. (2022)	0.388	0.342	0.437	-4.450	0.000
	Emiral et al. (2021)	0.389	0.345	0.435	-4.695	0.000
	Huang et al. (2020)b	0.545	0.494	0.595	1.716	0.086
	Jorgensen et al. (2011)	0.714	0.640	0.779	5.252	0.000
	Khairizka Citra et al. (2019)	0.511	0.447	0.574	0.326	0.744
	Kowalski et al. (1999)	0.600	0.520	0.675	2.433	0.015
	Kuhn et al. (2020)b	0.516	0.482	0.551	0.925	0.355
	Leong (2006)	0.329	0.233	0.442	-2.920	0.003
	Mei et al. (2020)	0.288	0.215	0.373	-4.582	0.000
	Peng et al. (2022)	0.272	0.218	0.332	-6.685	0.000
	Sanskriti et al. (2024)	0.177	0.113	0.267	-5.746	0.000
	Shan et al. (2011)a	0.182	0.156	0.213	-15.402	0.000
	Somlorm and Thi (2024)	0.399	0.352	0.447	-4.041	0.000
		0.468	0.458	0.478	-6.222	0.000
		0.447	0.374	0.523	-1.375	0.169
(b)	Alhadlaq et al. (2023)a	0.704	0.689	0.719	23.340	0.000
	Alhadlaq et al. (2023)b	0.501	0.485	0.517	0.146	0.884
	Ali et al. (2022)	0.705	0.690	0.720	23.413	0.000
	Bi et al. (2015)a	0.315	0.311	0.319	-79.644	0.000
	Bi et al. (2015)b	0.285	0.275	0.295	-38.066	0.000
	Bi et al. (2015)c	0.336	0.326	0.347	-28.453	0.000
	Bi et al. (2015)d	0.300	0.290	0.310	-35.434	0.000
	Bi et al. (2015)e	0.306	0.296	0.316	-34.221	0.000
	Bi et al. (2015)f	0.370	0.360	0.381	-23.133	0.000
	Bi et al. (2015)g	0.338	0.328	0.349	-28.111	0.000
	Hall et al. (2021)	0.628	0.602	0.653	9.447	0.000
	Huang et al. (2020)a	0.628	0.602	0.653	9.447	0.000
	Joy et al. (2017)	0.378	0.367	0.390	-19.829	0.000
	Kuhn et al. (2020)a	0.377	0.351	0.404	-8.642	0.000
	Lopez-Cevallos et al. (2019)	0.204	0.191	0.218	-31.878	0.000
	Shan et al. (2011)b	0.342	0.318	0.368	-11.556	0.000
		0.353	0.350	0.356	-99.454	0.000
		0.415	0.358	0.475	-2.770	0.006
		0.362	0.359	0.365	-97.068	0.000
		0.428	0.382	0.474	-3.021	0.003

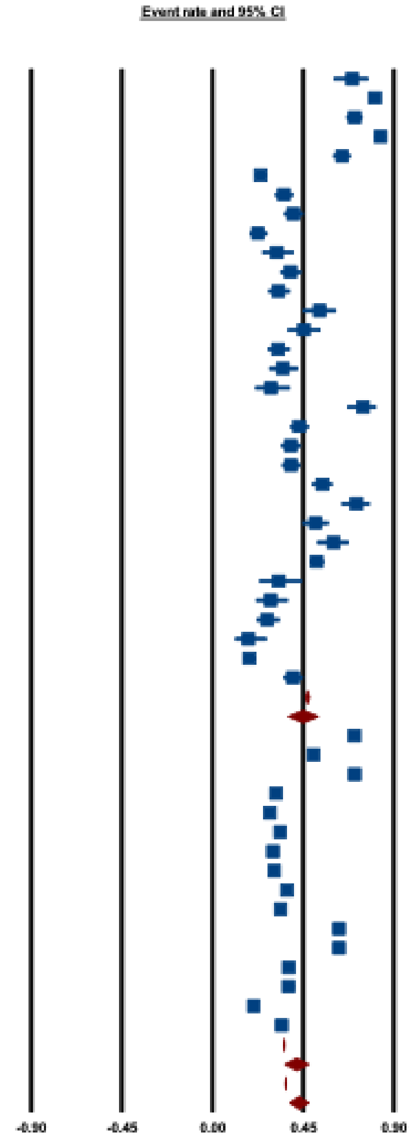

This figure illustrates the pooled prevalence of overweight/Obesity among migrant workers based on sample size category; (a) Study with sample less than 1000 was 44.7% (95% CI, 37.4 to 52.3; n = 11,651) and (b) Study with sample more than 1000 was 41.5% (95% CI, 35.8 to 47.5; n = 123,753)

Appendix 35. Pooled Prevalence of Overweight/Obesity among Migrant Workers according to Study Quality

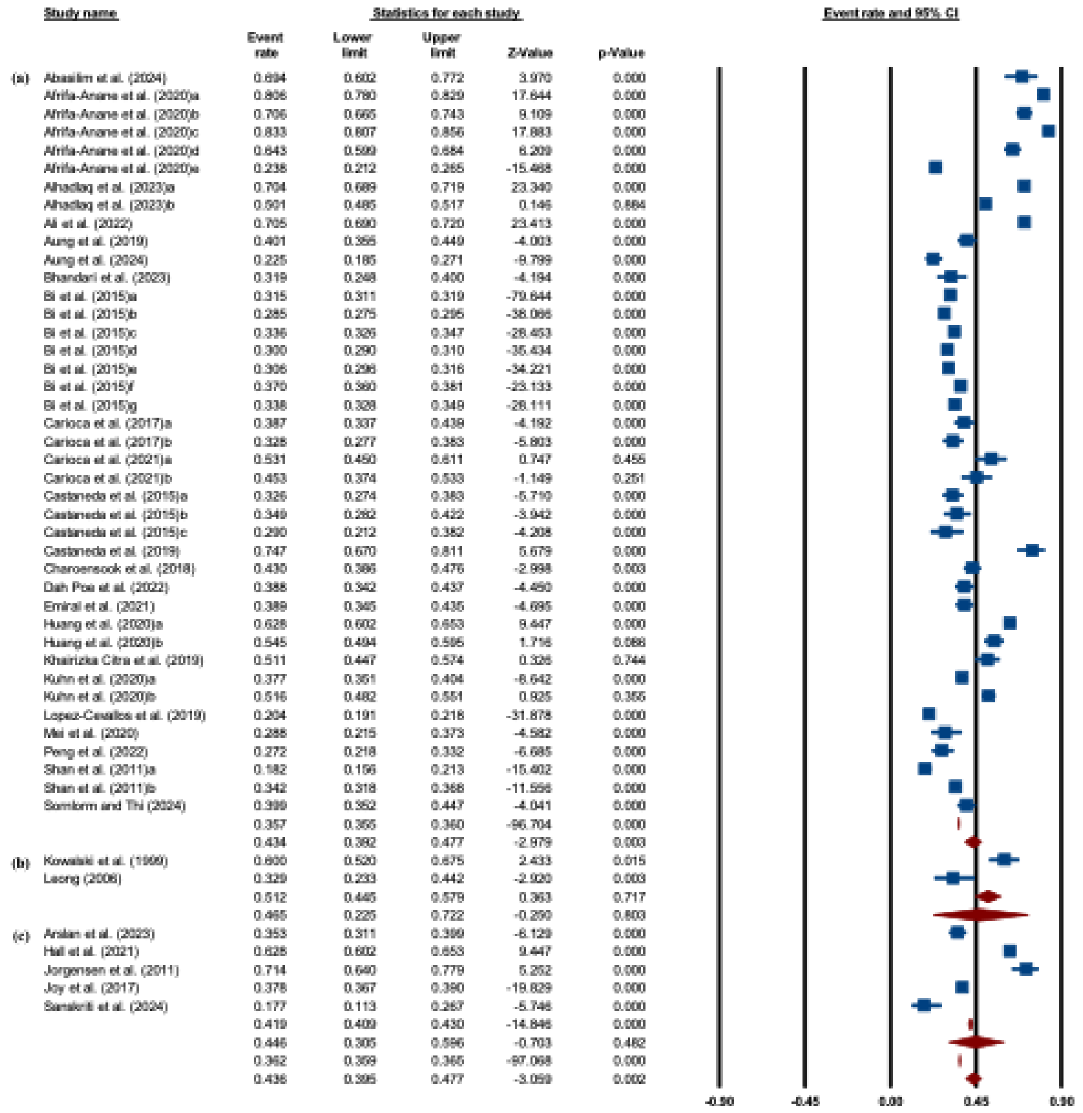

This figure illustrates the pooled prevalence of overweight/Obesity among migrant workers according to study quality; (a) High quality study was 43.4% (95% CI, 39.2 to 47.7; n = 126,308), (b) Low quality study was 46.5% (95% CI, 22.5 to 72.2; n = 3,884) and (c) Moderate quality study was 44.6% (95% CI, 30.5 to 59.6; n = 5,212).

Appendix 36. Pooled Prevalence of Overweight/Obesity among Migrant Workers based on Destination Continent

	Study name	Event rate	Lower limit	Upper limit	Z-Value	p-Value
		Statistics for each study				
(a)	Aung et al. (2019)	0.401	0.355	0.449	-4.003	0.000
	Aung et al. (2024)	0.225	0.185	0.271	-9.799	0.000
	Bhandari et al. (2023)	0.319	0.248	0.400	-4.194	0.000
	Bi et al. (2015)a	0.315	0.311	0.319	-79.644	0.000
	Bi et al. (2015)b	0.285	0.275	0.295	-38.066	0.000
	Bi et al. (2015)c	0.336	0.326	0.347	-28.453	0.000
	Bi et al. (2015)d	0.300	0.290	0.310	-35.434	0.000
	Bi et al. (2015)e	0.306	0.296	0.316	-34.221	0.000
	Bi et al. (2015)f	0.370	0.360	0.381	-23.133	0.000
	Bi et al. (2015)g	0.338	0.328	0.349	-28.111	0.000
	Charoensook et al. (2018)	0.430	0.386	0.476	-2.998	0.003
	Dah Poe et al. (2022)	0.388	0.342	0.437	-4.450	0.000
	Hall et al. (2021)	0.628	0.602	0.653	9.447	0.000
	Huang et al. (2020)a	0.628	0.602	0.653	9.447	0.000
	Huang et al. (2020)b	0.545	0.494	0.595	1.716	0.086
	Khairizka Citra et al. (2019)	0.511	0.447	0.574	0.326	0.744
	Leong (2006)	0.329	0.233	0.442	-2.920	0.003
	Mei et al. (2020)	0.288	0.215	0.373	-4.582	0.000
	Peng et al. (2022)	0.272	0.218	0.332	-6.685	0.000
	Shan et al. (2011)a	0.182	0.156	0.213	-15.402	0.000
	Shan et al. (2011)b	0.342	0.318	0.368	-11.556	0.000
	Somlorm and Thi (2024)	0.399	0.352	0.447	-4.041	0.000
		0.329	0.326	0.332	-108.108	0.000
		0.366	0.337	0.397	-8.388	0.000
(b)	Afrifa-Anane et al. (2020)a	0.806	0.780	0.829	17.644	0.000
	Afrifa-Anane et al. (2020)b	0.706	0.665	0.743	9.109	0.000
	Afrifa-Anane et al. (2020)c	0.833	0.807	0.856	17.883	0.000
	Arslan et al. (2023)	0.353	0.311	0.399	-6.129	0.000
	Emiral et al. (2021)	0.389	0.345	0.435	-4.695	0.000
	Jorgensen et al. (2011)	0.714	0.640	0.779	5.252	0.000
		0.665	0.647	0.682	17.212	0.000
		0.652	0.458	0.806	1.545	0.122
(c)	Carioca et al. (2017)a	0.387	0.337	0.439	-4.192	0.000
	Carioca et al. (2017)b	0.328	0.277	0.383	-5.803	0.000
	Carioca et al. (2021)a	0.531	0.450	0.611	0.747	0.455
	Carioca et al. (2021)b	0.453	0.374	0.533	-1.149	0.251
	Castaneda et al. (2019)	0.747	0.670	0.811	5.679	0.000
		0.442	0.412	0.473	-3.656	0.000
		0.489	0.357	0.621	-0.165	0.869
(d)	Alhadlaq et al. (2023)a	0.704	0.689	0.719	23.340	0.000
	Alhadlaq et al. (2023)b	0.501	0.485	0.517	0.146	0.884
	Ali et al. (2022)	0.705	0.690	0.720	23.413	0.000
	Kuhn et al. (2020)b	0.516	0.482	0.551	0.925	0.355
		0.620	0.611	0.629	25.025	0.000
		0.611	0.489	0.721	1.792	0.073
(e)	Abasilim et al. (2024)	0.694	0.602	0.772	3.970	0.000
	Castaneda et al. (2015)a	0.326	0.274	0.383	-5.710	0.000
	Castaneda et al. (2015)b	0.349	0.282	0.422	-3.942	0.000
	Castaneda et al. (2015)c	0.290	0.212	0.382	-4.208	0.000
	Kowalski et al. (1999)	0.600	0.520	0.675	2.433	0.015
	Lopez-Cevallos et al. (2019)	0.204	0.191	0.218	-31.878	0.000
		0.251	0.237	0.264	-29.663	0.000
		0.400	0.251	0.569	-1.163	0.245
(f)	Joy et al. (2017)	0.378	0.367	0.390	-19.829	0.000
	Kuhn et al. (2020)a	0.377	0.351	0.404	-8.642	0.000
	Sanskriti et al. (2024)	0.177	0.113	0.267	-5.746	0.000
		0.376	0.366	0.387	-22.044	0.000
		0.348	0.302	0.398	-5.775	0.000
(g)	Afrifa-Anane et al. (2020)d	0.643	0.599	0.684	6.209	0.000
	Afrifa-Anane et al. (2020)e	0.238	0.212	0.265	-15.468	0.000
		0.381	0.354	0.409	-8.227	0.000
		0.428	0.118	0.807	-0.331	0.741
		0.362	0.359	0.365	-97.068	0.000
		0.382	0.358	0.406	-9.182	0.000

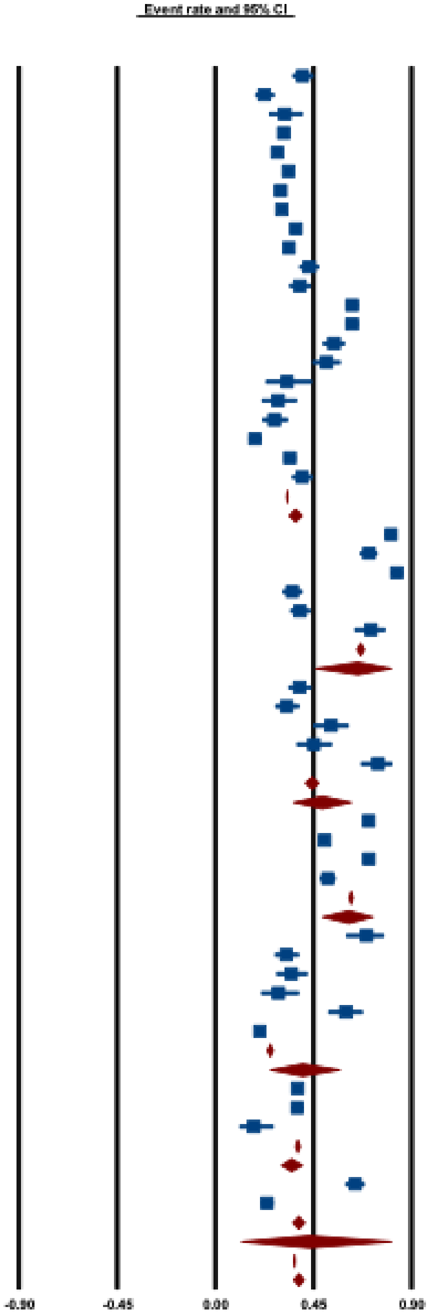

This figure illustrates the pooled prevalence of overweight/Obesity among migrant workers based on destination continent; (a) East Asia and Pacific was 36.6% (95% CI, 33.7 to 39.7; n = 105,551), (b) Europe & Central Asia was 65.2% (95% CI, 45.8 to 80.6; n = 3,463), (c) Latin America & Caribbean was 48.9% (95% CI, 35.7 to 62.1; n = 1,084), (d) Midle East & North Africa was 61.1% (95% CI, 48.9 to 72.1; n = 11,549), (e) North America was 41.1% (95% CI, 25.7 to 58.5; n = 4,198), (f) South Asia was 34.8% (95% CI, 30.2 to 39.8; n = 8,131) and (g) Sub-Saharan Africa was 42.8% (95% CI, 11.8 to 80.7; n = 1,459).

Appendix 37. Pooled Prevalence of Overweight/Obesity among Migrant Workers based on income level of destination country

	Study name	Event rate	Lower limit	Upper limit	Z-Value	p-Value
		Statistics for each study				
(a)	Abasilim et al. (2024)	0.694	0.602	0.772	3.970	0.000
	Afrifa-Anane et al. (2020)a	0.806	0.780	0.829	17.644	0.000
	Afrifa-Anane et al. (2020)b	0.706	0.665	0.743	9.109	0.000
	Afrifa-Anane et al. (2020)c	0.833	0.807	0.856	17.883	0.000
	Alhadiaq et al. (2023)a	0.704	0.689	0.719	23.340	0.000
	Alhadiaq et al. (2023)b	0.501	0.485	0.517	0.146	0.884
	Ali et al. (2022)	0.705	0.690	0.720	23.413	0.000
	Bhandari et al. (2023)	0.319	0.248	0.400	-4.194	0.000
	Castaneda et al. (2015)a	0.326	0.274	0.383	-5.710	0.000
	Castaneda et al. (2015)b	0.349	0.282	0.422	-3.942	0.000
	Castaneda et al. (2015)c	0.290	0.212	0.382	-4.208	0.000
	Hall et al. (2021)	0.628	0.602	0.653	9.447	0.000
	Huang et al. (2020)a	0.628	0.602	0.653	9.447	0.000
	Huang et al. (2020)b	0.545	0.494	0.595	1.718	0.086
	Jorgensen et al. (2011)	0.714	0.640	0.779	5.252	0.000
	Khairizka Citra et al. (2019)	0.511	0.447	0.574	0.326	0.744
	Kowalski et al. (1999)	0.600	0.520	0.675	2.433	0.015
	Kuhn et al. (2020)b	0.516	0.482	0.551	0.925	0.355
	Lopez-Cevallos et al. (2019)	0.204	0.191	0.218	-31.878	0.000
	Shan et al. (2011)a	0.182	0.156	0.213	-15.402	0.000
	Shan et al. (2011)b	0.342	0.318	0.368	-11.556	0.000
		0.549	0.542	0.556	13.978	0.000
		0.532	0.438	0.622	0.667	0.505
(b)	Afrifa-Anane et al. (2020)d	0.643	0.599	0.684	6.209	0.000
	Afrifa-Anane et al. (2020)e	0.238	0.212	0.265	-15.468	0.000
	Joy et al. (2017)	0.378	0.367	0.390	-19.829	0.000
	Kuhn et al. (2020)a	0.377	0.351	0.404	-8.642	0.000
	Sanskriti et al. (2024)	0.177	0.113	0.267	-5.748	0.000
		0.377	0.367	0.387	-23.527	0.000
		0.358	0.266	0.462	-2.647	0.008
(c)	Arslan et al. (2023)	0.353	0.311	0.399	-6.129	0.000
	Aung et al. (2019)	0.401	0.355	0.449	-4.003	0.000
	Aung et al. (2024)	0.225	0.185	0.271	-9.799	0.000
	Bi et al. (2015)a	0.315	0.311	0.319	-79.644	0.000
	Bi et al. (2015)b	0.285	0.275	0.295	-38.066	0.000
	Bi et al. (2015)c	0.336	0.326	0.347	-28.453	0.000
	Bi et al. (2015)d	0.300	0.290	0.310	-35.434	0.000
	Bi et al. (2015)e	0.306	0.296	0.316	-34.221	0.000
	Bi et al. (2015)f	0.370	0.360	0.381	-23.133	0.000
	Bi et al. (2015)g	0.338	0.328	0.349	-28.111	0.000
	Carioca et al. (2017)a	0.387	0.337	0.439	-4.192	0.000
	Carioca et al. (2017)b	0.328	0.277	0.383	-5.803	0.000
	Carioca et al. (2021)a	0.531	0.450	0.611	0.747	0.455
	Carioca et al. (2021)b	0.453	0.374	0.533	-1.149	0.251
	Castaneda et al. (2019)	0.747	0.670	0.811	5.679	0.000
	Charoensook et al. (2018)	0.430	0.386	0.476	-2.998	0.003
	Dah Poe et al. (2022)	0.388	0.342	0.437	-4.450	0.000
	Emiral et al. (2021)	0.389	0.345	0.435	-4.695	0.000
	Leong (2006)	0.329	0.233	0.442	-2.920	0.003
	Mei et al. (2020)	0.288	0.215	0.373	-4.582	0.000
	Peng et al. (2022)	0.272	0.218	0.332	-6.685	0.000
	Somlorm and Thi (2024)	0.399	0.352	0.447	-4.041	0.000
		0.322	0.319	0.325	-110.972	0.000
		0.357	0.339	0.375	-14.919	0.000
		0.362	0.359	0.365	-97.068	0.000
		0.364	0.347	0.381	-14.708	0.000

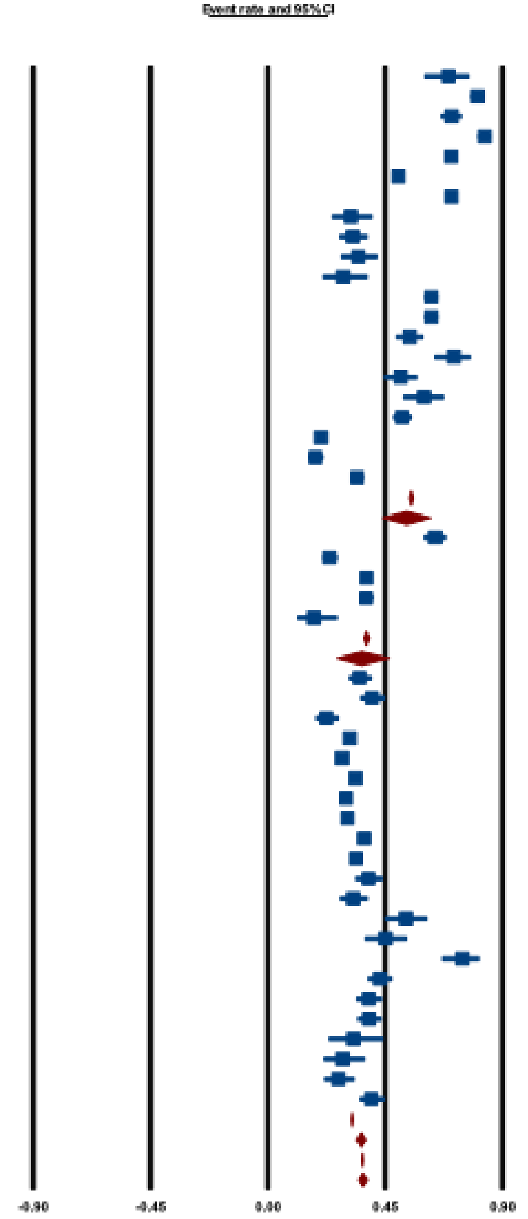

This figure illustrates the pooled prevalence of overweight/Obesity among migrant workers based on income level on destination country; (a) High income country was 53.2% (95% CI, 43.9 to 62.6; n = 27,413), (b) Lower middle income country was 35.8% (95% CI, 26.6 to 46.2, n = 9,590) and (c) Upper middle income country was 35.7% (95% CI, 33.9 to 37.5; n = 98,401)

Appendix 38. Pooled Prevalence of Overweight/Obesity among Migrant Workers according by Marital status

	Study name	Event rate	Lower limit	Upper limit	Z-Value	p-Value
(a)	Arslan et al. (2023)2	0.787	0.746	0.822	11.340	0.000
	Aung et al. (2019)2	0.700	0.655	0.743	7.918	0.000
	Aung et al. (2024)2	0.556	0.504	0.606	2.104	0.035
	Bhandari et al. (2023)2	0.567	0.485	0.647	1.595	0.111
	Charoensook et al. (2018)2	0.803	0.764	0.837	12.077	0.000
	Dah Poe et al. (2022)2	0.801	0.759	0.837	11.147	0.000
	Emiral et al. (2021)2	0.866	0.831	0.894	13.558	0.000
	Hall et al. (2021)2	0.516	0.490	0.542	1.181	0.238
	Huang et al. (2020)a2	0.445	0.419	0.471	-4.125	0.000
	Huang et al. (2020)b2	0.379	0.331	0.430	-4.587	0.000
	Joy et al. (2017)2	0.877	0.869	0.885	53.125	0.000
	Lopez-Cevallos et al. (2019)2	0.487	0.470	0.504	-1.479	0.139
	Mei et al. (2020)2	0.600	0.512	0.682	2.221	0.026
	Peng et al. (2022)2	0.914	0.870	0.944	10.093	0.000
	Somlorm and Thi (2024)2	0.680	0.633	0.723	7.078	0.000
		0.656	0.648	0.664	35.073	0.000
		0.690	0.573	0.787	3.092	0.002
(b)	Arslan et al. (2023)1	0.133	0.105	0.168	-13.498	0.000
	Aung et al. (2019)1	0.300	0.257	0.345	-7.918	0.000
	Aung et al. (2024)1	0.278	0.234	0.326	-8.120	0.000
	Bhandari et al. (2023)1	0.433	0.353	0.515	-1.595	0.111
	Charoensook et al. (2018)1	0.197	0.163	0.236	-12.077	0.000
	Dah Poe et al. (2022)1	0.199	0.163	0.241	-11.147	0.000
	Emiral et al. (2021)1	0.134	0.106	0.169	-13.558	0.000
	Hall et al. (2021)1	0.254	0.232	0.278	-17.452	0.000
	Huang et al. (2020)a1	0.254	0.232	0.278	-17.452	0.000
	Huang et al. (2020)b1	0.195	0.158	0.239	-10.788	0.000
	Joy et al. (2017)1	0.123	0.115	0.131	-53.125	0.000
	Lopez-Cevallos et al. (2019)1	0.386	0.370	0.403	-13.123	0.000
	Mei et al. (2020)1	0.352	0.273	0.439	-3.259	0.001
	Peng et al. (2022)1	0.086	0.056	0.130	-10.093	0.000
	Somlorm and Thi (2024)1	0.320	0.277	0.367	-7.078	0.000
		0.238	0.231	0.245	-60.242	0.000
		0.228	0.173	0.296	-6.827	0.000
(c)	Arslan et al. (2023)3	0.080	0.058	0.109	-14.056	0.000
	Hall et al. (2021)3	0.230	0.208	0.253	-18.955	0.000
	Huang et al. (2020)a3	0.301	0.278	0.326	-14.388	0.000
	Huang et al. (2020)b3	0.423	0.373	0.474	-2.955	0.003
	Mei et al. (2020)3	0.048	0.022	0.103	-7.140	0.000
		0.268	0.254	0.283	-25.910	0.000
		0.191	0.121	0.287	-5.269	0.000
		0.426	0.420	0.432	-23.683	0.000
		0.318	0.265	0.375	-5.900	0.000

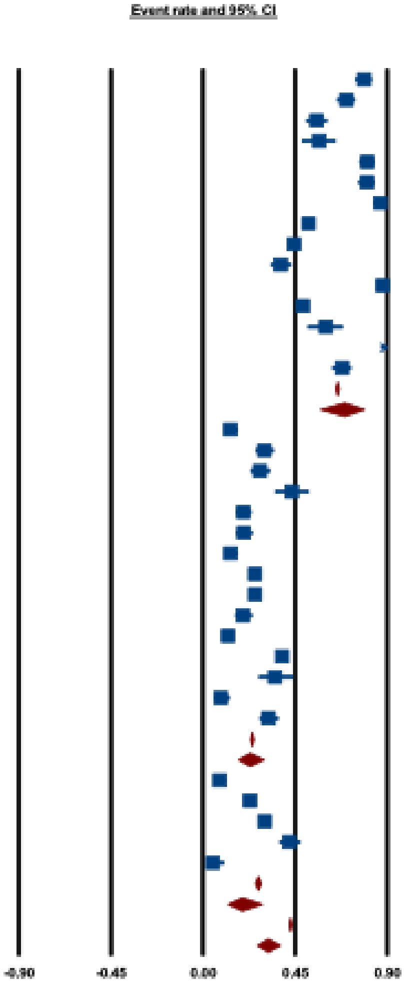

This figure illustrates the pooled prevalence of overweight/Obesity among migrant workers according by marital status; (a) Married was 69.0% (95% CI, 57.3 to 78.7; n = 16,754), (b) Single was 22.8% (95% CI, 17.3 to 29.6; n = 16,754) and (c) Widow or widower was 19.1% (95% CI, 12.1 to 28.7; n = 1,109).

Appendix 39. Pooled Prevalence of Overweight/Obesity among Migrant Workers by Education Level

	Study name	Statistics for each study				
		Event rate	Lower limit	Upper limit	Z-Value	p-Value
(a)	Abasilim et al. (2024)	0.342	0.260	0.435	-3.264	0.001
	Afrifa-Anane et al. (2020)a	0.776	0.749	0.801	16.251	0.000
	Afrifa-Anane et al. (2020)b	0.608	0.565	0.649	4.902	0.000
	Afrifa-Anane et al. (2020)c	0.448	0.415	0.481	-3.110	0.002
	Afrifa-Anane et al. (2020)d	0.727	0.686	0.765	9.625	0.000
	Afrifa-Anane et al. (2020)e	0.889	0.868	0.907	20.374	0.000
	Arslan et al. (2023)	0.240	0.203	0.282	-10.443	0.000
	Aung et al. (2019)	0.763	0.720	0.802	10.126	0.000
	Aung et al. (2024)	0.531	0.479	0.582	1.159	0.247
	Bhandari et al. (2023)	0.149	0.099	0.218	-7.369	0.000
	Bi et al. (2015)a	0.524	0.520	0.528	10.590	0.000
	Bi et al. (2015)b	0.481	0.470	0.492	-3.490	0.000
	Bi et al. (2015)c	0.572	0.561	0.583	12.684	0.000
	Bi et al. (2015)d	0.620	0.610	0.630	21.693	0.000
	Bi et al. (2015)e	0.405	0.394	0.416	-17.128	0.000
	Bi et al. (2015)f	0.607	0.596	0.618	19.101	0.000
	Bi et al. (2015)g	0.516	0.505	0.527	2.828	0.005
	Carioca et al. (2017)a	0.688	0.637	0.734	6.834	0.000
	Carioca et al. (2017)b	0.841	0.795	0.879	10.484	0.000
	Carioca et al. (2021)a	0.476	0.396	0.557	-0.581	0.561
	Carioca et al. (2021)b	0.655	0.575	0.727	3.717	0.000
	Castaneda et al. (2015)a	0.777	0.724	0.821	8.715	0.000
	Castaneda et al. (2015)b	0.771	0.703	0.828	6.757	0.000
	Castaneda et al. (2015)c	0.785	0.697	0.853	5.504	0.000
	Castaneda et al. (2019)	0.966	0.920	0.986	7.338	0.000
	Dah Poe et al. (2022)	0.346	0.301	0.394	-6.081	0.000
	Hall et al. (2021)	0.018	0.012	0.027	-19.812	0.000
	Huang et al. (2020)a	0.018	0.012	0.027	-19.812	0.000
	Joy et al. (2017)	0.248	0.238	0.258	-39.476	0.000
	Khairizka Citra et al. (2019)	0.123	0.087	0.172	-9.885	0.000
	Peng et al. (2022)	0.888	0.840	0.923	9.945	0.000
	Shan et al. (2011)a	0.175	0.149	0.205	-15.652	0.000
	Shan et al. (2011)b	0.210	0.190	0.233	-20.122	0.000
	Sornlorm and Thi (2024)	0.261	0.221	0.306	-9.207	0.000
		0.512	0.509	0.515	7.732	0.000
		0.492	0.445	0.539	-0.320	0.749
(b)	Abasilim et al. (2024)1	0.577	0.483	0.665	1.607	0.108
	Afrifa-Anane et al. (2020)a1	0.224	0.199	0.251	-16.251	0.000
	Afrifa-Anane et al. (2020)b1	0.392	0.351	0.435	-4.902	0.000
	Afrifa-Anane et al. (2020)c1	0.552	0.519	0.585	3.110	0.002
	Afrifa-Anane et al. (2020)d1	0.273	0.235	0.314	-9.625	0.000
	Afrifa-Anane et al. (2020)e1	0.111	0.093	0.132	-20.374	0.000
	Arslan et al. (2023)1	0.760	0.718	0.797	10.443	0.000
	Aung et al. (2019)1	0.237	0.198	0.280	-10.126	0.000
	Aung et al. (2024)1	0.469	0.418	0.521	-1.159	0.247
	Bhandari et al. (2023)1	0.851	0.782	0.901	7.369	0.000
	Bi et al. (2015)a1	0.476	0.472	0.480	-10.590	0.000
	Bi et al. (2015)b1	0.519	0.508	0.530	3.490	0.000
	Bi et al. (2015)c1	0.428	0.417	0.439	-12.706	0.000
	Bi et al. (2015)d1	0.380	0.370	0.390	-21.693	0.000
	Bi et al. (2015)e1	0.595	0.584	0.606	17.128	0.000
	Bi et al. (2015)f1	0.393	0.382	0.404	-19.123	0.000
	Bi et al. (2015)g1	0.484	0.473	0.495	-2.828	0.005
	Carioca et al. (2017)a1	0.312	0.266	0.363	-6.834	0.000
	Carioca et al. (2017)b1	0.159	0.121	0.205	-10.484	0.000
	Carioca et al. (2021)a1	0.524	0.443	0.604	0.581	0.561
	Carioca et al. (2021)b1	0.345	0.273	0.425	-3.717	0.000
	Castaneda et al. (2015)a1	0.223	0.179	0.276	-8.715	0.000
	Castaneda et al. (2015)b1	0.229	0.172	0.297	-6.757	0.000
	Castaneda et al. (2015)c1	0.215	0.147	0.303	-5.504	0.000
	Castaneda et al. (2019)1	0.034	0.014	0.080	-7.338	0.000
	Dah Poe et al. (2022)1	0.654	0.606	0.699	6.081	0.000
	Hall et al. (2021)1	0.982	0.973	0.988	19.812	0.000
	Huang et al. (2020)a1	0.982	0.973	0.988	19.812	0.000
	Joy et al. (2017)1	0.752	0.742	0.762	39.476	0.000
	Khairizka Citra et al. (2019)1	0.877	0.828	0.913	9.885	0.000
	Peng et al. (2022)1	0.112	0.077	0.160	-9.945	0.000
	Shan et al. (2011)a1	0.810	0.780	0.838	15.143	0.000
	Shan et al. (2011)b1	0.790	0.767	0.810	20.122	0.000
	Sornlorm and Thi (2024)1	0.983	0.964	0.992	10.604	0.000
		0.488	0.485	0.491	-8.130	0.000
		0.518	0.471	0.565	0.753	0.451
		0.500	0.498	0.502	-0.275	0.784
		0.505	0.472	0.539	0.304	0.761

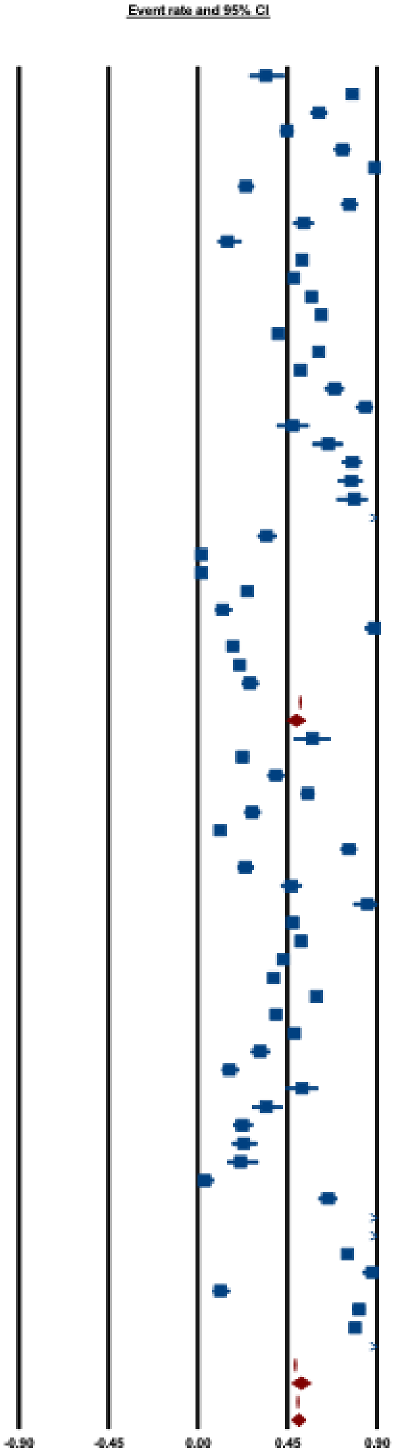

This figure illustrates the pooled prevalence of overweight/Obesity among migrant workers by education level; (a) Education level that lower than secondary level was 49.2% (95% CI, 44.5 to 53.9; n = 117,314) and (b) Education level that more than secondary level was 51.8% (95% CI, 47.1 to 56.5; n = 117,314)

Appendix 40. Pooled Prevalence of Overweight/Obesity among Migrant Workers according to Migrant Setting

	Study name	Statistics for each study				
		Event rate	Lower limit	Upper limit	Z-Value	p-Value
(a)	Abasilim et al. (2024)	0.694	0.602	0.772	3.970	0.000
	Afrifa-Anane et al. (2020)e	0.238	0.212	0.265	-15.468	0.000
	Alhadlaq et al. (2023)a	0.704	0.689	0.719	23.340	0.000
	Alhadlaq et al. (2023)b	0.501	0.485	0.517	0.146	0.884
	Castaneda et al. (2015)a	0.326	0.274	0.383	-5.710	0.000
	Castaneda et al. (2015)b	0.349	0.282	0.422	-3.942	0.000
	Castaneda et al. (2015)c	0.290	0.212	0.382	-4.208	0.000
	Castaneda et al. (2019)	0.747	0.670	0.811	5.679	0.000
	Emiral et al. (2021)	0.389	0.345	0.435	-4.695	0.000
	Lopez-Cevallos et al. (2019)	0.204	0.191	0.218	-31.878	0.000
	Shan et al. (2011)a	0.182	0.156	0.213	-15.402	0.000
	Shan et al. (2011)b	0.342	0.318	0.368	-11.556	0.000
		0.438	0.430	0.447	-13.857	0.000
		0.404	0.286	0.534	-1.453	0.146
(b)	Afrifa-Anane et al. (2020)a	0.806	0.780	0.829	17.644	0.000
	Afrifa-Anane et al. (2020)b	0.706	0.665	0.743	9.109	0.000
	Afrifa-Anane et al. (2020)c	0.833	0.807	0.856	17.883	0.000
	Afrifa-Anane et al. (2020)d	0.643	0.599	0.684	6.209	0.000
	Ali et al. (2022)	0.705	0.690	0.720	23.413	0.000
	Arslan et al. (2023)	0.353	0.311	0.399	-6.129	0.000
	Aung et al. (2019)	0.401	0.355	0.449	-4.003	0.000
	Aung et al. (2024)	0.225	0.185	0.271	-9.799	0.000
	Bhandari et al. (2023)	0.319	0.248	0.400	-4.194	0.000
	Bi et al. (2015)a	0.315	0.311	0.319	-79.644	0.000
	Bi et al. (2015)b	0.285	0.275	0.295	-38.066	0.000
	Bi et al. (2015)c	0.336	0.326	0.347	-28.453	0.000
	Bi et al. (2015)d	0.300	0.290	0.310	-35.434	0.000
	Bi et al. (2015)e	0.306	0.296	0.316	-34.221	0.000
	Bi et al. (2015)f	0.370	0.360	0.381	-23.133	0.000
	Bi et al. (2015)g	0.338	0.328	0.349	-28.111	0.000
	Carioca et al. (2017)a	0.387	0.337	0.439	-4.192	0.000
	Carioca et al. (2017)b	0.328	0.277	0.383	-5.803	0.000
	Carioca et al. (2021)a	0.531	0.450	0.611	0.747	0.455
	Carioca et al. (2021)b	0.453	0.374	0.533	-1.149	0.251
	Charoensook et al. (2018)	0.430	0.386	0.476	-2.998	0.003
	Dah Poe et al. (2022)	0.388	0.342	0.437	-4.450	0.000
	Hall et al. (2021)	0.628	0.602	0.653	9.447	0.000
	Huang et al. (2020)a	0.628	0.602	0.653	9.447	0.000
	Huang et al. (2020)b	0.545	0.494	0.595	1.716	0.086
	Jorgensen et al. (2011)	0.714	0.640	0.779	5.252	0.000
	Joy et al. (2017)	0.378	0.367	0.390	-19.829	0.000
	Khairizka Citra et al. (2019)	0.511	0.447	0.574	0.326	0.744
	Kowalski et al. (1999)	0.600	0.520	0.675	2.433	0.015
	Kuhn et al. (2020)a	0.377	0.351	0.404	-8.642	0.000
	Kuhn et al. (2020)b	0.516	0.482	0.551	0.925	0.355
	Leong (2006)	0.329	0.233	0.442	-2.920	0.003
	Peng et al. (2022)	0.272	0.218	0.332	-6.685	0.000
	Sanskriti et al. (2024)	0.177	0.113	0.267	-5.746	0.000
	Somlorm and Thi (2024)	0.399	0.352	0.447	-4.041	0.000
		0.353	0.351	0.356	-97.792	0.000
		0.453	0.412	0.495	-2.208	0.027
		0.362	0.359	0.365	-96.975	0.000
		0.449	0.410	0.488	-2.545	0.011

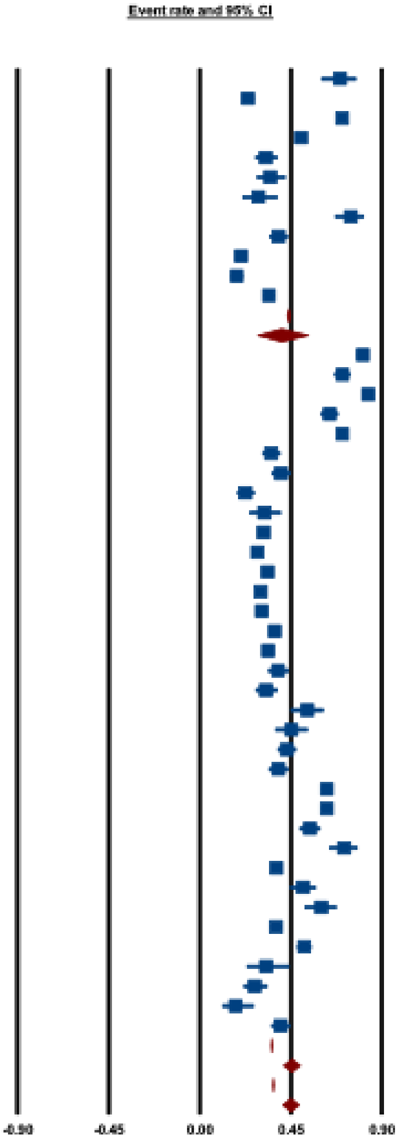

This figure illustrates the pooled prevalence of overweight/Obesity among migrant workers according to migrant setting; (a) Rural setting was 40.4% (95% CI, 28.6 to 53.4; n = 15,006) and (b) Urban setting was 45.3% (95% CI, 41.2 to 49.5; n = 120,398).

References

1 Stroup DF, Berlin JA, Morton SC, Olkin I, Williamson GD, Rennie D, et al. Meta-analysis of Observational Studies in EpidemiologyA Proposal for Reporting. JAMA. 2000;283:2008-12.

2 PRISMA. PRISMA 2020 Checklist. 2021. Available: https://www.prisma-statement.org/prisma-2020-checklist. Accessed.

3 Migration IOf. Glossary on Migration. 2021. Available: https://publications.iom.int/system/files/pdf/iml_34_glossary.pdf. Accessed.

4 ILO. Labour Migration. 2021. Available: https://www.ilo.org/topics-and-sectors/labour-migration#about. Accessed.

5 ILO. Media-Friendly Glossary on Migration. Moscow, Russia: International Labor Migration; 2022.

6 UN. International Convention on the Protection of the Rights of All Migrant Workers and Members of Their Families. New York: United Nation, 1990.

7 ILO. Temporary labour migration. Internation Labor Organization, 2022.

8 Munn Z, Moola S, Lisy K, Riitano D, Tufanaru C. Methodological guidance for systematic reviews of observational epidemiological studies reporting prevalence and cumulative incidence data. JBI Evidence Implementation. 2015;13:147-53.

9 Munn Z, Moola S, Riitano D, Lisy K. The Development of a Critical Appraisal Tool for Use in Systematic Reviews: Addressing Questions of Prevalence. International Journal of Health Policy and Management. 2014;3:123-8.

10 Kazeminia M, Afshar ZM, Rajati M, Saeedi A, Rajati F. Evaluation of the Acceptance Rate of Covid-19 Vaccine and its Associated Factors: A Systematic Review and Meta-analysis. J Prev (2022). 2022;43:421-67.

11 Abasilim C, Friedman LS, Martin MC, Madigan D, Perez J, Morera M, et al. Risk factors associated with indicators of dehydration among migrant farmworkers. Environ Res. 2024;251:118633.

12 Afrifa-Anane E, A DE-GA, K ACM, Beune E, Addo J, Smeeth L, et al. Physical Inactivity among Ghanaians in Ghana and Ghanaian Migrants in Europe. Med Sci Sports Exerc. 2020;52:2152-61.

13 Alhadlaq HW, Ateeq A, Shayea AMF, Gasana J. Occupational Asthma Prevalence among Migrant Workers Attending Shuaiba Industrial Medical Center in Kuwait. Healthcare (Basel). 2023;11.

14 Ali A, Alfajjam S, Gasana J. Diabetes Mellitus and Its Risk Factors among Migrant Workers in Kuwait. Int J Environ Res Public Health. 2022;19.

15 Arslan N, Çifci S, Bayram Değer V. Nutritional status of Syrian female migrants in Turkey: a cross-sectional study. International Journal of Public Health Science (IJPHS). 2023;12.

16 Aung TNN, Shirayama Y, Moolphate S, Aung MN, Lorga T, Yuasa M. Health risk behaviors, musculoskeletal disorders and associated cultural adaptation, depression: a survey among Myanmar migrant workers in Chiangmai, Northern Thailand. Int J Gen Med. 2019;12:283-92.

17 Aung TNN, Shirayama Y, Moolphate S, Lorga T, Angkurawaranon C, Yuasa M, et al. Prevalence and social determinants of depression: A cross-sectional survey of Myanmar migrant workers in Chiang Mai, Northern Thailand. Glob Public Health. 2024;19:2334316.

18 Bhandari P. Prevalence of cardiovascular risk factors among Asian migrant workers in South Korea. PLoS One. 2023;18:e0288375.

19 Bi Y, Wang L, Xu Y, Jiang Y, He J, Zhang M, et al. Diabetes-related metabolic risk factors in internal migrant workers in China: a national surveillance study. Lancet Diabetes Endocrinol. 2016;4:125-35.

20 Carioca AAF, Gorgulho B, Teixeira JA, Fisberg RM, Marchioni DM. Dietary patterns in internal migrants in a continental country: A population-based study. PLoS One. 2017;12:e0185882.

21 Carioca AAF, Gorgulho B, de Mello Fontanelli M, Fisberg RM, Marchioni DM. Cardiometabolic risk profile and diet quality among internal migrants in Brazil: a population-based study. Eur J Nutr. 2021;60:759-68.
22 Castaneda SF, Rosenbaum RP, Holscher JT, Madanat H, Talavera GA. Cardiovascular disease risk factors among Latino migrant and seasonal farmworkers. J Agromedicine. 2015;20:95-104.
23 Castaneda J, Caire-Juvera G, Sandoval S, Castaneda PA, Contreras AD, Portillo GE, et al. Food Security and Obesity among Mexican Agricultural Migrant Workers. Int J Environ Res Public Health. 2019;16.
24 Charoensook P, Upala P, Anuwatnonthakate A, Ruanjai T, Apidechkul T. Pulmonary tuberculosis screening and quality of life among migrant workers, Northern Thailand. J Infect Dev Ctries. 2018;12:1052-61.
25 Dah Poe NE, Srichan P, Khunthason S, Apidechkul T, Suttana W. Prevalence and Factors Associated with Hyperglycemia among Myanmar Migrant Workers in Mueang District, Chiang Rai Province, Thailand: a Cross-Sectional Study. Journal of Health Science and Medical Research. 2022.
26 Emiral GO, Onsuz MF, Ozay O, Isikli B, Metintas S. Cardiovascular Disease Risk Factors among Migrant Seasonal Agricultural Workers- Comparison with Local Residents. Iran J Public Health. 2021;50:747-55
27 Hall BJ, Huang L, Yi G, Latkin C. Fast food restaurant density and weight status: A spatial analysis among Filipina migrant workers in Macao (SAR), People's Republic of China. Soc Sci Med. 2021;269:113192.
28 Huang L, Chen W, Renzaho AMN, Hall BJ. Validation of Obesity Status Based on Self-Reported Data among Filipina and Indonesian Female Migrant Domestic Workers in Macao (SAR), China. Int J Environ Res Public Health. 2020;17.
29 Jorgensen MB, Rasmussen CD, Carneiro IG, Flyvholm MA, Olesen K, Ekner D, et al. Health disparities between immigrant and Danish cleaners. Int Arch Occup Environ Health. 2011;84:665-74.
30 Joy EJ, Green R, Agrawal S, Aleksandrowicz L, Bowen L, Kinra S, et al. Dietary patterns and non-communicable disease risk in Indian adults: secondary analysis of Indian Migration Study data. Public Health Nutr. 2017;20:1963-72.
31 Khairizka Citra P, Jung-Su C, Chun-Kuang S. Dietary Pattern and Anemia among Indonesian Female Migrant Workers in Taiwan. 2019. p. 37-47.
32 Kowalski K, Hoffman CJ, McClure A. Nutritional patterns and needs of migrant farm workers in northwest Michigan. J Am Diet Assoc. 1999;99:221-4.
33 Kuhn R, Barham T, Razzaque A, Turner P. Health and well-being of male international migrants and non-migrants in Bangladesh: A cross-sectional follow-up study. PLoS Med. 2020;17:e1003081.
34 Leong CC. Pre-Employment Medical Examination of Indonesian

Domestic Helpers in a Private Clinic in Johor Bahru - An Eight Year Review. Med J Malaysia. 2006;61:592-8.
35 Lopez-Cevallos DF, Escutia G, Gonzalez-Pena Y, Garside LI. Cardiovascular disease risk factors among Latino farmworkers in Oregon. Ann Epidemiol. 2019;40:8-12 e1.
36 Mei CF, Faller EM, Chuan LX, Gabriel JS. Household Income, Food Insecurity and Nutritional Status of Migrant Workers in Klang Valley, Malaysia. Ann Glob Health. 2020;86:90.
37 Peng H, Sun M, Hu X, Han H, Su J, Peng E, et al. Prevalence, awareness, and associated factors of high blood pressure among female migrant workers in Central South China. PeerJ. 2022;10:e13365.
38 Sanskriti J, Supriya V, Hemamalini AJ. Food Security, Nutritional Status and Prevalence of Hypertension Among Industrial Migrant Workers in Chennai. Indian Journal Of Science And Technology. 2024;17:4711-21.

39 Shan G, Wei D, Wang C, Zhang J, Wang B, Ma M, et al. Trends of overweight and obesity in Yi people between 1996 and 2007: an Yi migrant study. Biomed Environ Sci. 2011;24:467-74.
40 Sornlorm K, Thi WM. Health literacy and high blood pressure among Myanmar migrant workers in Northeastern Thailand. PLoS One. 2024;19:e0302057.
41 Higgins JPT, Thomas J, Chandler J, Cumpston M, Li T, Page MJ, et al. Chapter 14: Completing 'Summary of findings' tables and grading the certainty of the evidence. 2024. Available: https://www.cochrane.org/authors/handbooks-and-manuals/handbook/current/chapter-14. Accessed.
